# Supplementary figures and images for: A data-driven prospective study of dementia among older adults in the United States
Source: PLoS One. 2020 Oct 7;15(10):e0239994. doi: 10.1371/journal.pone.0239994 (PMC7540891; doi:10.1371/journal.pone.0239994)

NH White Men

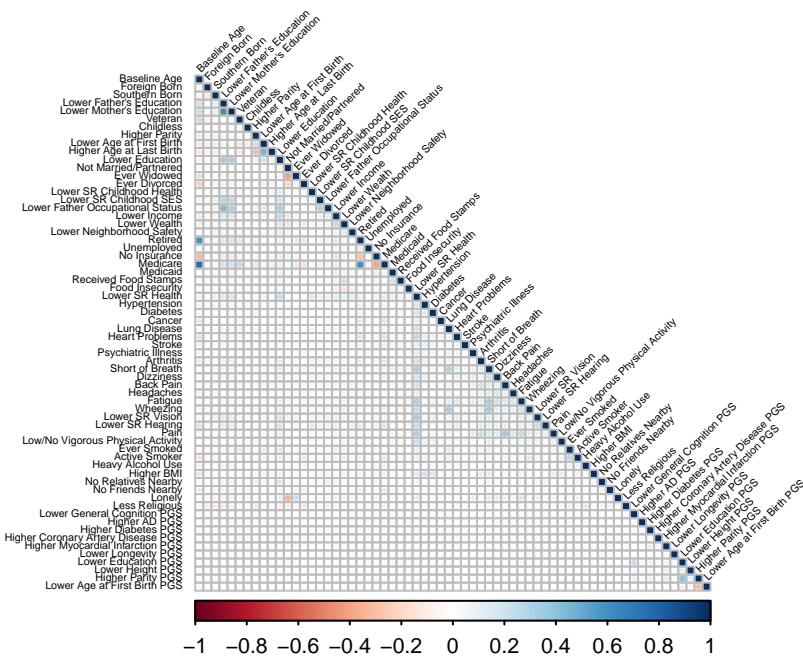

NH White Women

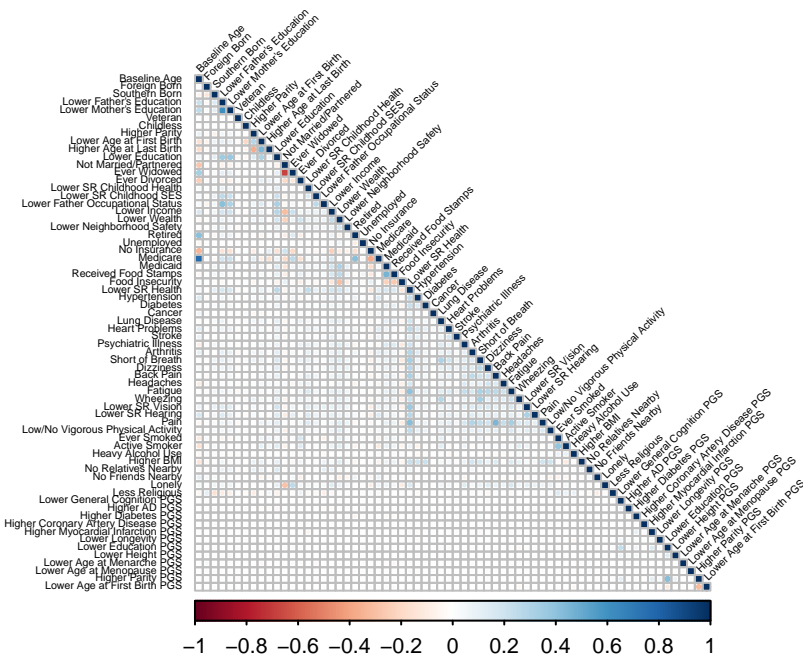

NH Black Men

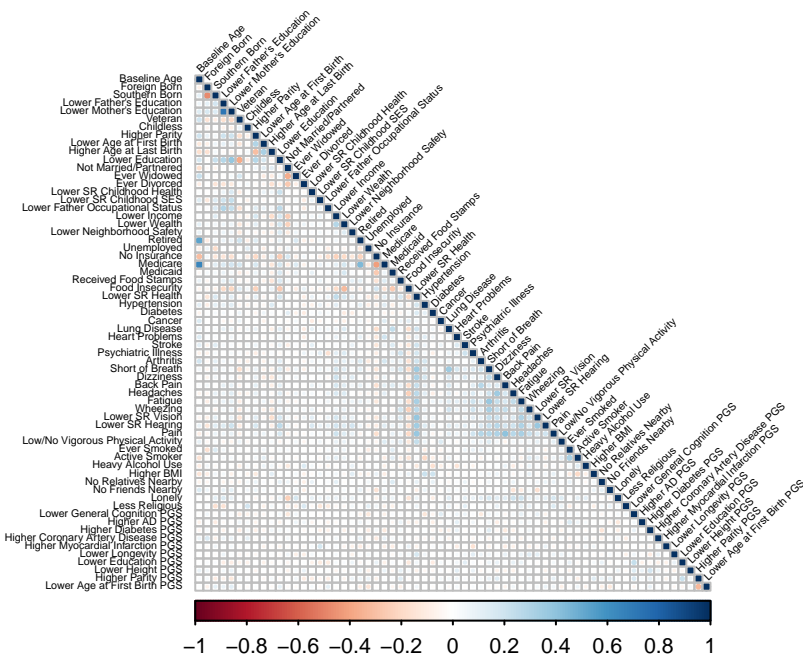

NH Black Women

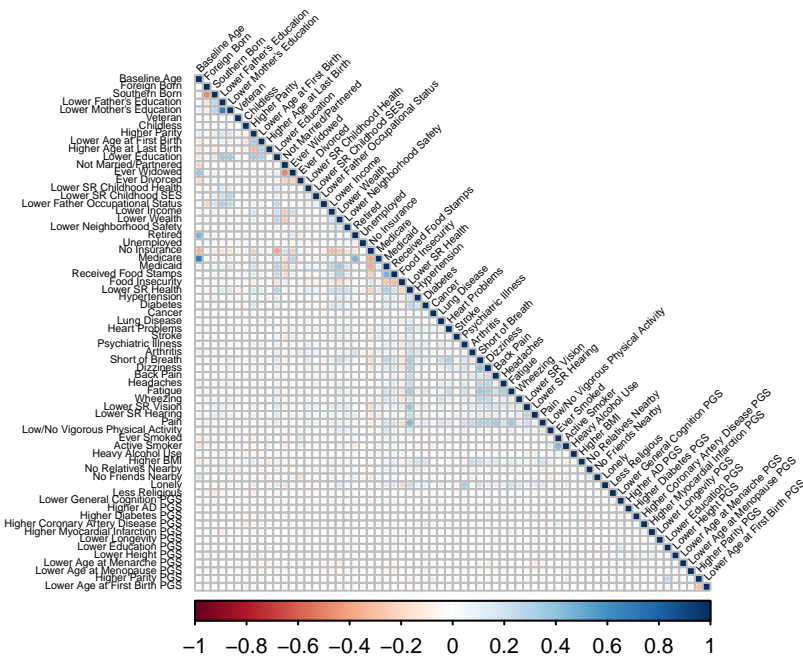

Supplement: S1 Fig — (PDF) [file pone.0239994.s001.pdf]

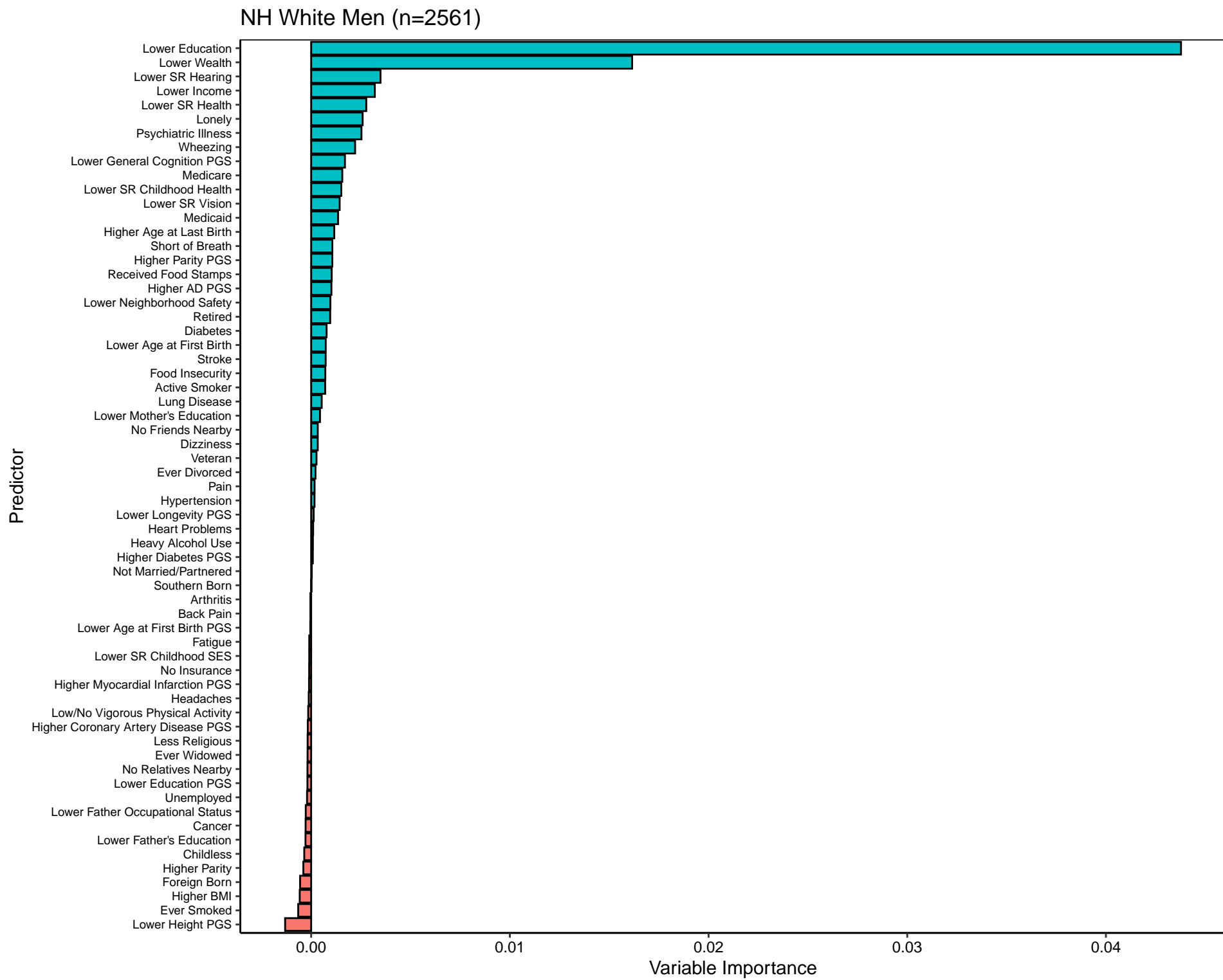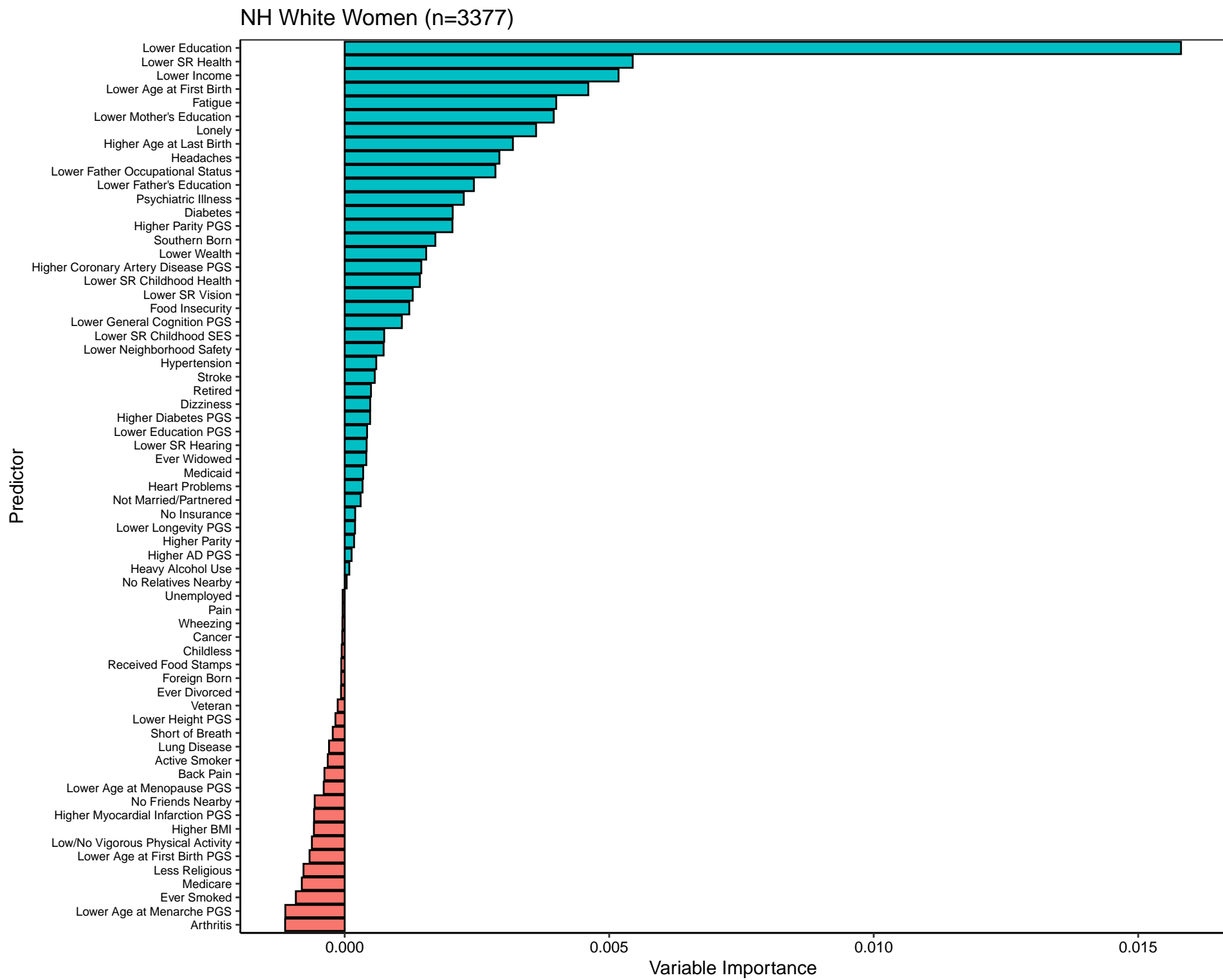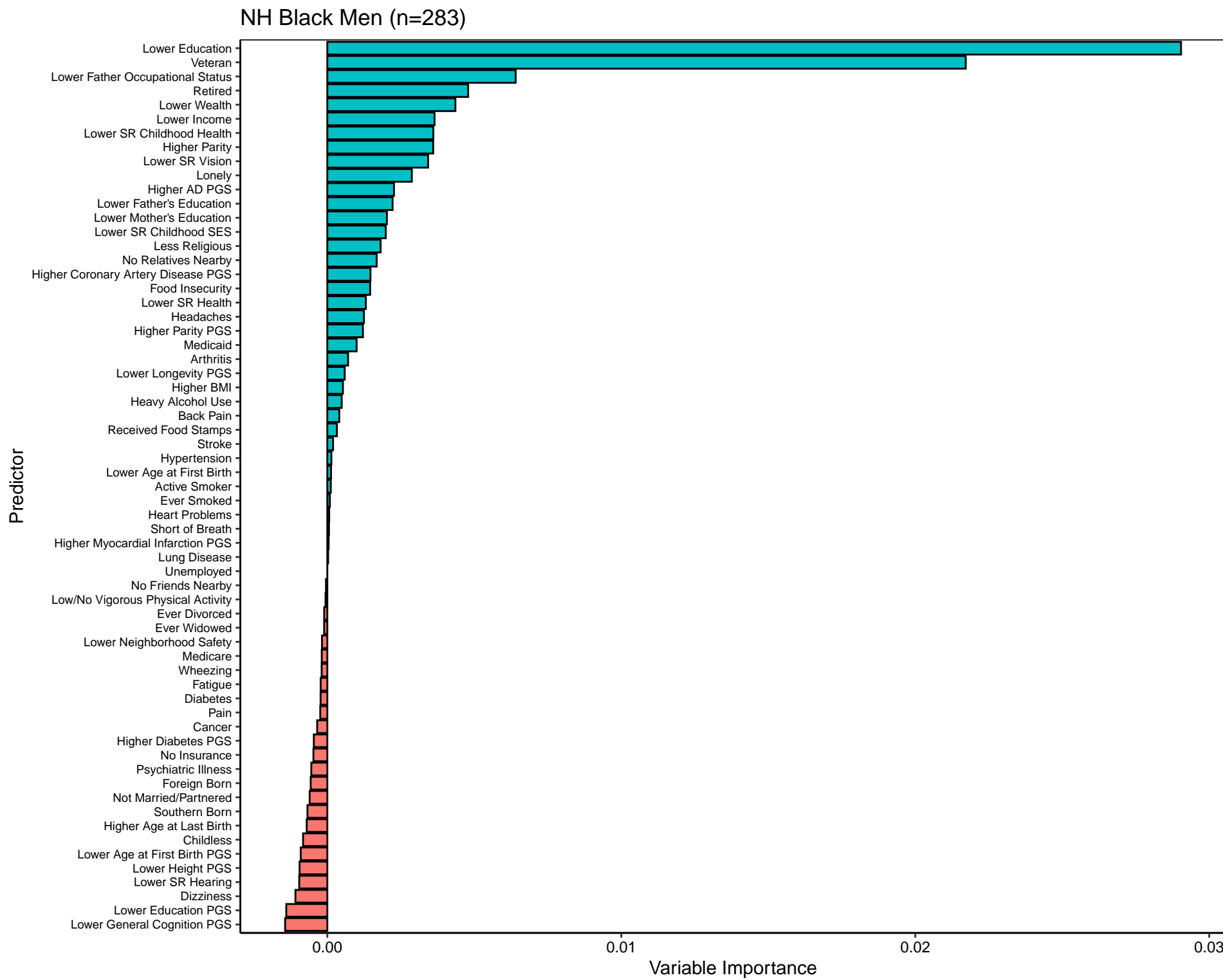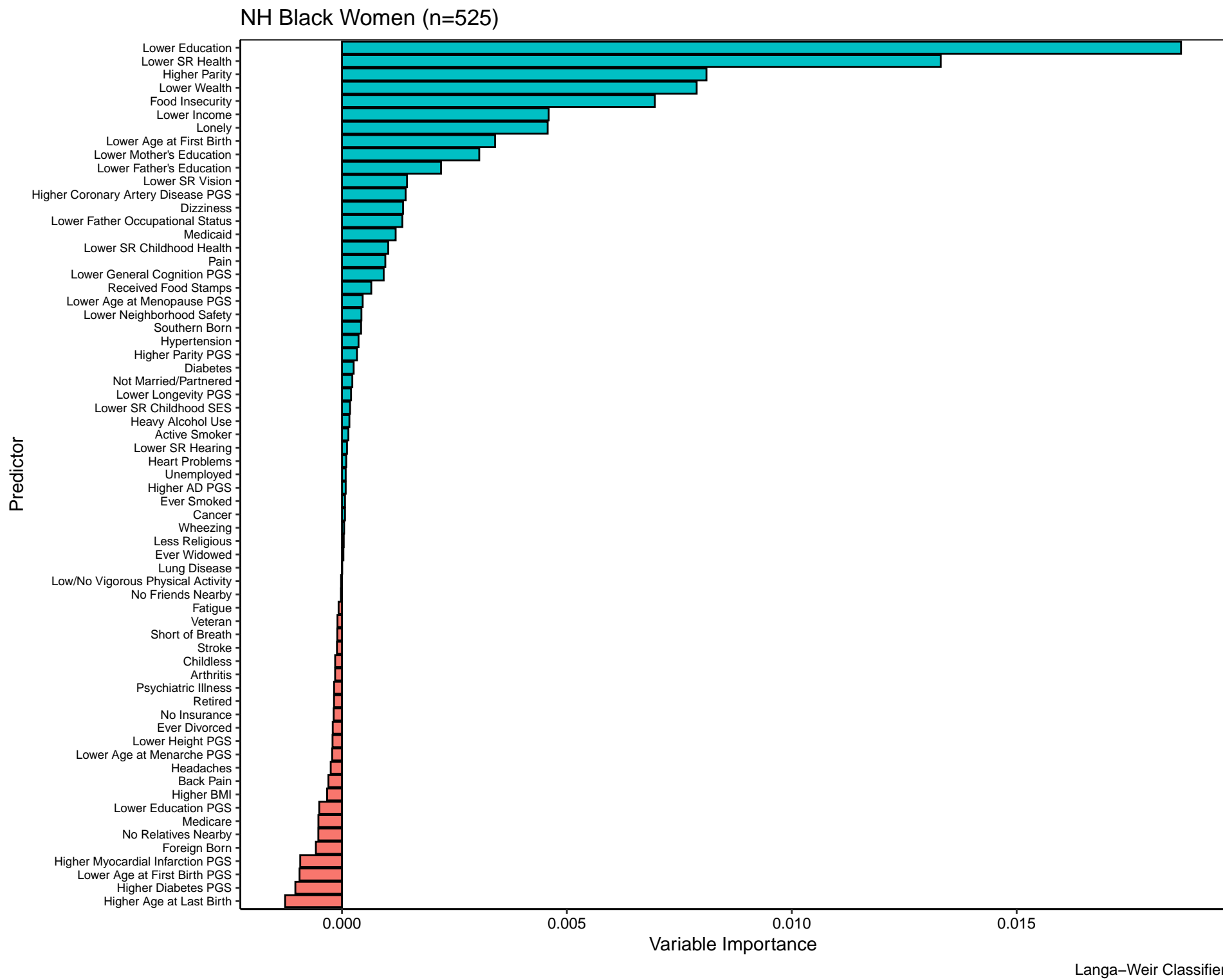

Supplement: S7 Fig — Model uses restricted analytic sample and classifies dementia using the Langa-Weir classification scheme. (PDF) [file pone.0239994.s007.pdf]

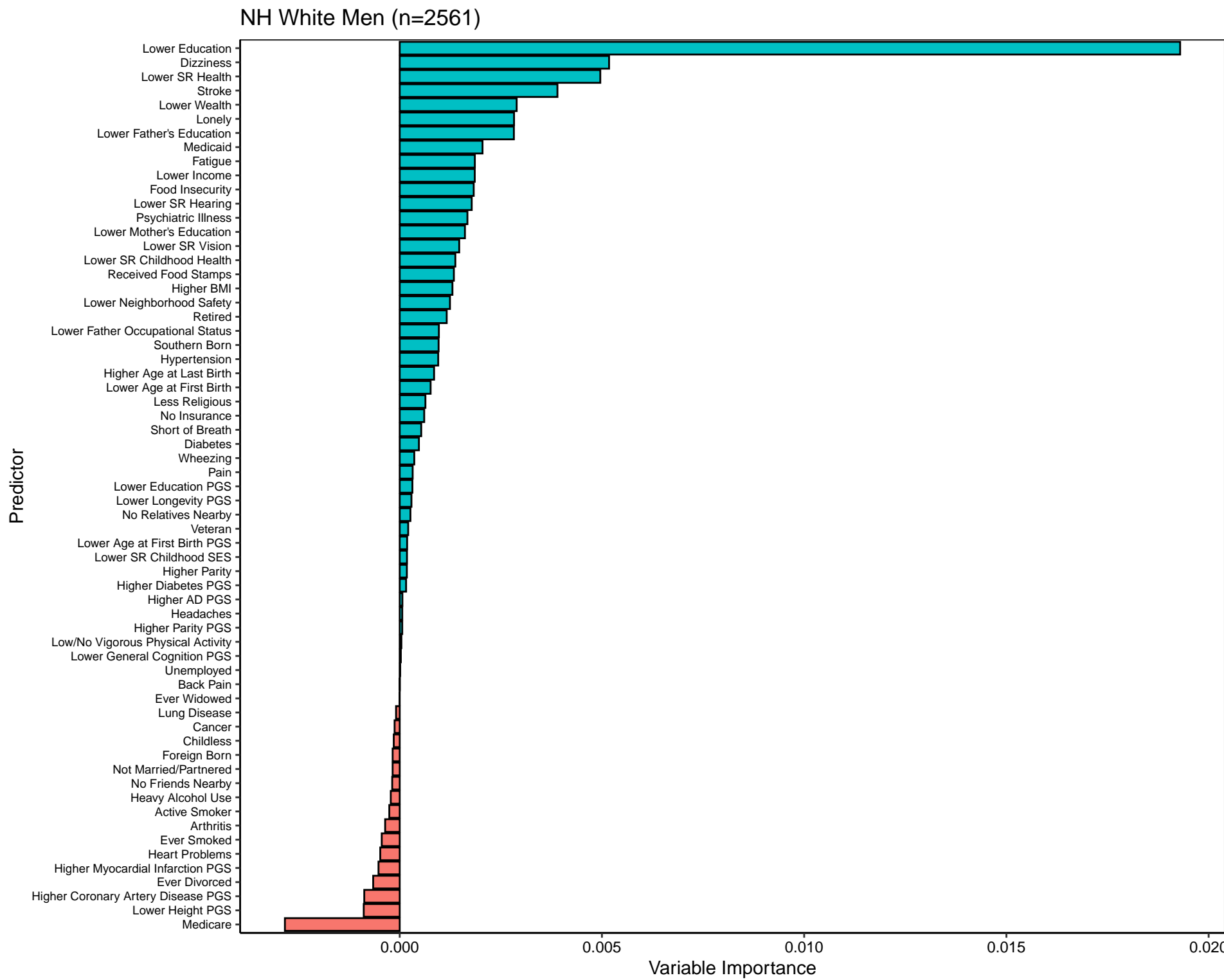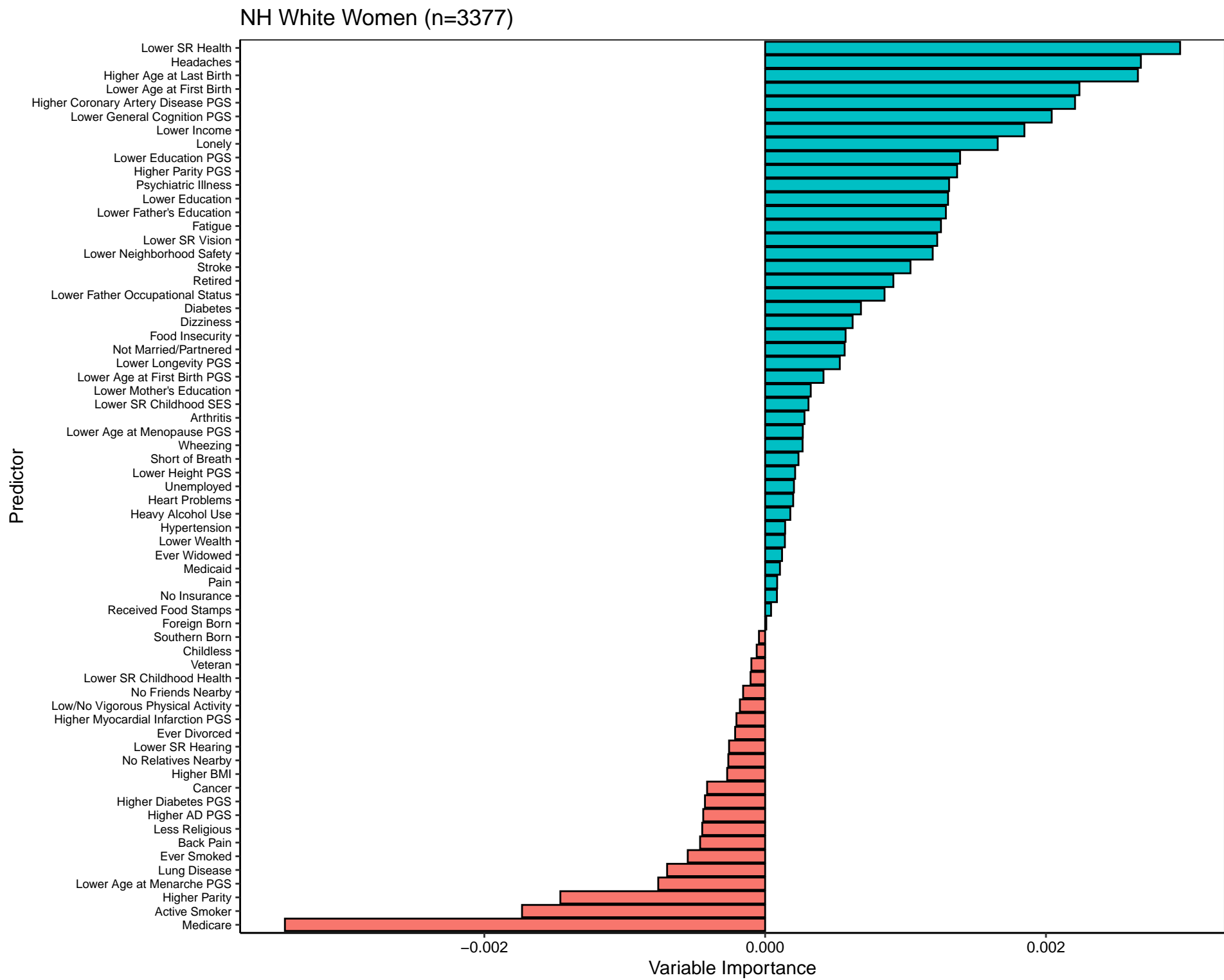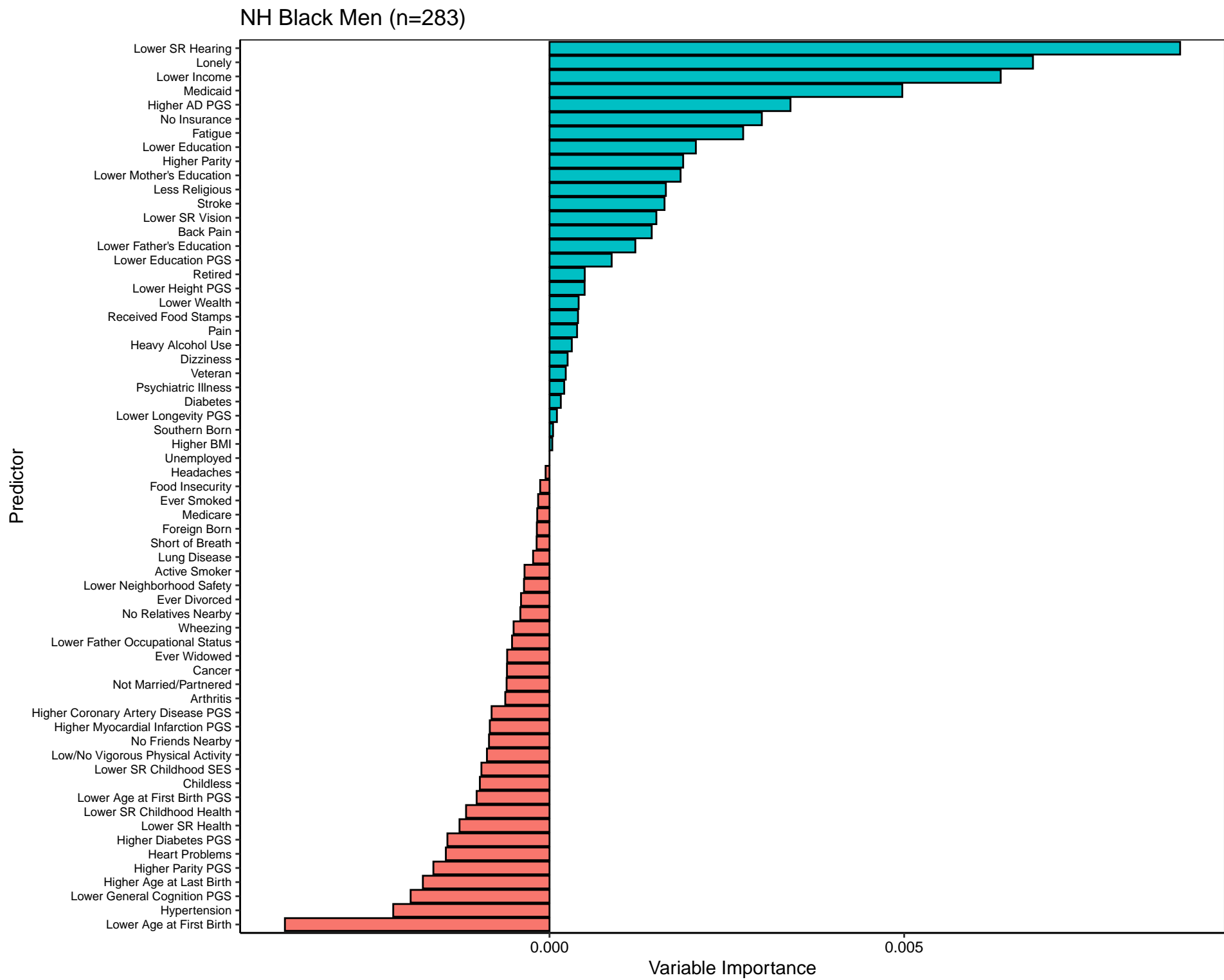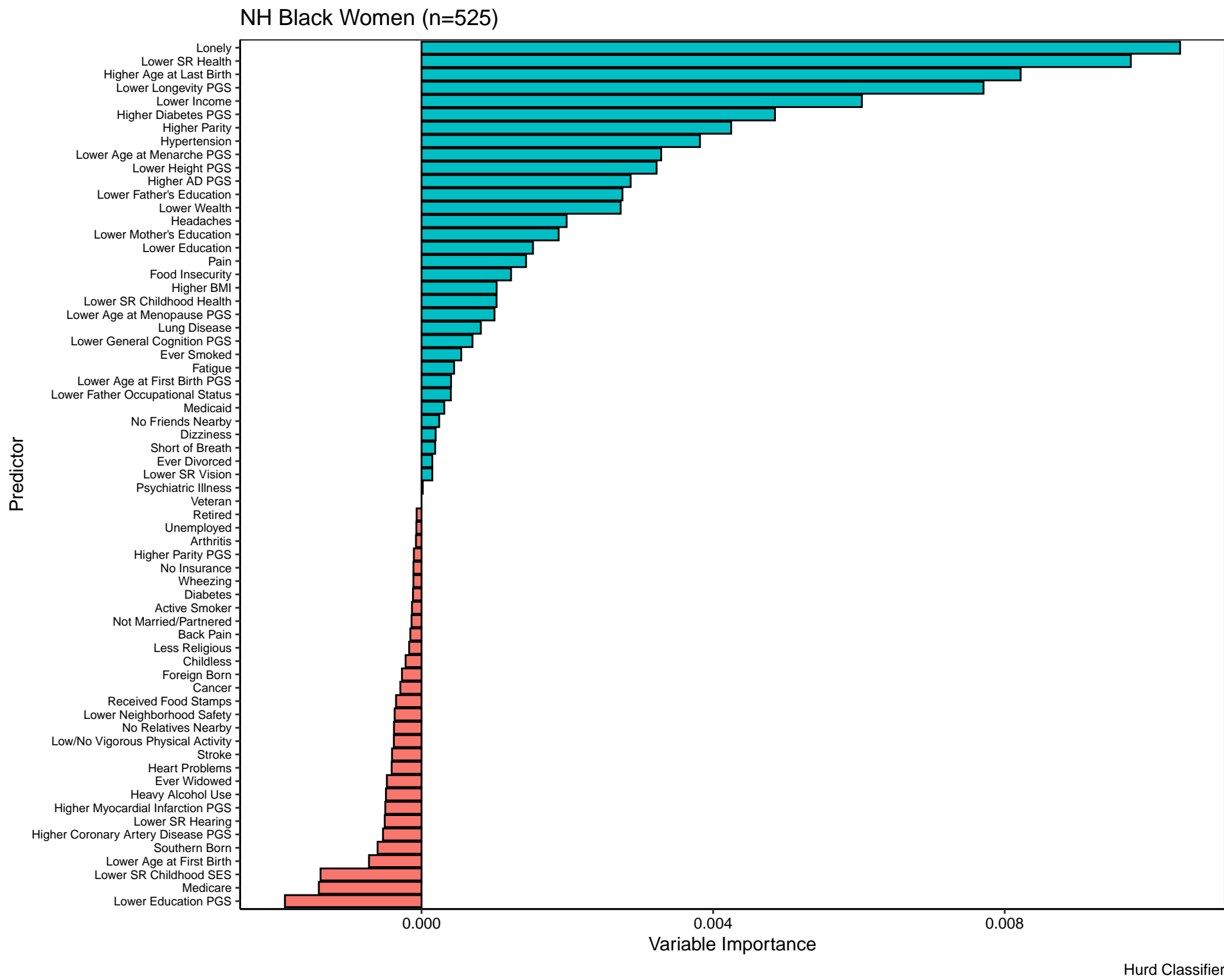

Supplement: S8 Fig — Model uses restricted analytic sample and classifies dementia using the Hurd classification scheme. (PDF) [file pone.0239994.s008.pdf]

NH White Men (n=2561)

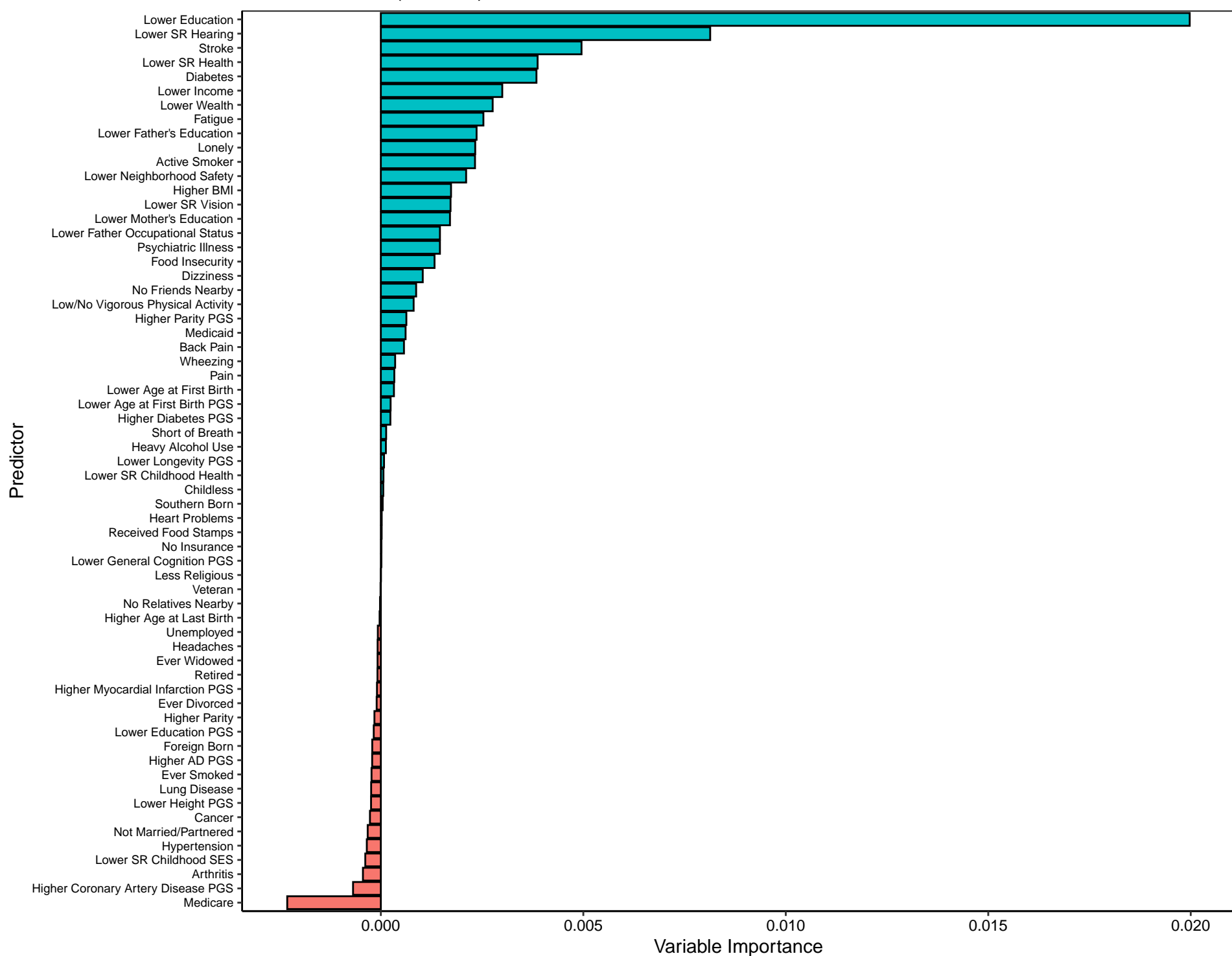

NH White Women (n=3377)

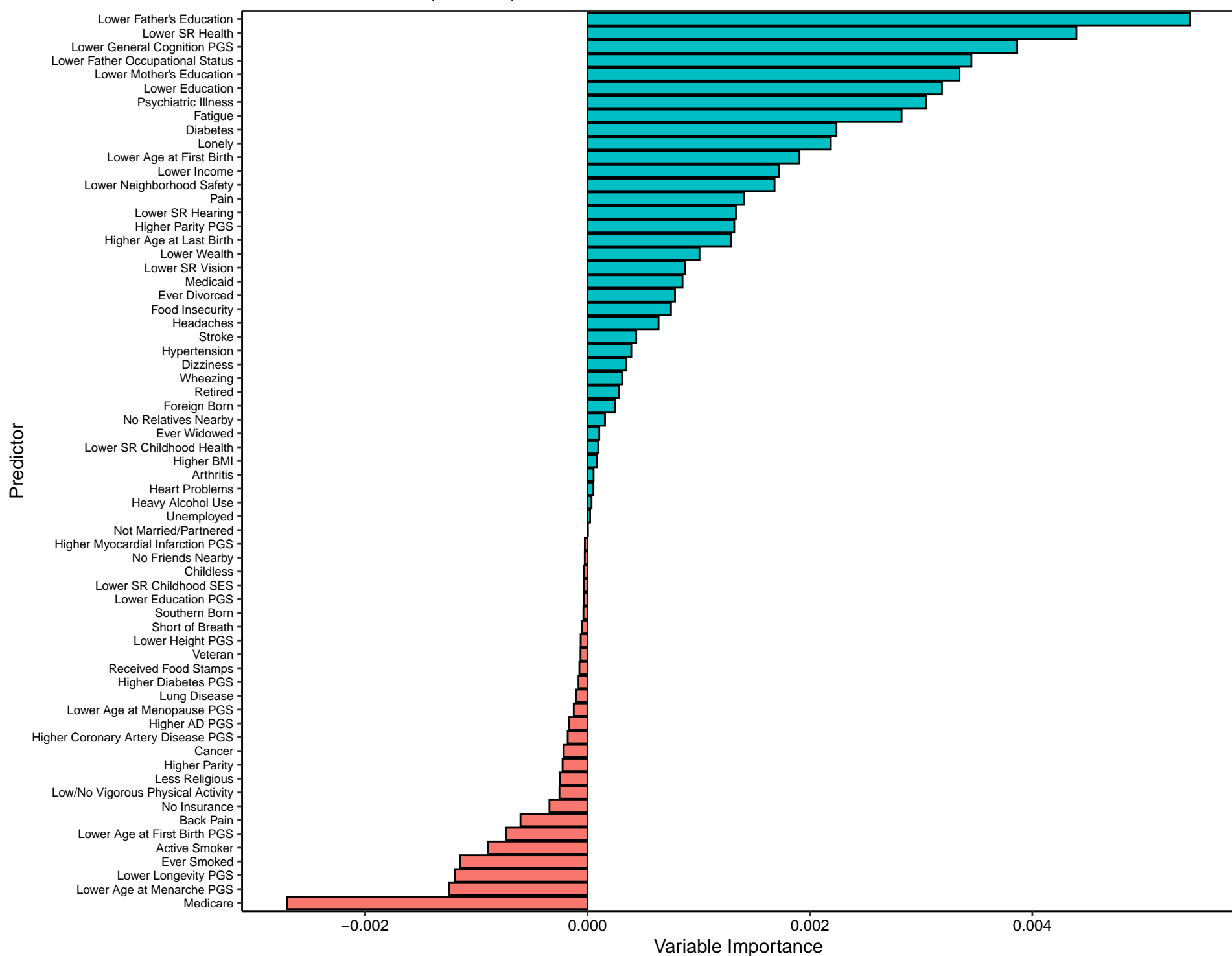

NH Black Men (n=283)

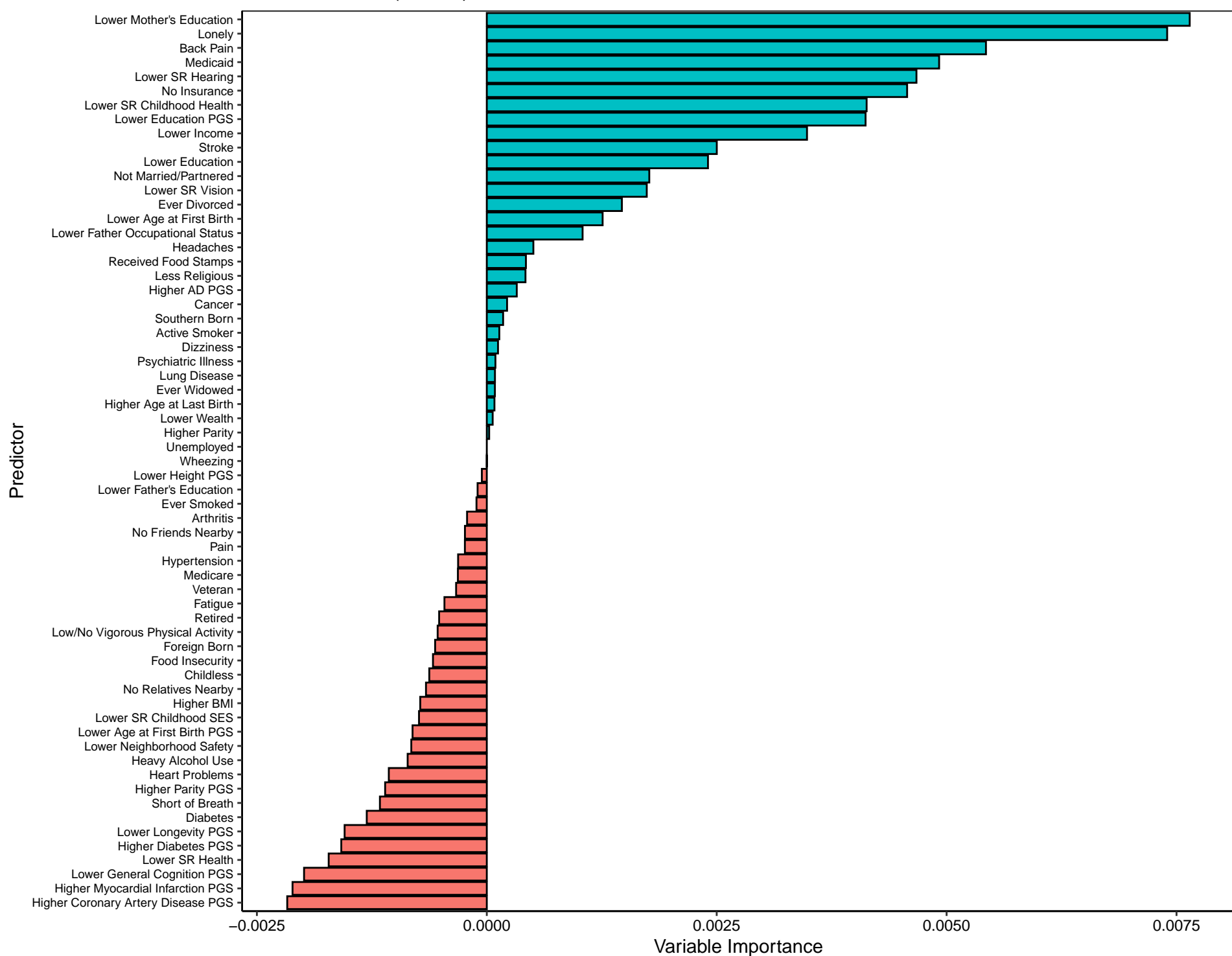

NH Black Women (n=525)

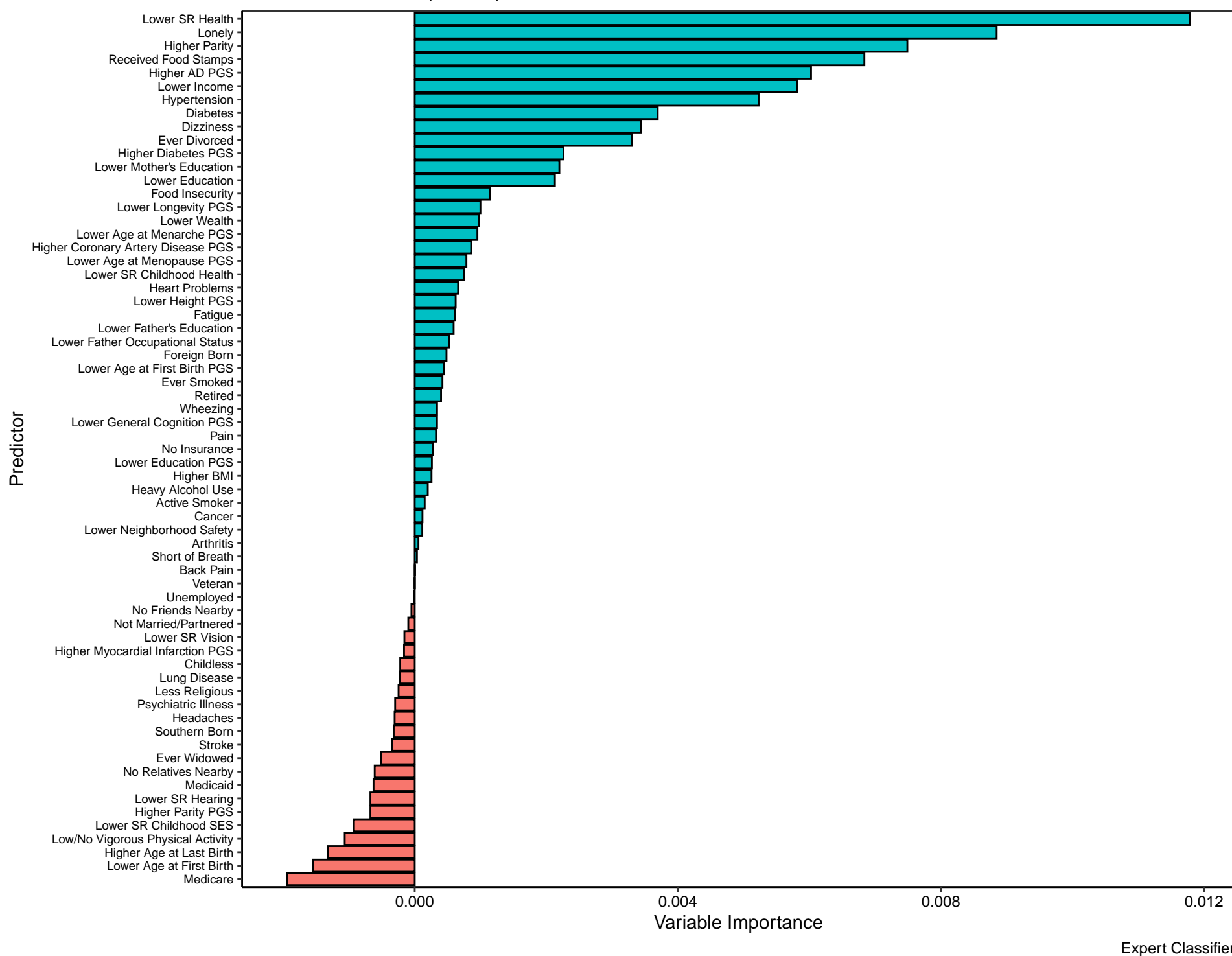

Supplement: S9 Fig — Model uses restricted analytic sample and classifies dementia using the Expert classification scheme. (PDF) [file pone.0239994.s009.pdf]

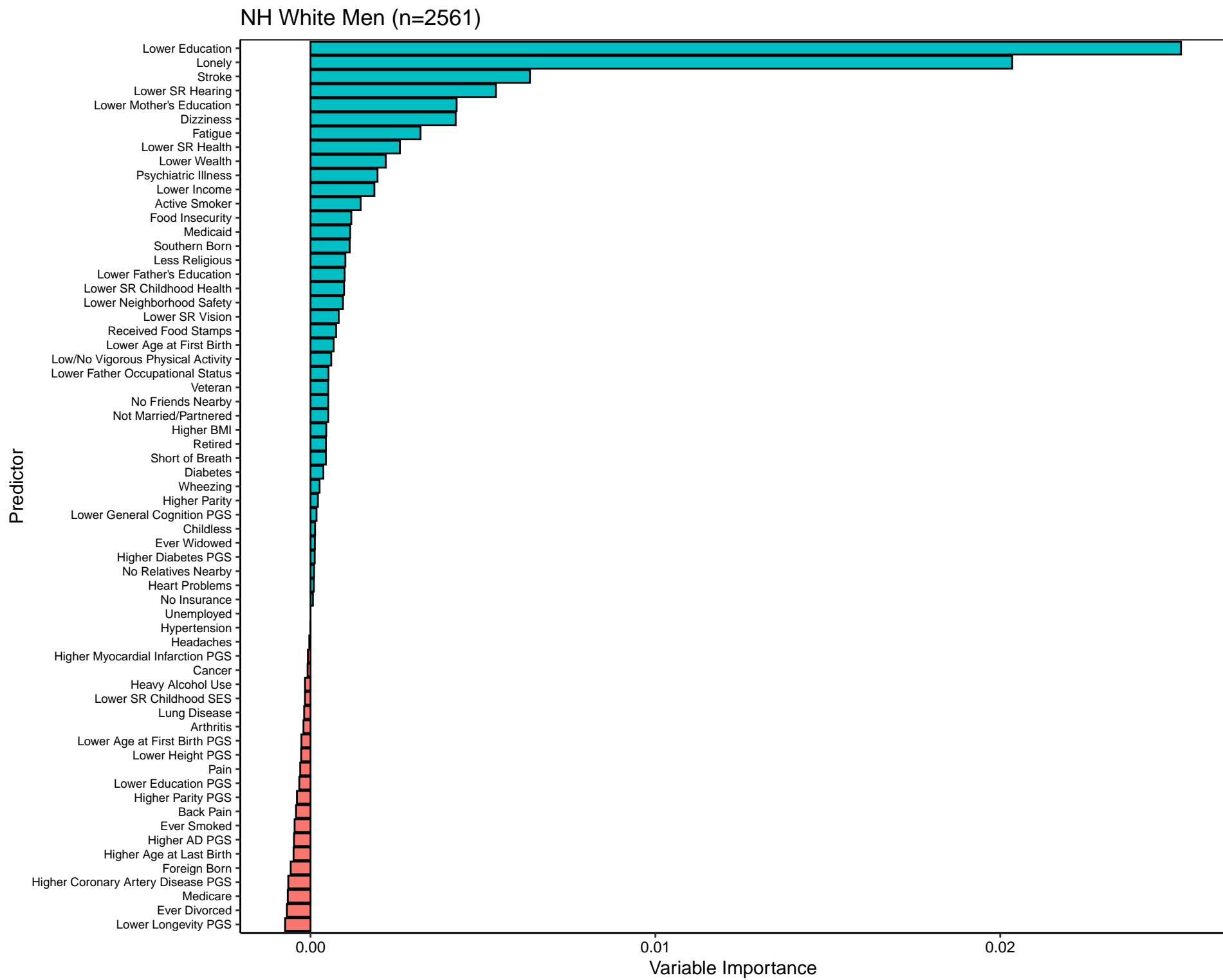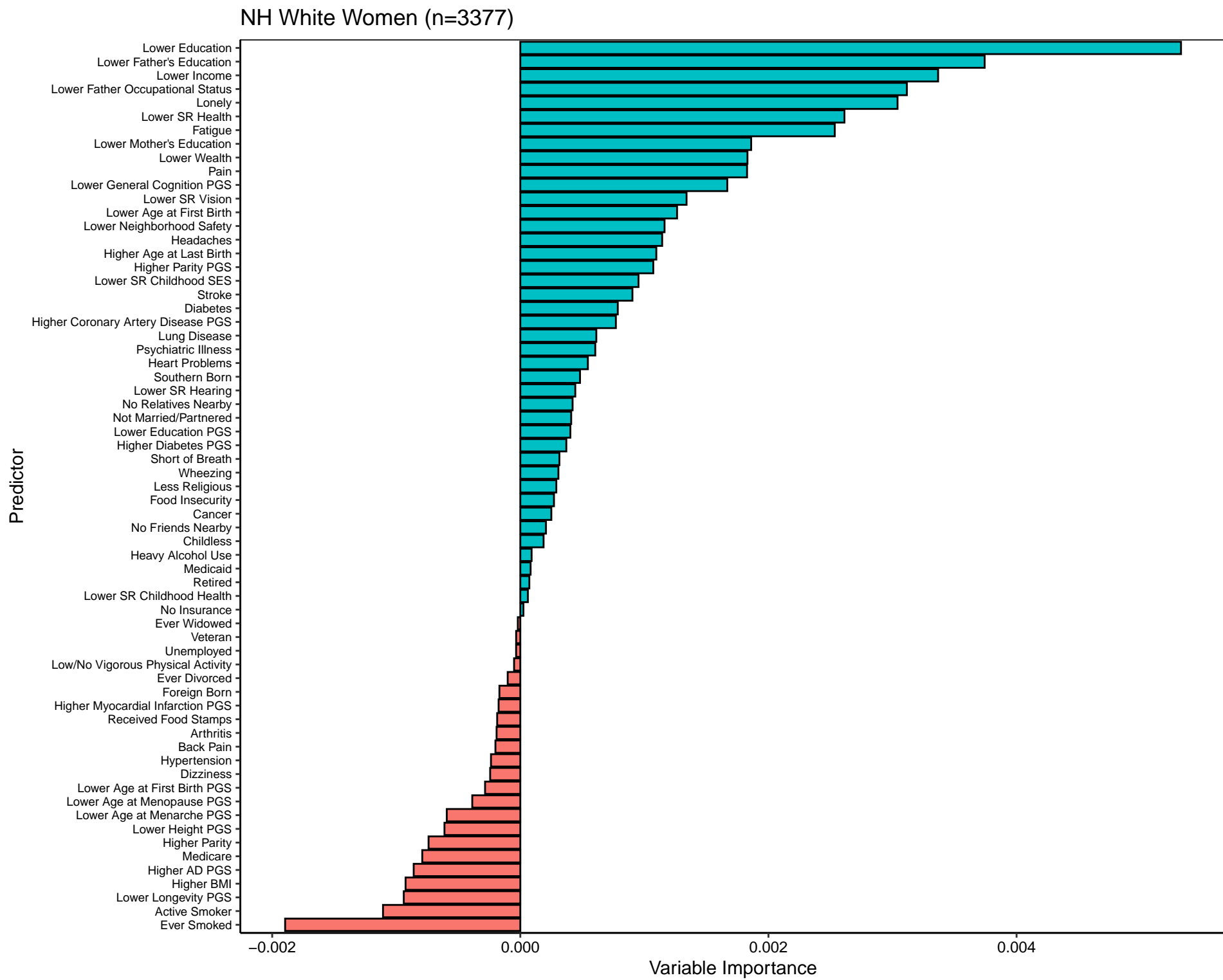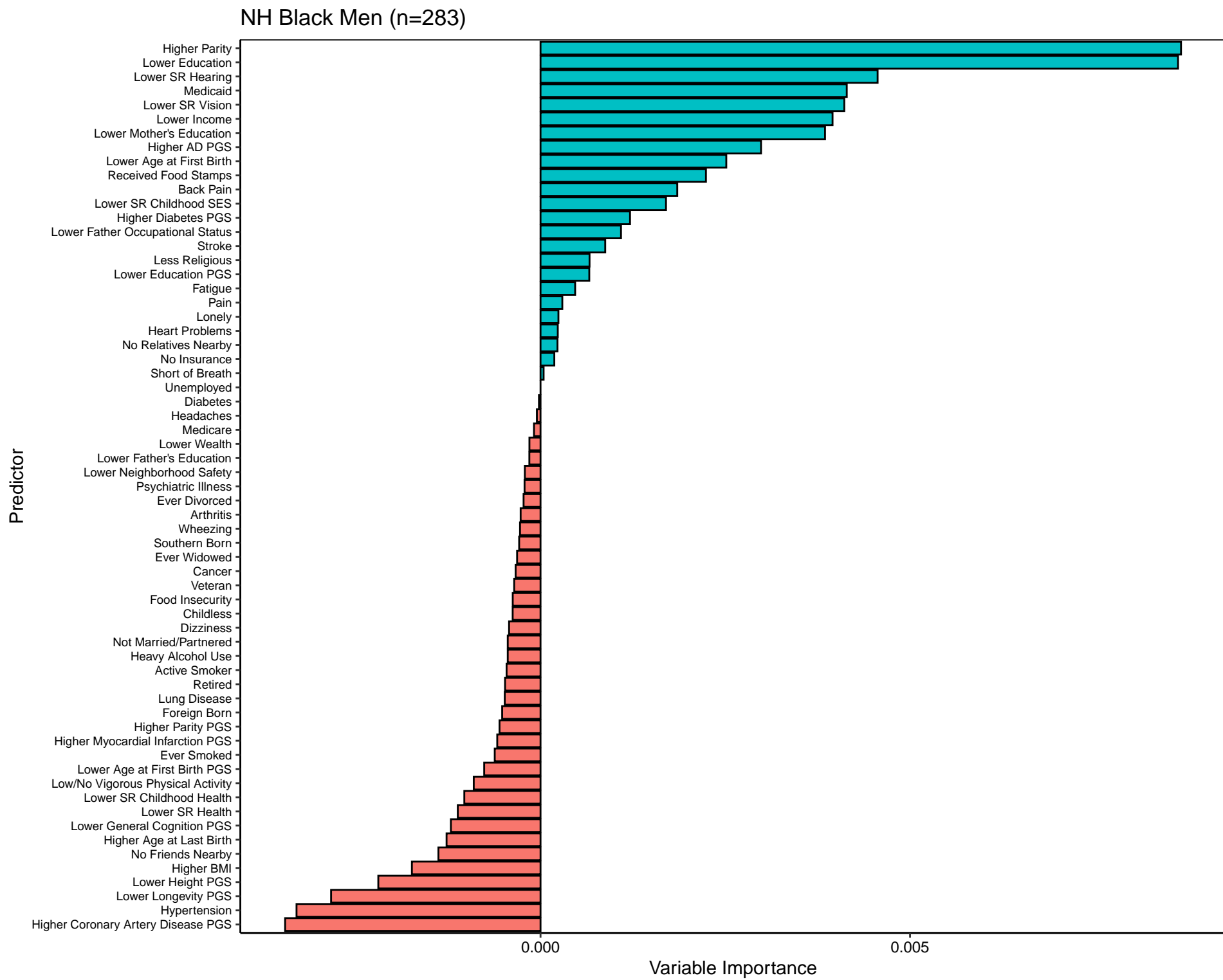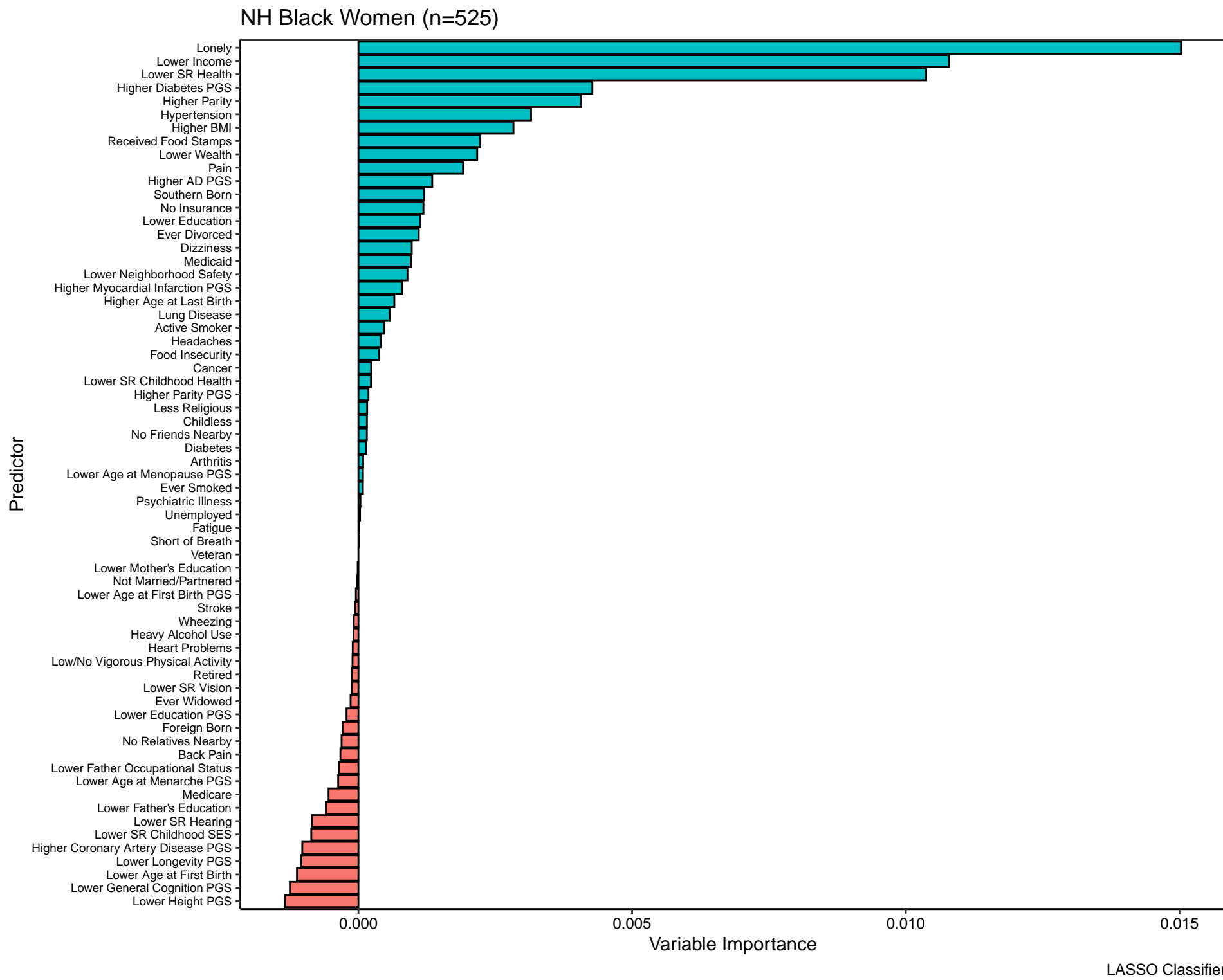

Supplement: S10 Fig — Model uses restricted analytic sample and classifies dementia using the LASSO classification scheme. (PDF) [file pone.0239994.s010.pdf]

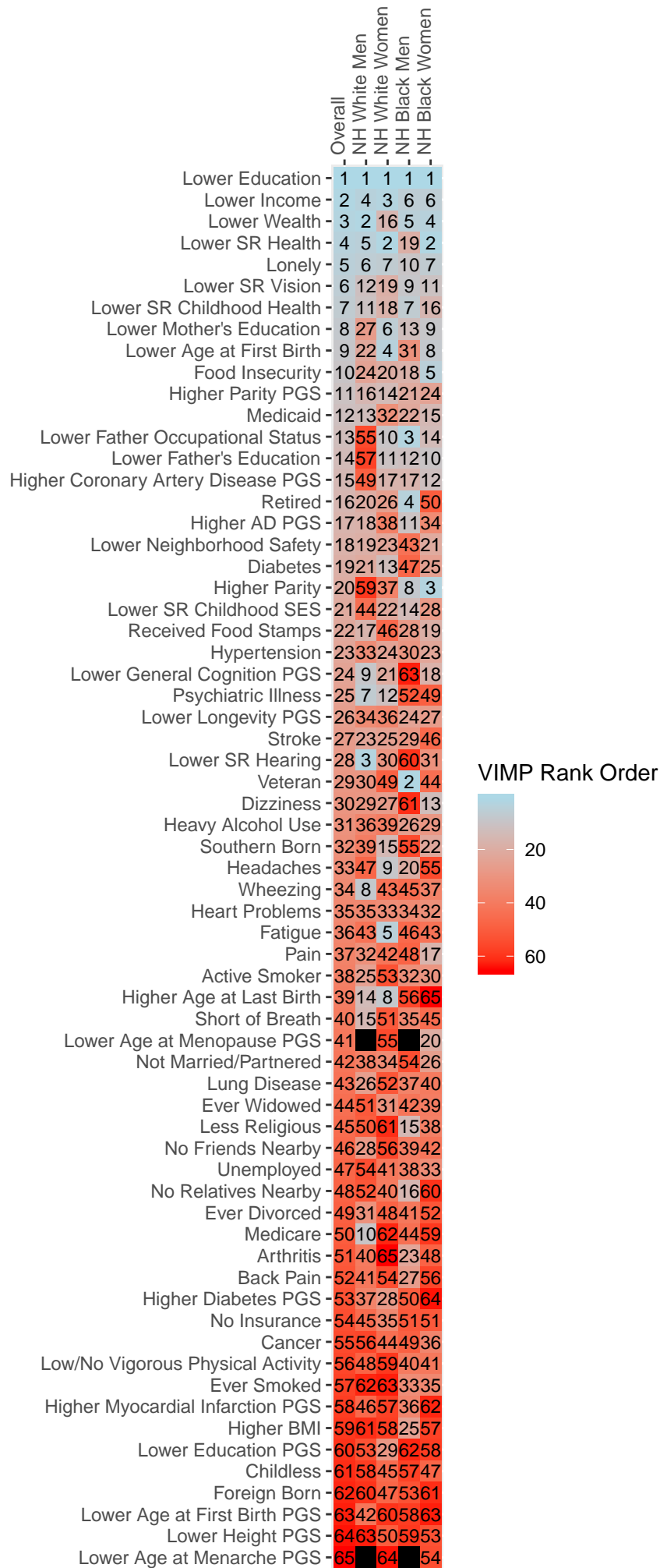

Supplement: S11 Fig — Model uses restricted analytic sample and classifies dementia using the Langa-Weir classification scheme. (PDF) [file pone.0239994.s011.pdf]

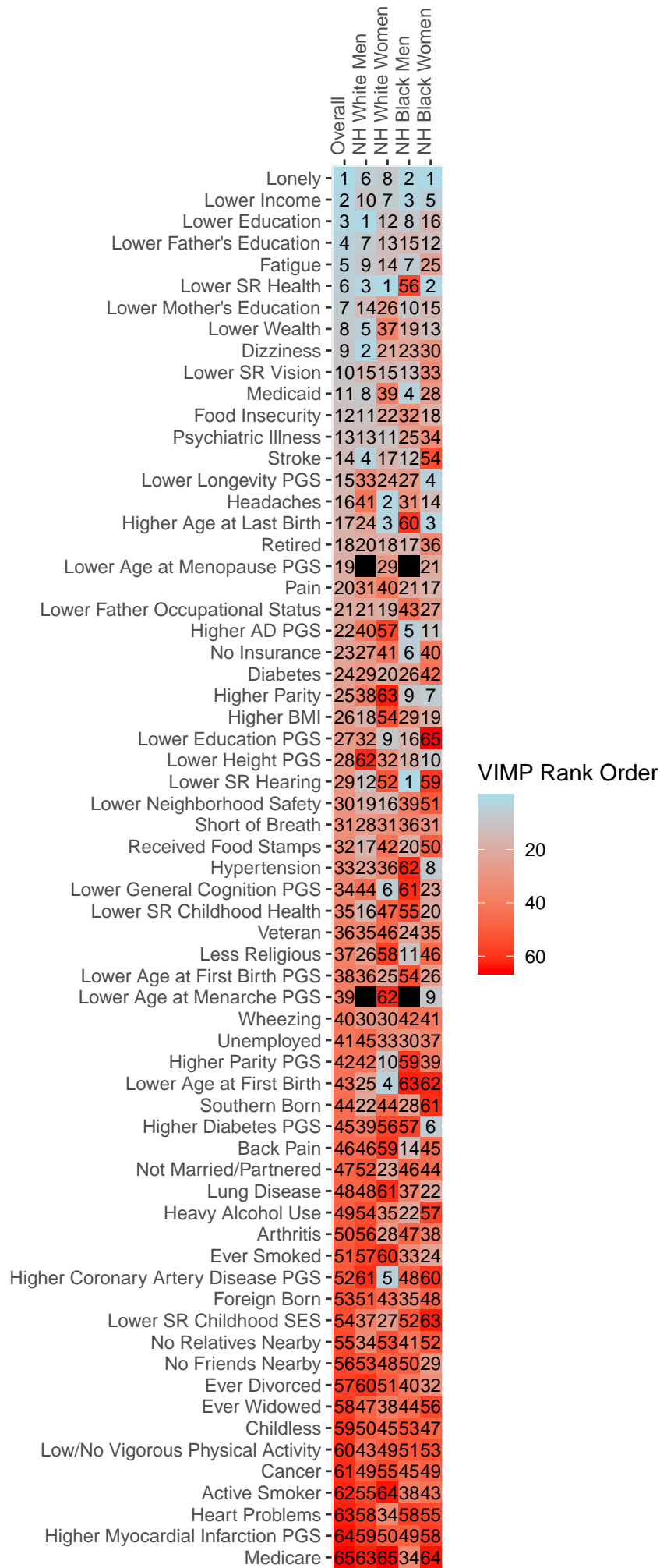

Supplement: S12 Fig — Model uses restricted analytic sample and classifies dementia using the Hurd classification scheme. (PDF) [file pone.0239994.s012.pdf]
