# Supplementary material for: A data-driven prospective study of dementia among older adults in the United States
Source: PLoS One. 2020 Oct 7;15(10):e0239994. doi: 10.1371/journal.pone.0239994 (PMC7540891; doi:10.1371/journal.pone.0239994)
Supplement: S2 Fig — Model uses full analytic sample and classifies dementia using the Langa-Weir classification scheme. (PDF) [file pone.0239994.s002.pdf]

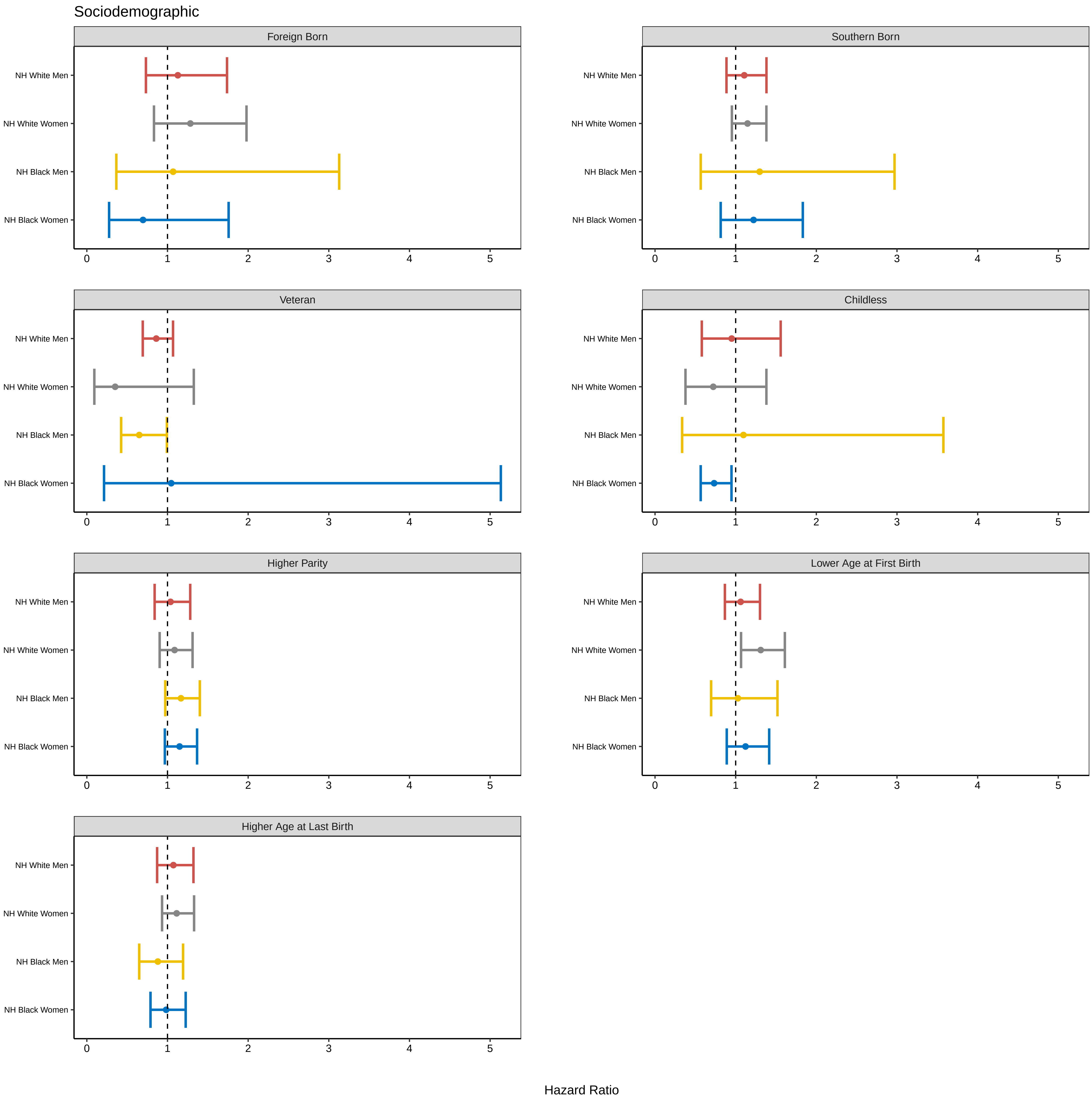

Sociodemographic
Foreign Born
Southern Born
Veteran
Childless
Higher Parity
Lower Age at First Birth
Higher Age at Last Birth
NH White Men
NH White Women
NH Black Men
NH Black Women
0
1
2
3
4
5
Hazard Ratio

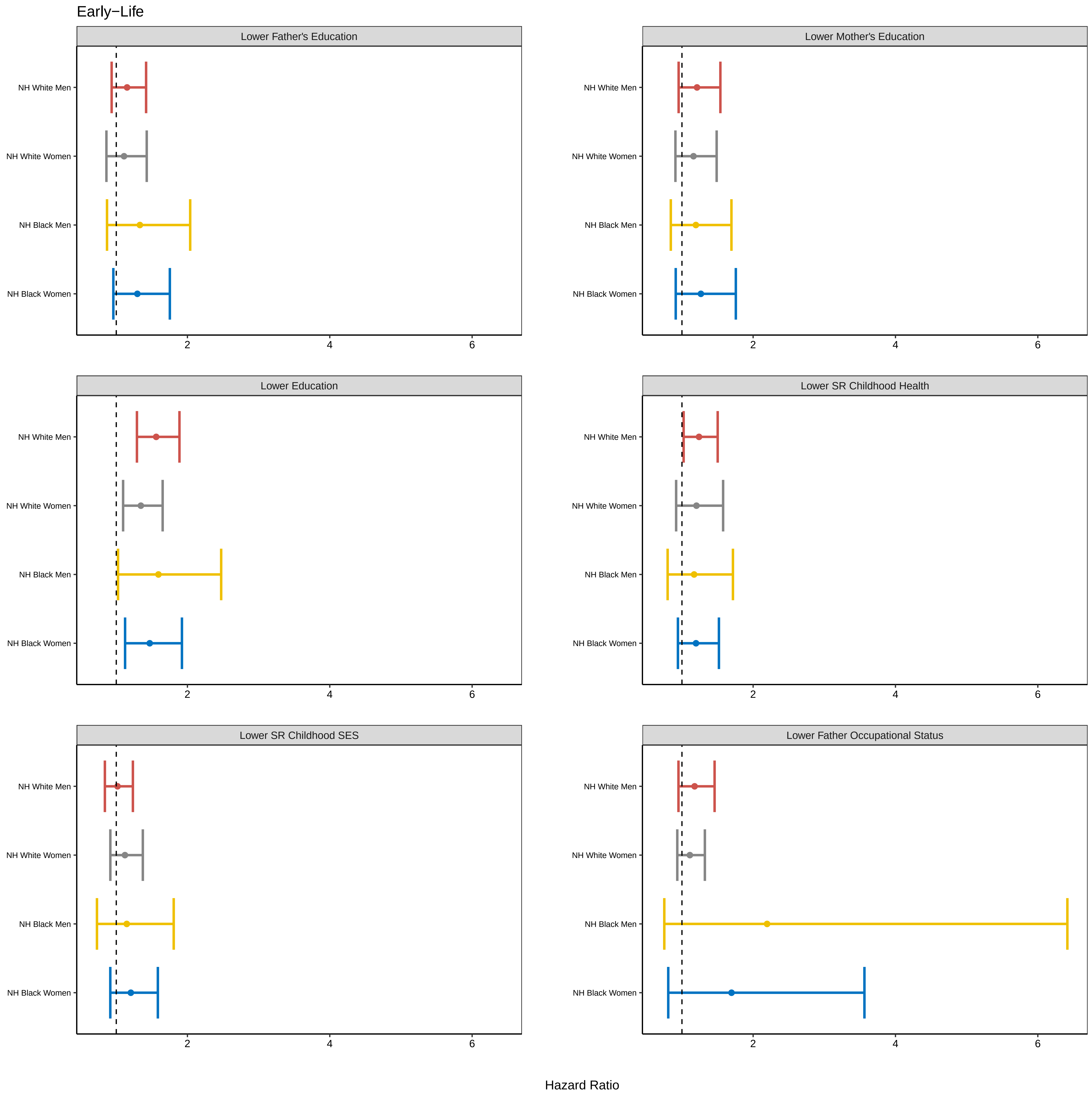
Early−Life
Lower Father's Education
Lower Mother's Education
Lower Education
Lower SR Childhood Health
Lower SR Childhood SES
Lower Father Occupational Status
NH White Men
NH White Women
NH Black Men
NH Black Women
2
4
6
Hazard Ratio

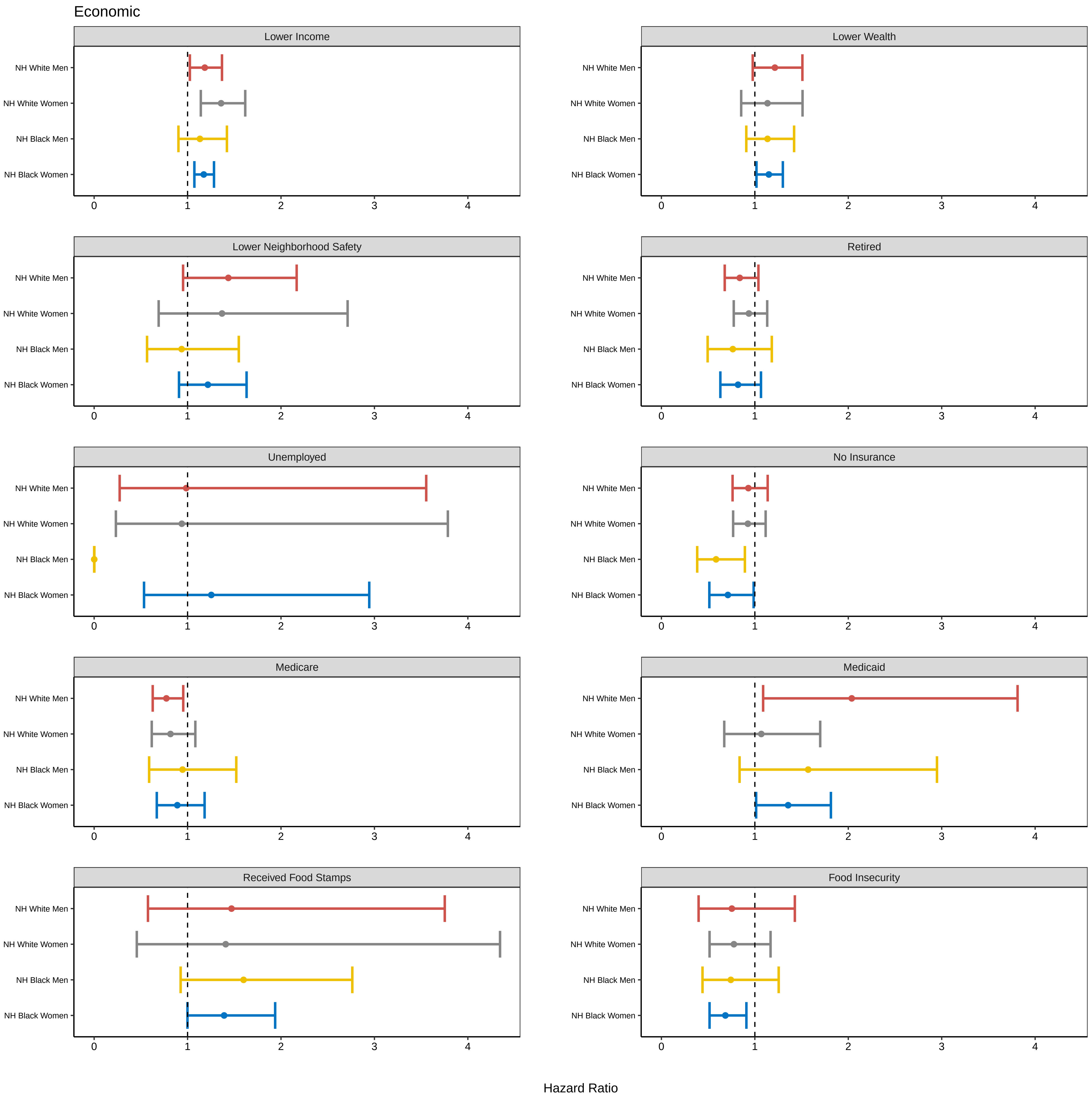
Economic
Lower Income
Lower Wealth
Lower Neighborhood Safety
Retired
Unemployed
No Insurance
Medicare
Medicaid
Received Food Stamps
Food Insecurity
NH White Men
NH White Women
NH Black Men
NH Black Women
0
1
2
3
4
Hazard Ratio

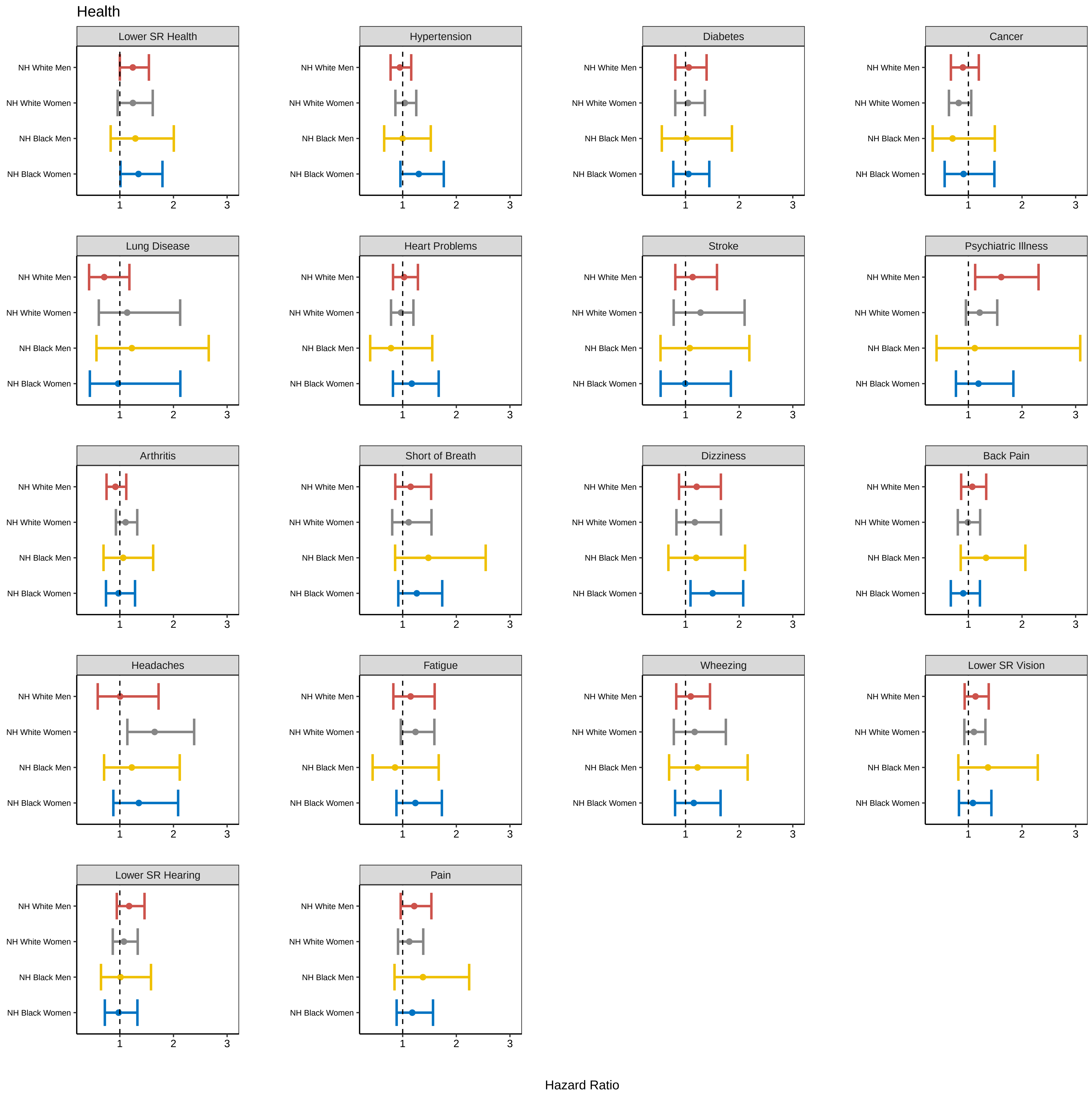
Health
Lower SR Health
Hypertension
Diabetes
Cancer
Lung Disease
Heart Problems
Stroke
Psychiatric Illness
Arthritis
Short of Breath
Dizziness
Back Pain
Headaches
Fatigue
Wheezing
Lower SR Vision
Lower SR Hearing
Pain
NH White Men
NH White Women
NH Black Men
NH Black Women
1
2
3
Hazard Ratio

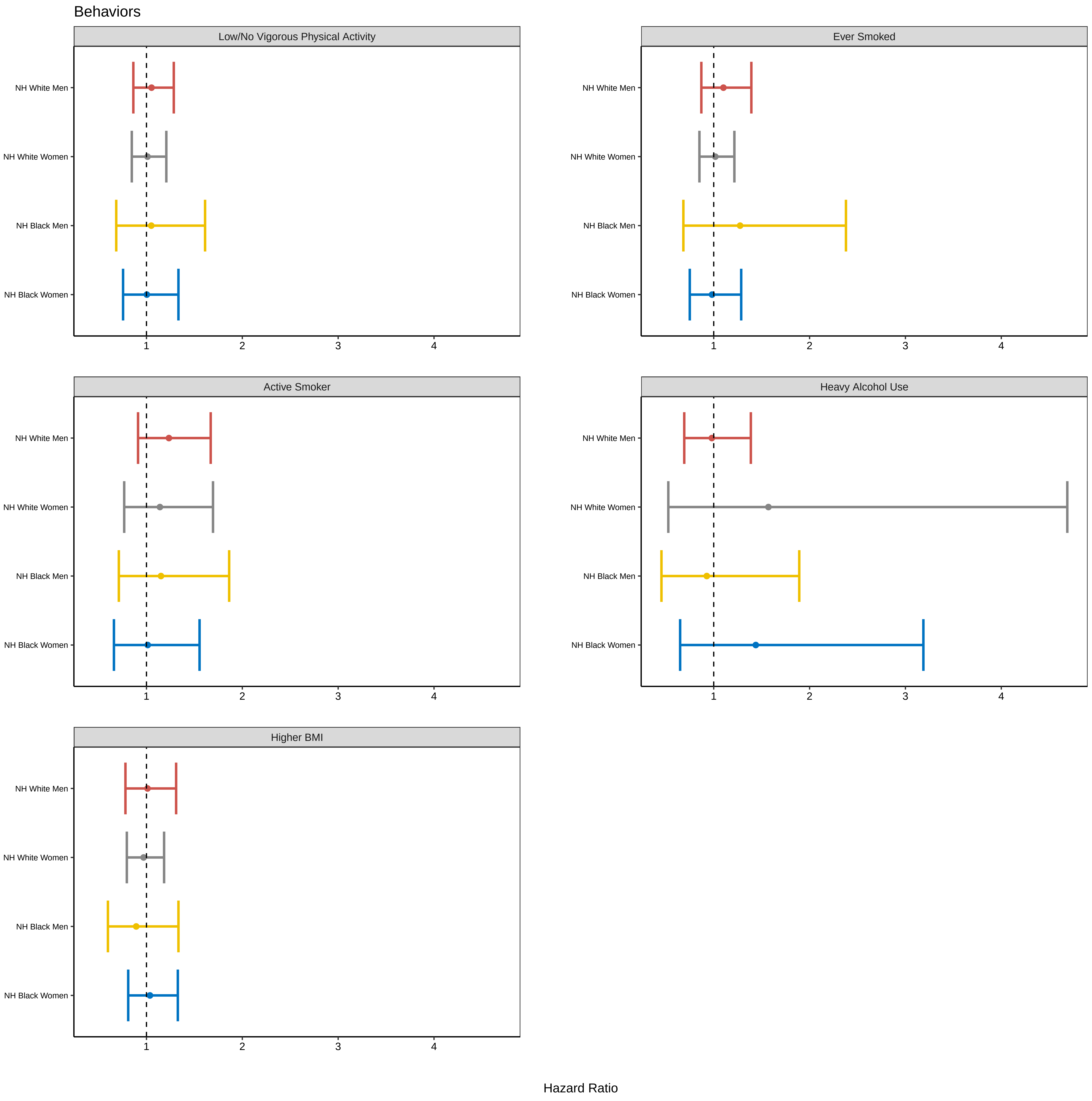

Behaviors
Low/No Vigorous Physical Activity
Ever Smoked
Active Smoker
Heavy Alcohol Use
Higher BMI
NH White Men
NH White Women
NH Black Men
NH Black Women
1
2
3
4
Hazard Ratio

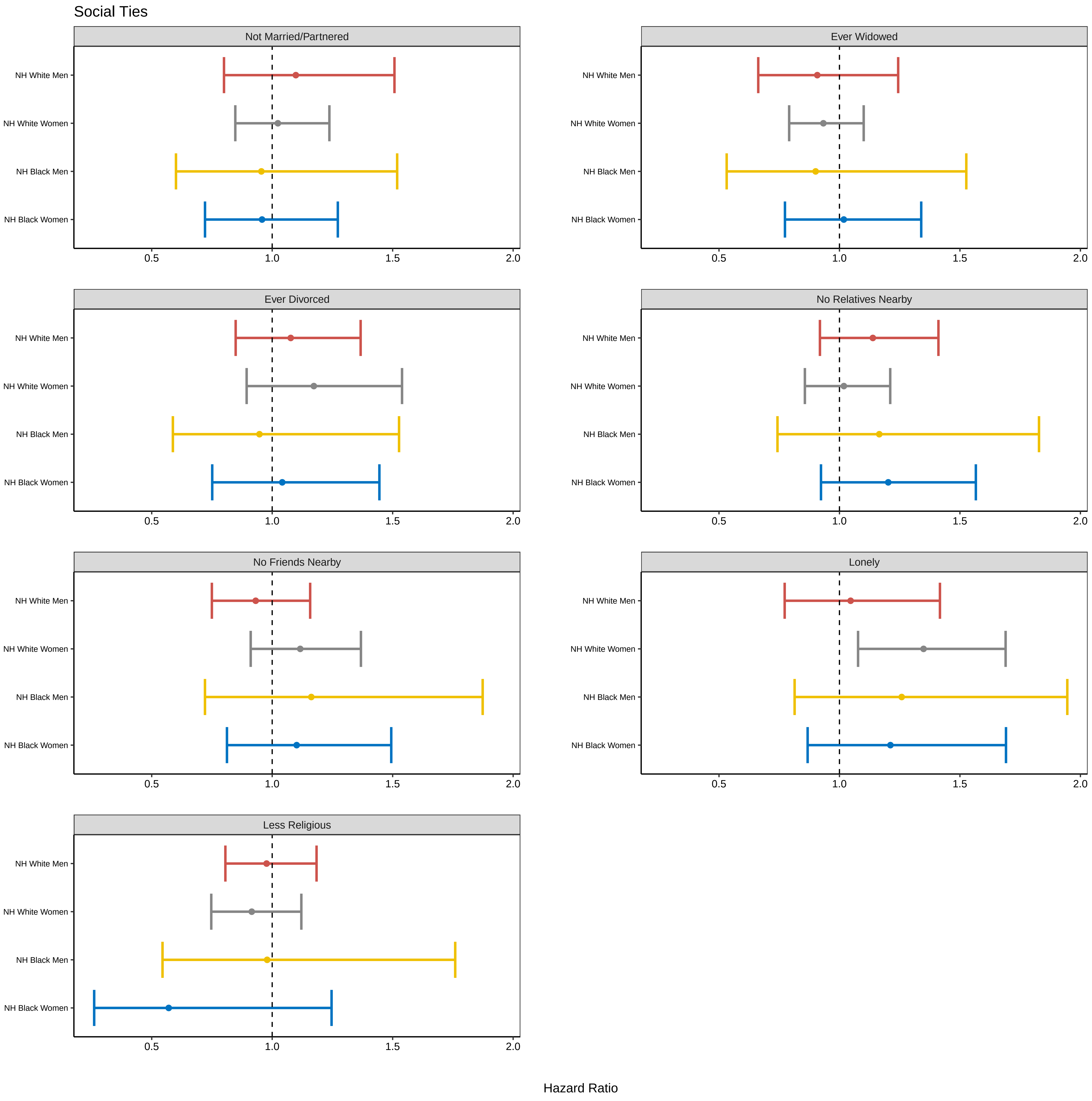

Social Ties
Not Married/Partnered
Ever Widowed
Ever Divorced
No Relatives Nearby
No Friends Nearby
Lonely
Less Religious
NH White Men
NH White Women
NH Black Men
NH Black Women
0.5
1.0
1.5
2.0
Hazard Ratio

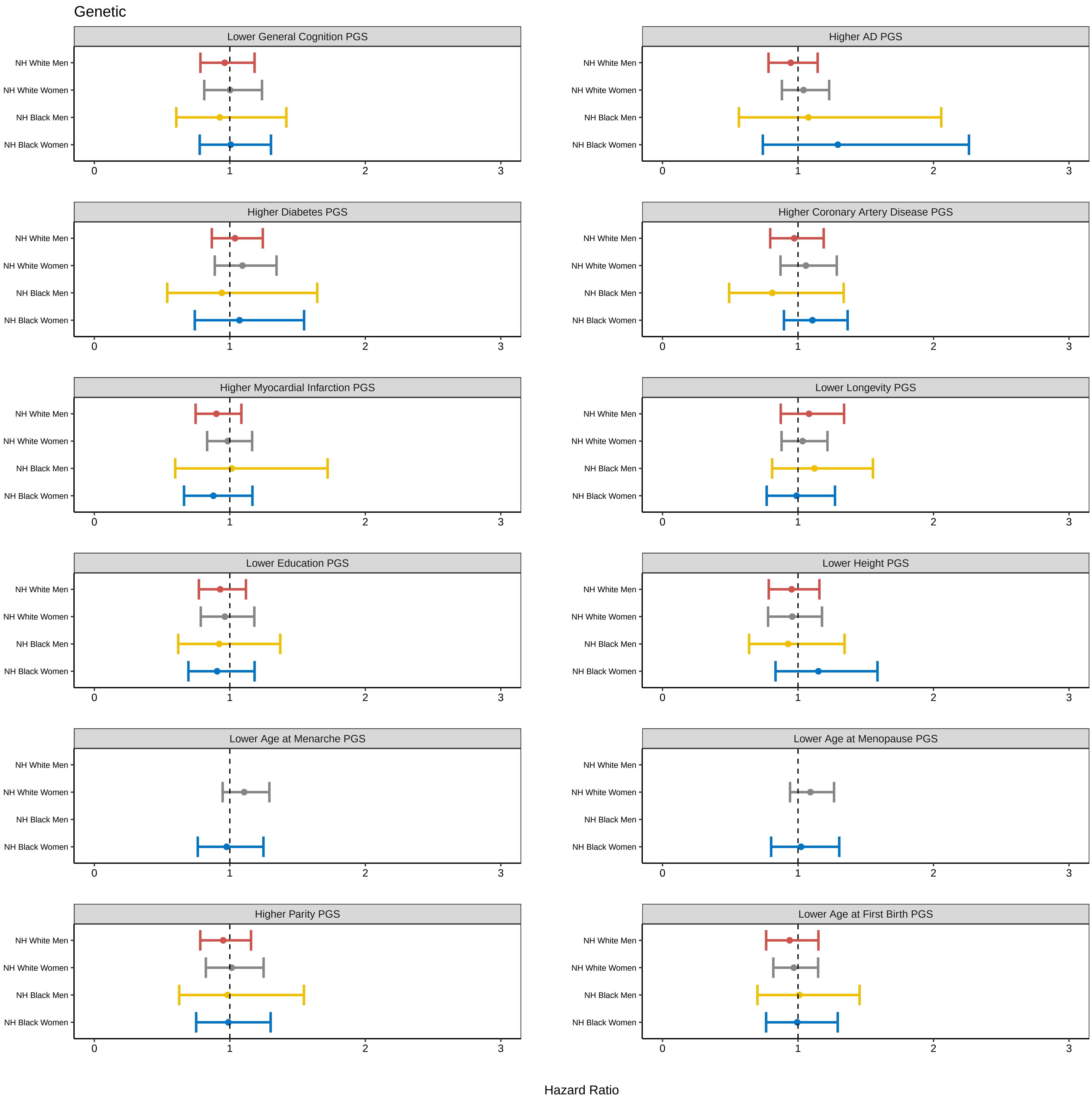
Genetic
Lower General Cognition PGS
Higher AD PGS
Higher Diabetes PGS
Higher Coronary Artery Disease PGS
Higher Myocardial Infarction PGS
Lower Longevity PGS
Lower Education PGS
Lower Height PGS
Lower Age at Menarche PGS
Lower Age at Menopause PGS
Higher Parity PGS
Lower Age at First Birth PGS
NH White Men
NH White Women
NH Black Men
NH Black Women
0
1
2
3
Hazard Ratio
