# Supplementary material for: A data-driven prospective study of dementia among older adults in the United States
Source: PLoS One. 2020 Oct 7;15(10):e0239994. doi: 10.1371/journal.pone.0239994 (PMC7540891; doi:10.1371/journal.pone.0239994)
Supplement: S3 Fig — Models use restricted analytic sample and classify dementia using the Langa-Weir classification scheme. Predictors with HRs equal to zero are excluded from the figure but retained in S5 Table in S1 File. (PDF) [file pone.0239994.s003.pdf]

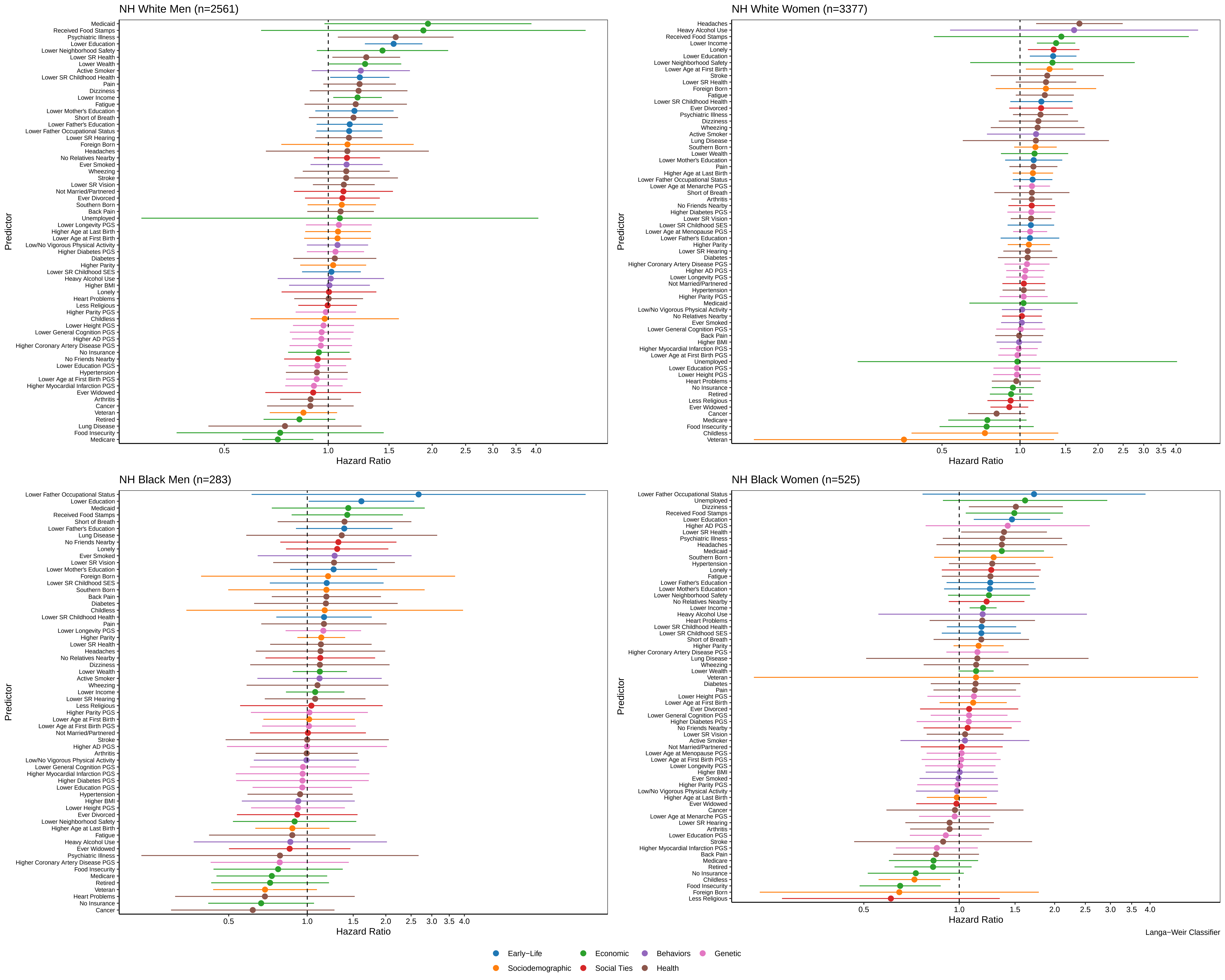

NH White Men (n=2561)
NH White Women (n=3377)
NH Black Men (n=283)
NH Black Women (n=525)
Predictor
Hazard Ratio
0.5
1.0
1.5
2.0
2.5
3.0
3.5
4.0
Langa−Weir Classifier
Early−Life
Sociodemographic
Economic
Social Ties
Behaviors
Health
Genetic
