# Supplementary material for: A data-driven prospective study of dementia among older adults in the United States
Source: PLoS One. 2020 Oct 7;15(10):e0239994. doi: 10.1371/journal.pone.0239994 (PMC7540891; doi:10.1371/journal.pone.0239994)
Supplement: S4 Fig — Models use restricted analytic sample and classify dementia using the Hurd classification scheme. Predictors with HRs equal to zero are excluded from the figure but retained in S6 Table in S1 File. (PDF) [file pone.0239994.s004.pdf]

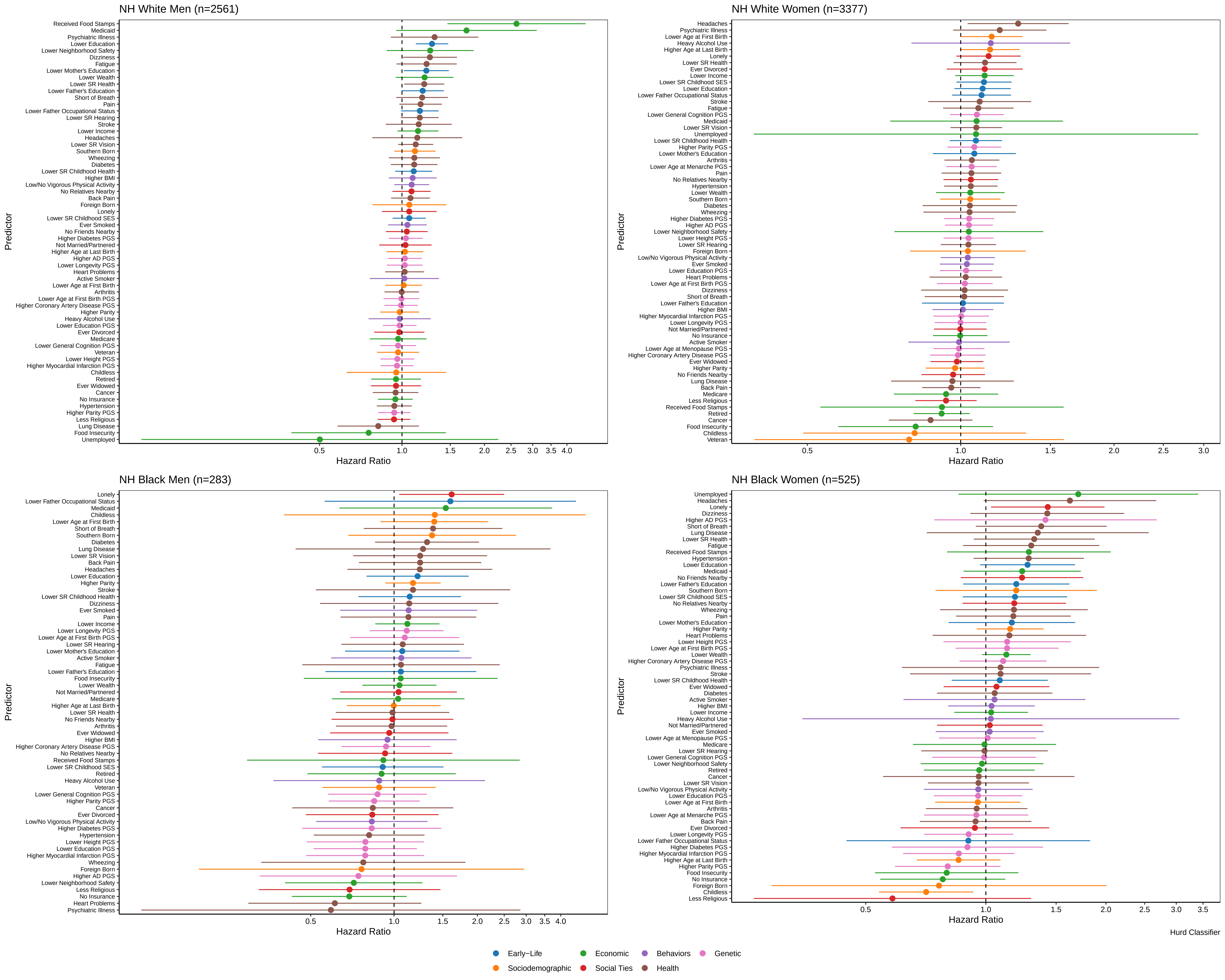
NH White Men (n=2561)
Received Food Stamps
Medicaid
Psychiatric Illness
Lower Education
Lower Neighborhood Safety
Dizziness
Fatigue
Lower Mother's Education
Lower Wealth
Lower SR Health
Lower Father's Education
Short of Breath
Pain
Lower Father Occupational Status
Lower SR Hearing
Stroke
Lower Income
Headaches
Lower SR Vision
Southern Born
Wheezing
Diabetes
Lower SR Childhood Health
Higher BMI
Low/No Vigorous Physical Activity
No Relatives Nearby
Back Pain
Foreign Born
Lonely
Lower SR Childhood SES
Ever Smoked
No Friends Nearby
Higher Diabetes PGS
Not Married/Partnered
Higher Age at Last Birth
Higher AD PGS
Lower Longevity PGS
Heart Problems
Active Smoker
Lower Age at First Birth
Arthritis
Lower Age at First Birth PGS
Higher Coronary Artery Disease PGS
Higher Parity
Heavy Alcohol Use
Lower Education PGS
Ever Divorced
Medicare
Lower General Cognition PGS
Veteran
Lower Height PGS
Higher Myocardial Infarction PGS
Childless
Retired
Ever Widowed
Cancer
No Insurance
Hypertension
Higher Parity PGS
Less Religious
Lung Disease
Food Insecurity
Unemployed
Predictor
Hazard Ratio
0.5 1.0 1.5 2.0 2.5 3.0 3.5 4.0
NH White Women (n=3377)
Headaches
Psychiatric Illness
Lower Age at First Birth
Heavy Alcohol Use
Higher Age at Last Birth
Lonely
Lower SR Health
Ever Divorced
Lower Income
Lower SR Childhood SES
Lower Education
Lower Father Occupational Status
Stroke
Fatigue
Lower General Cognition PGS
Medicaid
Lower SR Vision
Unemployed
Lower SR Childhood Health
Higher Parity PGS
Lower Mother's Education
Arthritis
Lower Age at Menarche PGS
Pain
No Relatives Nearby
Hypertension
Lower Wealth
Southern Born
Diabetes
Wheezing
Higher Diabetes PGS
Higher AD PGS
Lower Neighborhood Safety
Lower Height PGS
Lower SR Hearing
Foreign Born
Low/No Vigorous Physical Activity
Ever Smoked
Lower Education PGS
Heart Problems
Lower Age at First Birth PGS
Dizziness
Short of Breath
Lower Father's Education
Higher BMI
Higher Myocardial Infarction PGS
Lower Longevity PGS
Not Married/Partnered
No Insurance
Active Smoker
Lower Age at Menopause PGS
Higher Coronary Artery Disease PGS
Ever Widowed
Higher Parity
No Friends Nearby
Lung Disease
Back Pain
Medicare
Less Religious
Received Food Stamps
Retired
Cancer
Food Insecurity
Childless
Veteran
Predictor
Hazard Ratio
0.5 1.0 1.5 2.0 2.5 3.0
NH Black Men (n=283)
Lonely
Lower Father Occupational Status
Medicaid
Childless
Lower Age at First Birth
Short of Breath
Southern Born
Diabetes
Lung Disease
Lower SR Vision
Back Pain
Headaches
Lower Education
Higher Parity
Stroke
Lower SR Childhood Health
Dizziness
Ever Smoked
Pain
Lower Income
Lower Longevity PGS
Lower Age at First Birth PGS
Lower SR Hearing
Lower Mother's Education
Active Smoker
Fatigue
Lower Father's Education
Food Insecurity
Lower Wealth
Not Married/Partnered
Medicare
Higher Age at Last Birth
Lower SR Health
No Friends Nearby
Arthritis
Ever Widowed
Higher BMI
Higher Coronary Artery Disease PGS
No Relatives Nearby
Received Food Stamps
Lower SR Childhood SES
Retired
Heavy Alcohol Use
Veteran
Lower General Cognition PGS
Higher Parity PGS
Cancer
Ever Divorced
Low/No Vigorous Physical Activity
Higher Diabetes PGS
Hypertension
Lower Height PGS
Lower Education PGS
Higher Myocardial Infarction PGS
Wheezing
Foreign Born
Higher AD PGS
Lower Neighborhood Safety
Less Religious
No Insurance
Heart Problems
Psychiatric Illness
Predictor
Hazard Ratio
0.5 1.0 1.5 2.0 2.5 3.0 3.5 4.0
NH Black Women (n=525)
Unemployed
Headaches
Lonely
Dizziness
Higher AD PGS
Short of Breath
Lung Disease
Lower SR Health
Fatigue
Received Food Stamps
Hypertension
Lower Education
Medicaid
No Friends Nearby
Lower Father's Education
Southern Born
Lower SR Childhood SES
No Relatives Nearby
Wheezing
Pain
Lower Mother's Education
Higher Parity
Heart Problems
Lower Height PGS
Lower Age at First Birth PGS
Lower Wealth
Higher Coronary Artery Disease PGS
Psychiatric Illness
Stroke
Lower SR Childhood Health
Ever Widowed
Diabetes
Active Smoker
Higher BMI
Lower Income
Heavy Alcohol Use
Not Married/Partnered
Ever Smoked
Lower Age at Menopause PGS
Medicare
Lower SR Hearing
Lower General Cognition PGS
Lower Neighborhood Safety
Retired
Cancer
Lower SR Vision
Low/No Vigorous Physical Activity
Lower Education PGS
Lower Age at First Birth
Arthritis
Lower Age at Menarche PGS
Back Pain
Ever Divorced
Lower Longevity PGS
Lower Father Occupational Status
Higher Diabetes PGS
Higher Myocardial Infarction PGS
Higher Age at Last Birth
Higher Parity PGS
Food Insecurity
No Insurance
Foreign Born
Childless
Less Religious
Predictor
Hazard Ratio
0.5 1.0 1.5 2.0 2.5 3.0 3.5
Hurd Classifier
Early−Life
Economic
Behaviors
Genetic
Sociodemographic
Social Ties
Health
