# Supplementary material for: A data-driven prospective study of dementia among older adults in the United States
Source: PLoS One. 2020 Oct 7;15(10):e0239994. doi: 10.1371/journal.pone.0239994 (PMC7540891; doi:10.1371/journal.pone.0239994)
Supplement: S5 Fig — Models use restricted analytic sample and classify dementia using the Expert classification scheme. Predictors with HRs equal to zero are excluded from the figure but retained in S7 Table in S1 File. (PDF) [file pone.0239994.s005.pdf]

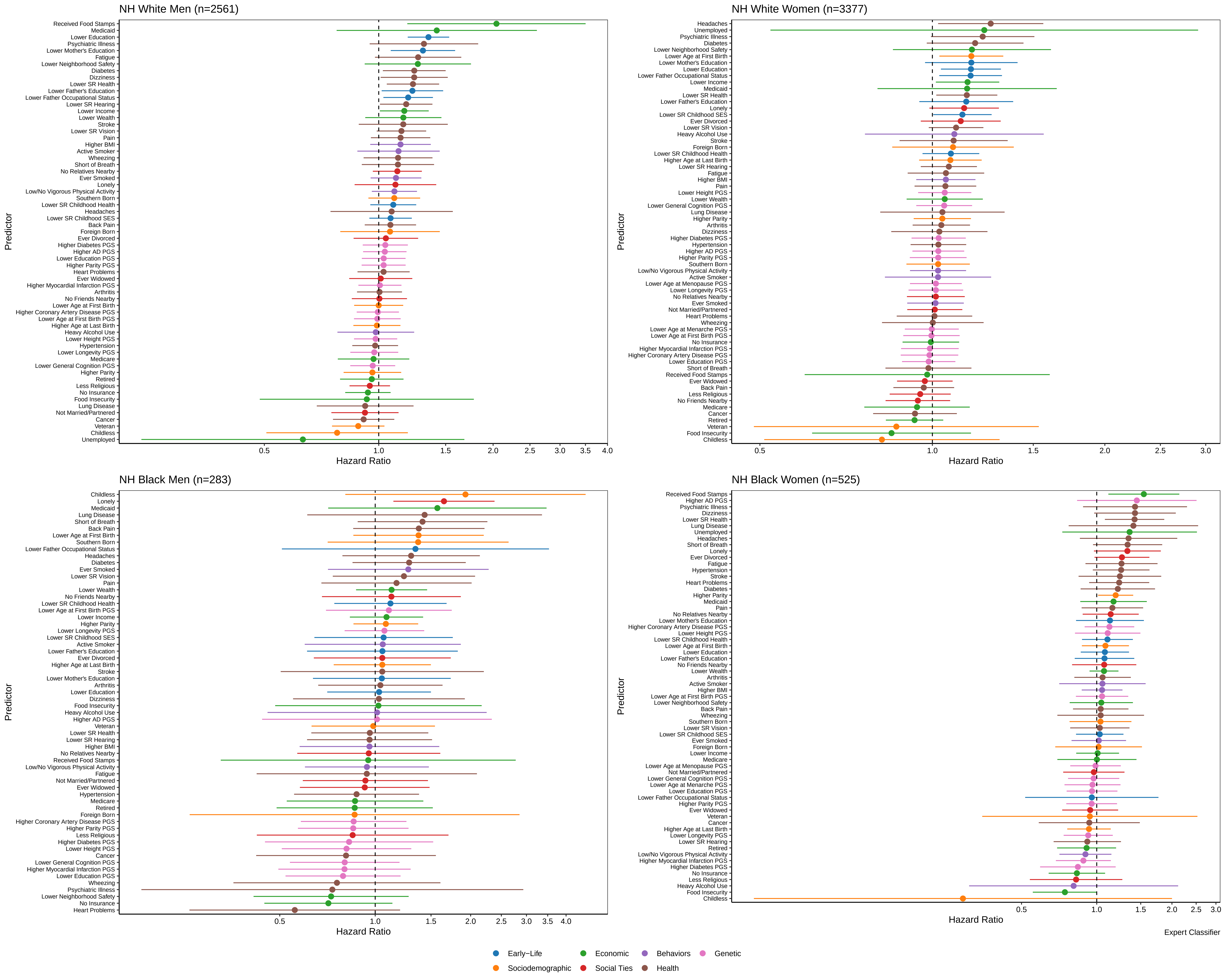
NH White Men (n=2561)
Received Food Stamps
Medicaid
Lower Education
Psychiatric Illness
Lower Mother's Education
Fatigue
Lower Neighborhood Safety
Diabetes
Dizziness
Lower SR Health
Lower Father's Education
Lower Father Occupational Status
Lower SR Hearing
Lower Income
Lower Wealth
Stroke
Lower SR Vision
Pain
Higher BMI
Active Smoker
Wheezing
Short of Breath
No Relatives Nearby
Ever Smoked
Lonely
Low/No Vigorous Physical Activity
Southern Born
Lower SR Childhood Health
Headaches
Lower SR Childhood SES
Back Pain
Foreign Born
Ever Divorced
Higher Diabetes PGS
Higher AD PGS
Lower Education PGS
Higher Parity PGS
Heart Problems
Ever Widowed
Higher Myocardial Infarction PGS
Arthritis
No Friends Nearby
Lower Age at First Birth
Higher Coronary Artery Disease PGS
Lower Age at First Birth PGS
Higher Age at Last Birth
Heavy Alcohol Use
Lower Height PGS
Hypertension
Lower Longevity PGS
Medicare
Lower General Cognition PGS
Higher Parity
Retired
Less Religious
No Insurance
Food Insecurity
Lung Disease
Not Married/Partnered
Cancer
Veteran
Childless
Unemployed
Predictor
Hazard Ratio
0.5
1.0
1.5
2.0
2.5
3.0
3.5
4.0
NH White Women (n=3377)
Headaches
Unemployed
Psychiatric Illness
Diabetes
Lower Neighborhood Safety
Lower Age at First Birth
Lower Mother's Education
Lower Education
Lower Father Occupational Status
Lower Income
Medicaid
Lower SR Health
Lower Father's Education
Lonely
Lower SR Childhood SES
Ever Divorced
Lower SR Vision
Heavy Alcohol Use
Stroke
Foreign Born
Lower SR Childhood Health
Higher Age at Last Birth
Lower SR Hearing
Fatigue
Higher BMI
Pain
Lower Height PGS
Lower Wealth
Lower General Cognition PGS
Lung Disease
Higher Parity
Arthritis
Dizziness
Higher Diabetes PGS
Hypertension
Higher AD PGS
Higher Parity PGS
Southern Born
Low/No Vigorous Physical Activity
Active Smoker
Lower Age at Menopause PGS
Lower Longevity PGS
No Relatives Nearby
Ever Smoked
Not Married/Partnered
Heart Problems
Wheezing
Lower Age at Menarche PGS
Lower Age at First Birth PGS
No Insurance
Higher Myocardial Infarction PGS
Higher Coronary Artery Disease PGS
Lower Education PGS
Short of Breath
Received Food Stamps
Ever Widowed
Back Pain
Less Religious
No Friends Nearby
Medicare
Cancer
Retired
Veteran
Food Insecurity
Childless
Predictor
Hazard Ratio
0.5
1.0
1.5
2.0
2.5
3.0
NH Black Men (n=283)
Childless
Lonely
Medicaid
Lung Disease
Short of Breath
Back Pain
Lower Age at First Birth
Southern Born
Lower Father Occupational Status
Headaches
Diabetes
Ever Smoked
Lower SR Vision
Pain
Lower Wealth
No Friends Nearby
Lower SR Childhood Health
Lower Age at First Birth PGS
Lower Income
Higher Parity
Lower Longevity PGS
Lower SR Childhood SES
Active Smoker
Lower Father's Education
Ever Divorced
Higher Age at Last Birth
Stroke
Lower Mother's Education
Arthritis
Lower Education
Dizziness
Food Insecurity
Heavy Alcohol Use
Higher AD PGS
Veteran
Lower SR Health
Lower SR Hearing
Higher BMI
No Relatives Nearby
Received Food Stamps
Low/No Vigorous Physical Activity
Fatigue
Not Married/Partnered
Ever Widowed
Hypertension
Medicare
Retired
Foreign Born
Higher Coronary Artery Disease PGS
Higher Parity PGS
Less Religious
Higher Diabetes PGS
Lower Height PGS
Cancer
Lower General Cognition PGS
Higher Myocardial Infarction PGS
Lower Education PGS
Wheezing
Psychiatric Illness
Lower Neighborhood Safety
No Insurance
Heart Problems
Predictor
Hazard Ratio
0.5
1.0
1.5
2.0
2.5
3.0
3.5
4.0
NH Black Women (n=525)
Received Food Stamps
Higher AD PGS
Psychiatric Illness
Dizziness
Lower SR Health
Lung Disease
Unemployed
Headaches
Short of Breath
Lonely
Ever Divorced
Fatigue
Hypertension
Stroke
Heart Problems
Diabetes
Higher Parity
Medicaid
Pain
No Relatives Nearby
Lower Mother's Education
Higher Coronary Artery Disease PGS
Lower Height PGS
Lower SR Childhood Health
Lower Age at First Birth
Lower Education
Lower Father's Education
No Friends Nearby
Lower Wealth
Arthritis
Active Smoker
Higher BMI
Lower Age at First Birth PGS
Lower Neighborhood Safety
Back Pain
Wheezing
Southern Born
Lower SR Vision
Lower SR Childhood SES
Ever Smoked
Foreign Born
Lower Income
Medicare
Lower Age at Menopause PGS
Not Married/Partnered
Lower General Cognition PGS
Lower Age at Menarche PGS
Lower Education PGS
Lower Father Occupational Status
Higher Parity PGS
Ever Widowed
Veteran
Cancer
Higher Age at Last Birth
Lower Longevity PGS
Lower SR Hearing
Retired
Low/No Vigorous Physical Activity
Higher Myocardial Infarction PGS
Higher Diabetes PGS
No Insurance
Less Religious
Heavy Alcohol Use
Food Insecurity
Childless
Predictor
Hazard Ratio
0.5
1.0
1.5
2.0
2.5
3.0
Expert Classifier
Early−Life
Economic
Behaviors
Genetic
Sociodemographic
Social Ties
Health
