# Supplementary material for: A data-driven prospective study of dementia among older adults in the United States
Source: PLoS One. 2020 Oct 7;15(10):e0239994. doi: 10.1371/journal.pone.0239994 (PMC7540891; doi:10.1371/journal.pone.0239994)
Supplement: S6 Fig — Models use restricted analytic sample and classify dementia using the LASSO classification scheme. Predictors with HRs equal to zero are excluded from the figure but retained in S8 Table in S1 File. (PDF) [file pone.0239994.s006.pdf]

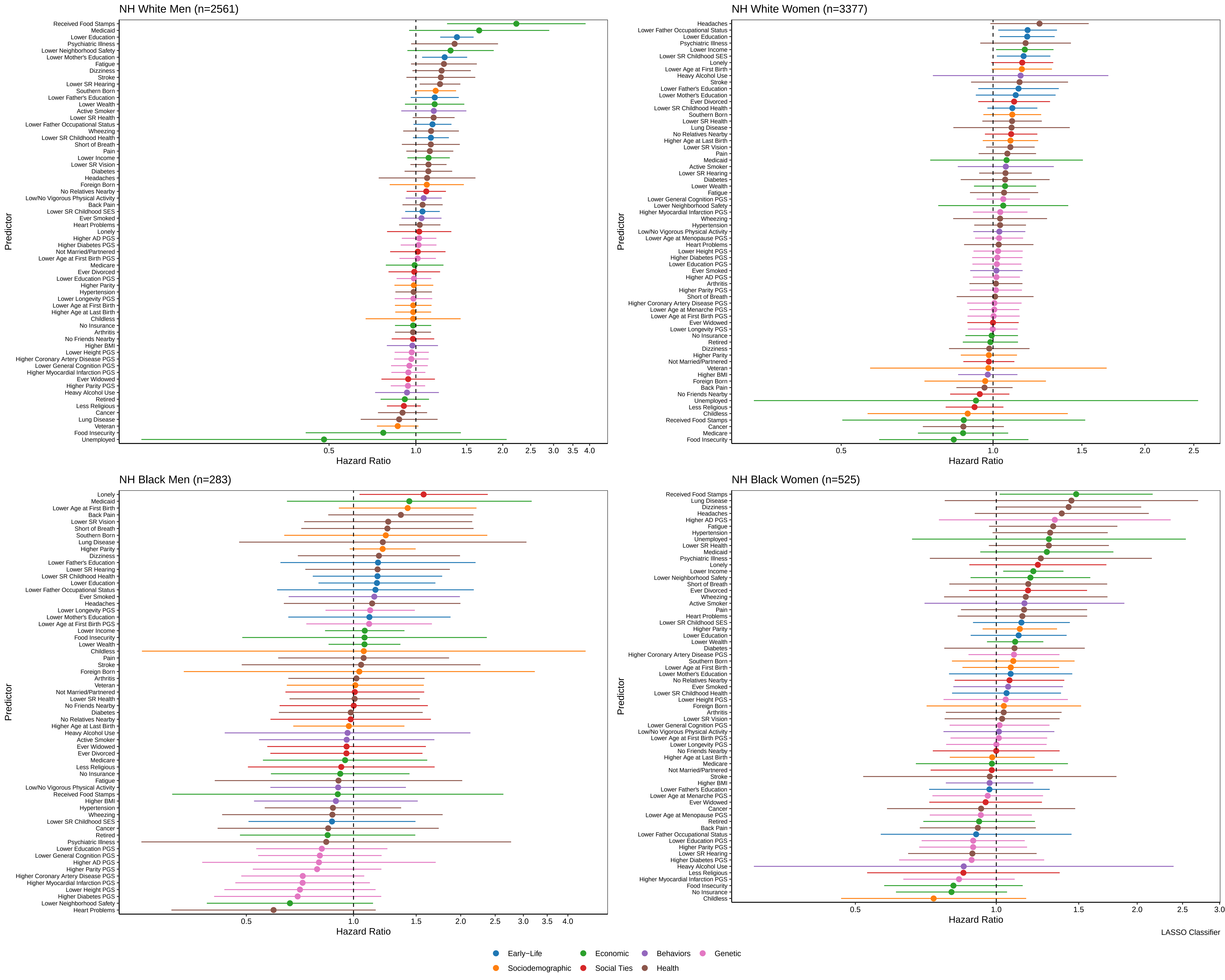
NH White Men (n=2561)
Received Food Stamps
Medicaid
Lower Education
Psychiatric Illness
Lower Neighborhood Safety
Lower Mother's Education
Fatigue
Dizziness
Stroke
Lower SR Hearing
Southern Born
Lower Father's Education
Lower Wealth
Active Smoker
Lower SR Health
Lower Father Occupational Status
Wheezing
Lower SR Childhood Health
Short of Breath
Pain
Lower Income
Lower SR Vision
Diabetes
Headaches
Foreign Born
No Relatives Nearby
Low/No Vigorous Physical Activity
Back Pain
Lower SR Childhood SES
Ever Smoked
Heart Problems
Lonely
Higher AD PGS
Higher Diabetes PGS
Not Married/Partnered
Lower Age at First Birth PGS
Medicare
Ever Divorced
Lower Education PGS
Higher Parity
Hypertension
Lower Longevity PGS
Lower Age at First Birth
Higher Age at Last Birth
Childless
No Insurance
Arthritis
No Friends Nearby
Higher BMI
Lower Height PGS
Higher Coronary Artery Disease PGS
Lower General Cognition PGS
Higher Myocardial Infarction PGS
Ever Widowed
Higher Parity PGS
Heavy Alcohol Use
Retired
Less Religious
Cancer
Lung Disease
Veteran
Food Insecurity
Unemployed
Predictor
0.5
1.0
1.5
2.0
2.5
3.0
3.5
4.0
Hazard Ratio
NH White Women (n=3377)
Headaches
Lower Father Occupational Status
Lower Education
Psychiatric Illness
Lower Income
Lower SR Childhood SES
Lonely
Lower Age at First Birth
Heavy Alcohol Use
Stroke
Lower Father's Education
Lower Mother's Education
Ever Divorced
Lower SR Childhood Health
Southern Born
Lower SR Health
Lung Disease
No Relatives Nearby
Higher Age at Last Birth
Lower SR Vision
Pain
Medicaid
Active Smoker
Lower SR Hearing
Diabetes
Lower Wealth
Fatigue
Lower General Cognition PGS
Lower Neighborhood Safety
Higher Myocardial Infarction PGS
Wheezing
Hypertension
Low/No Vigorous Physical Activity
Lower Age at Menopause PGS
Heart Problems
Lower Height PGS
Higher Diabetes PGS
Lower Education PGS
Ever Smoked
Higher AD PGS
Arthritis
Higher Parity PGS
Short of Breath
Higher Coronary Artery Disease PGS
Lower Age at Menarche PGS
Lower Age at First Birth PGS
Ever Widowed
Lower Longevity PGS
No Insurance
Retired
Dizziness
Higher Parity
Not Married/Partnered
Veteran
Higher BMI
Foreign Born
Back Pain
No Friends Nearby
Unemployed
Less Religious
Childless
Received Food Stamps
Cancer
Medicare
Food Insecurity
Predictor
0.5
1.0
1.5
2.0
2.5
Hazard Ratio
NH Black Men (n=283)
Lonely
Medicaid
Lower Age at First Birth
Back Pain
Lower SR Vision
Short of Breath
Southern Born
Lung Disease
Higher Parity
Dizziness
Lower Father's Education
Lower SR Hearing
Lower SR Childhood Health
Lower Education
Lower Father Occupational Status
Ever Smoked
Headaches
Lower Longevity PGS
Lower Mother's Education
Lower Age at First Birth PGS
Lower Income
Food Insecurity
Lower Wealth
Childless
Pain
Stroke
Foreign Born
Arthritis
Veteran
Not Married/Partnered
Lower SR Health
No Friends Nearby
Diabetes
No Relatives Nearby
Higher Age at Last Birth
Heavy Alcohol Use
Active Smoker
Ever Widowed
Ever Divorced
Medicare
Less Religious
No Insurance
Fatigue
Low/No Vigorous Physical Activity
Received Food Stamps
Higher BMI
Hypertension
Wheezing
Lower SR Childhood SES
Cancer
Retired
Psychiatric Illness
Lower Education PGS
Lower General Cognition PGS
Higher AD PGS
Higher Parity PGS
Higher Coronary Artery Disease PGS
Higher Myocardial Infarction PGS
Lower Height PGS
Higher Diabetes PGS
Lower Neighborhood Safety
Heart Problems
Predictor
0.5
1.0
1.5
2.0
2.5
3.0
3.5
4.0
Hazard Ratio
NH Black Women (n=525)
Received Food Stamps
Lung Disease
Dizziness
Headaches
Higher AD PGS
Fatigue
Hypertension
Unemployed
Lower SR Health
Medicaid
Psychiatric Illness
Lonely
Lower Income
Lower Neighborhood Safety
Short of Breath
Ever Divorced
Wheezing
Active Smoker
Pain
Heart Problems
Lower SR Childhood SES
Higher Parity
Lower Education
Lower Wealth
Diabetes
Higher Coronary Artery Disease PGS
Southern Born
Lower Age at First Birth
Lower Mother's Education
No Relatives Nearby
Ever Smoked
Lower SR Childhood Health
Lower Height PGS
Foreign Born
Arthritis
Lower SR Vision
Lower General Cognition PGS
Low/No Vigorous Physical Activity
Lower Age at First Birth PGS
Lower Longevity PGS
No Friends Nearby
Higher Age at Last Birth
Medicare
Not Married/Partnered
Stroke
Higher BMI
Lower Father's Education
Lower Age at Menarche PGS
Ever Widowed
Cancer
Lower Age at Menopause PGS
Retired
Back Pain
Lower Father Occupational Status
Lower Education PGS
Higher Parity PGS
Lower SR Hearing
Higher Diabetes PGS
Heavy Alcohol Use
Less Religious
Higher Myocardial Infarction PGS
Food Insecurity
No Insurance
Childless
Predictor
0.5
1.0
1.5
2.0
2.5
3.0
Hazard Ratio
LASSO Classifier
Early−Life
Economic
Behaviors
Genetic
Sociodemographic
Social Ties
Health
