# Supplementary material for: A data-driven prospective study of dementia among older adults in the United States
Source: PLoS One. 2020 Oct 7;15(10):e0239994. doi: 10.1371/journal.pone.0239994 (PMC7540891; doi:10.1371/journal.pone.0239994)
Supplement: S13 Fig — Model uses restricted analytic sample and classifies dementia using the Expert classification scheme. (PDF) [file pone.0239994.s013.pdf]

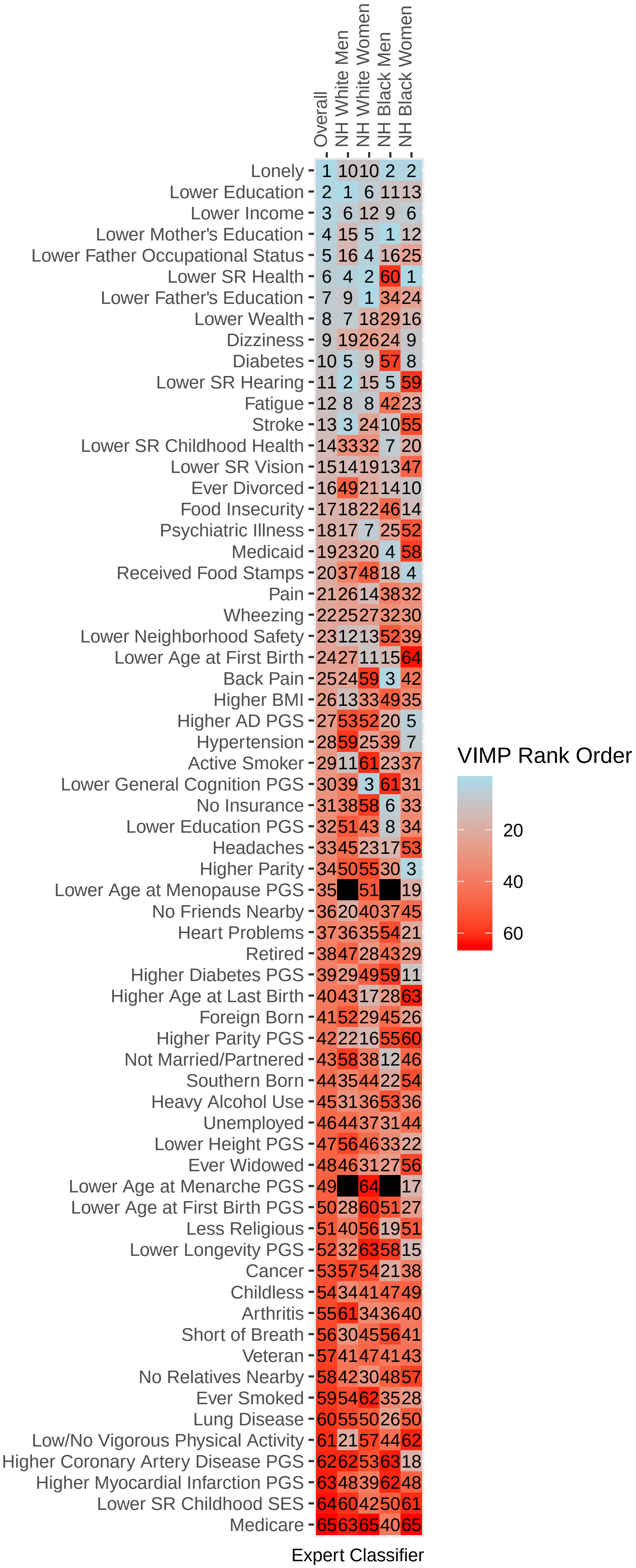

Overall
NH White Men
NH White Women
NH Black Men
NH Black Women
Lonely 1 10 10 2 2
Lower Education 2 1 6 11 13
Lower Income 3 6 12 9 6
Lower Mother's Education 4 15 5 1 12
Lower Father Occupational Status 5 16 4 16 25
Lower SR Health 6 4 2 60 1
Lower Father's Education 7 9 1 34 24
Lower Wealth 8 7 18 29 16
Dizziness 9 19 26 24 9
Diabetes 10 5 9 57 8
Lower SR Hearing 11 2 15 5 59
Fatigue 12 8 8 42 23
Stroke 13 3 24 10 55
Lower SR Childhood Health 14 33 32 7 20
Lower SR Vision 15 14 19 13 47
Ever Divorced 16 49 21 14 10
Food Insecurity 17 18 22 46 14
Psychiatric Illness 18 17 7 25 52
Medicaid 19 23 20 4 58
Received Food Stamps 20 37 48 18 4
Pain 21 26 14 38 32
Wheezing 22 25 27 32 30
Lower Neighborhood Safety 23 12 13 52 39
Lower Age at First Birth 24 27 11 15 64
Back Pain 25 24 59 3 42
Higher BMI 26 13 33 49 35
Higher AD PGS 27 53 52 20 5
Hypertension 28 59 25 39 7
Active Smoker 29 11 61 23 37
Lower General Cognition PGS 30 39 3 61 31
No Insurance 31 38 58 6 33
Lower Education PGS 32 51 43 8 34
Headaches 33 45 23 17 53
Higher Parity 34 50 55 30 3
Lower Age at Menopause PGS 35 51 19
No Friends Nearby 36 20 40 37 45
Heart Problems 37 36 35 54 21
Retired 38 47 28 43 29
Higher Diabetes PGS 39 29 49 59 11
Higher Age at Last Birth 40 43 17 28 63
Foreign Born 41 52 29 45 26
Higher Parity PGS 42 22 16 55 60
Not Married/Partnered 43 58 38 12 46
Southern Born 44 35 44 22 54
Heavy Alcohol Use 45 31 36 53 36
Unemployed 46 44 37 31 44
Lower Height PGS 47 56 46 33 22
Ever Widowed 48 46 31 27 56
Lower Age at Menarche PGS 49 64 17
Lower Age at First Birth PGS 50 28 60 51 27
Less Religious 51 40 56 19 51
Lower Longevity PGS 52 32 63 58 15
Cancer 53 57 54 21 38
Childless 54 34 41 47 49
Arthritis 55 61 34 36 40
Short of Breath 56 30 45 56 41
Veteran 57 41 47 41 43
No Relatives Nearby 58 42 30 48 57
Ever Smoked 59 54 62 35 28
Lung Disease 60 55 50 26 50
Low/No Vigorous Physical Activity 61 21 57 44 62
Higher Coronary Artery Disease PGS 62 62 53 63 18
Higher Myocardial Infarction PGS 63 48 39 62 48
Lower SR Childhood SES 64 60 42 50 61
Medicare 65 63 65 40 65
VIMP Rank Order
20
40
60
Expert Classifier
