# Supplementary material for: A data-driven prospective study of dementia among older adults in the United States
Source: PLoS One. 2020 Oct 7;15(10):e0239994. doi: 10.1371/journal.pone.0239994 (PMC7540891; doi:10.1371/journal.pone.0239994)
Supplement: S14 Fig — Model uses restricted analytic sample and classifies dementia using the LASSO classification scheme. (PDF) [file pone.0239994.s014.pdf]

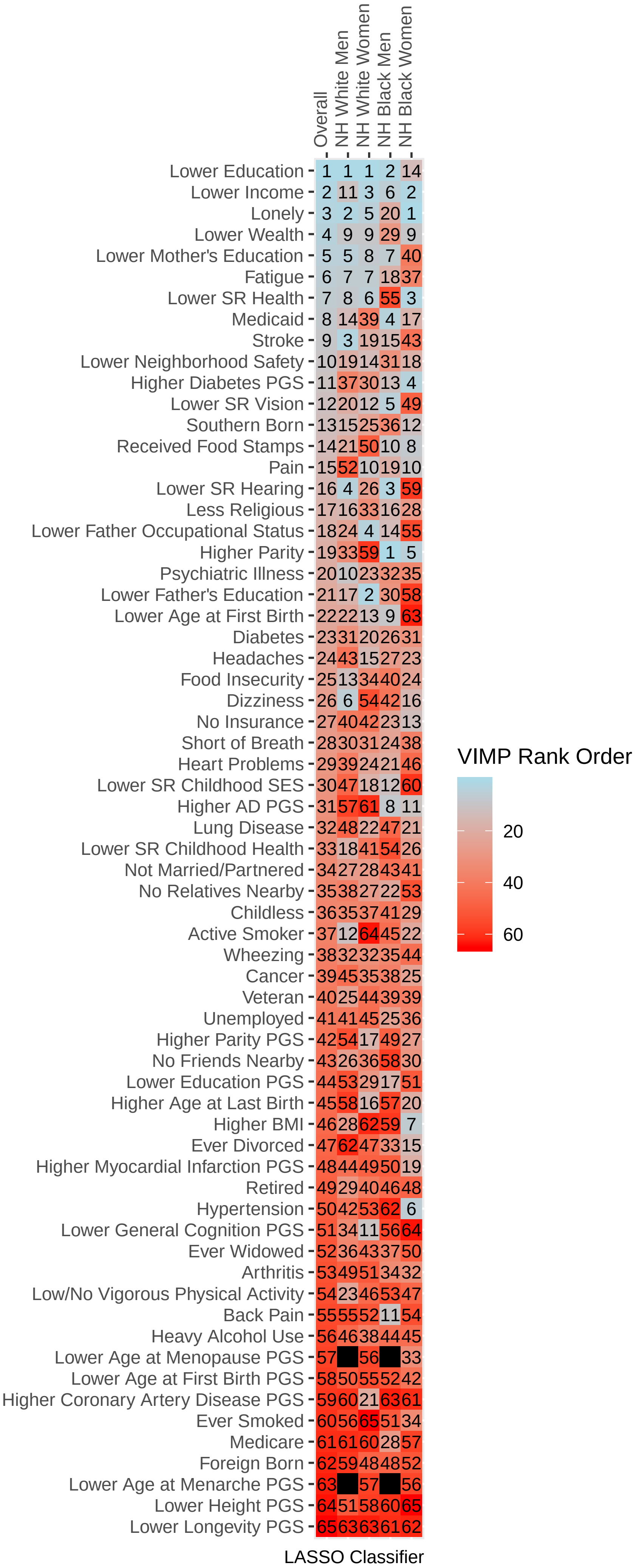

Overall
NH White Men
NH White Women
NH Black Men
NH Black Women
Lower Education 1 1 1 2 14
Lower Income 2 11 3 6 2
Lonely 3 2 5 20 1
Lower Wealth 4 9 9 29 9
Lower Mother's Education 5 5 8 7 40
Fatigue 6 7 7 18 37
Lower SR Health 7 8 6 55 3
Medicaid 8 14 39 4 17
Stroke 9 3 19 15 43
Lower Neighborhood Safety 10 19 14 31 18
Higher Diabetes PGS 11 37 30 13 4
Lower SR Vision 12 20 12 5 49
Southern Born 13 15 25 36 12
Received Food Stamps 14 21 50 10 8
Pain 15 52 10 19 10
Lower SR Hearing 16 4 26 3 59
Less Religious 17 16 33 16 28
Lower Father Occupational Status 18 24 4 14 55
Higher Parity 19 33 59 1 5
Psychiatric Illness 20 10 23 32 35
Lower Father's Education 21 17 2 30 58
Lower Age at First Birth 22 22 13 9 63
Diabetes 23 31 20 26 31
Headaches 24 43 15 27 23
Food Insecurity 25 13 34 40 24
Dizziness 26 6 54 42 16
No Insurance 27 40 42 23 13
Short of Breath 28 30 31 24 38
Heart Problems 29 39 24 21 46
Lower SR Childhood SES 30 47 18 12 60
Higher AD PGS 31 57 61 8 11
Lung Disease 32 48 22 47 21
Lower SR Childhood Health 33 18 41 54 26
Not Married/Partnered 34 27 28 43 41
No Relatives Nearby 35 38 27 22 53
Childless 36 35 37 41 29
Active Smoker 37 12 64 45 22
Wheezing 38 32 32 35 44
Cancer 39 45 35 38 25
Veteran 40 25 44 39 39
Unemployed 41 41 45 25 36
Higher Parity PGS 42 54 17 49 27
No Friends Nearby 43 26 36 58 30
Lower Education PGS 44 53 29 17 51
Higher Age at Last Birth 45 58 16 57 20
Higher BMI 46 28 62 59 7
Ever Divorced 47 62 47 33 15
Higher Myocardial Infarction PGS 48 44 49 50 19
Retired 49 29 40 46 48
Hypertension 50 42 53 62 6
Lower General Cognition PGS 51 34 11 56 64
Ever Widowed 52 36 43 37 50
Arthritis 53 49 51 34 32
Low/No Vigorous Physical Activity 54 23 46 53 47
Back Pain 55 55 52 11 54
Heavy Alcohol Use 56 46 38 44 45
Lower Age at Menopause PGS 57 56 33
Lower Age at First Birth PGS 58 50 55 52 42
Higher Coronary Artery Disease PGS 59 60 21 63 61
Ever Smoked 60 56 65 51 34
Medicare 61 61 60 28 57
Foreign Born 62 59 48 48 52
Lower Age at Menarche PGS 63 57 56
Lower Height PGS 64 51 58 60 65
Lower Longevity PGS 65 63 63 61 62
LASSO Classifier
VIMP Rank Order
20
40
60
