# Supplementary material for: A data-driven prospective study of dementia among older adults in the United States
Source: PLoS One. 2020 Oct 7;15(10):e0239994. doi: 10.1371/journal.pone.0239994 (PMC7540891; doi:10.1371/journal.pone.0239994)
Supplement: S1 File — (DOCX) [file pone.0239994.s015.docx]

**S1 Table. Descriptive characteristics of the full analytic sample, HRS 2000.**

| Characteristic | Statistic^*^ | % Missing |
| --- | --- | --- |
| *Sociodemographic* |  |  |
| Baseline Age | 65.59 (0.15) | 0.000 |
| Female, % | 0.56 (0.01) | 0.000 |
| Non-Hispanic Black, % | 0.10 (0.00) | 0.000 |
| Foreign Born, % | 0.08 (0.01) | 0.001 |
| Southern Born, % | 0.31 (0.01) | 0.001 |
| Veteran, % | 0.28 (0.01) | 0.001 |
| Childless, % | 0.03 (0.00) | 0.001 |
| Parity | 2.94 (0.02) | 0.001 |
| Age at First Birth | 24.90 (0.08) | 0.000 |
| Age at Last Birth | 31.98 (0.10) | 0.000 |
| *Early-Life* |  |  |
| Mother's Education, years | 9.64 (0.05) | 0.08 |
| Father's Education, years | 9.26 (0.05) | 0.123 |
| Father's Occupational Status, unit | 2.15 (0.03) | 0.132 |
| SR Childhood Health | 4.27 (0.01) | 0.013 |
| SR Childhood SES | 1.80 (0.01) | 0.013 |
| Education, years | 3.32 (0.02) | 0.000 |
| *Economic* |  |  |
| Food Insecurity, % | 0.04 (0.00) | 0.005 |
| Income, dollars | 66270.17 (2039.69) | 0.000 |
| Neighborhood Safety | 1.91 (0.01) | 0.006 |
| Wealth, dollars | 371402.5 (13790.59) | 0.000 |
| Medicaid, % | 0.04 (0.00) | 0.001 |
| Medicare, % | 0.45 (0.01) | 0.001 |
| No Insurance, % | 0.37 (0.01) | 0.021 |
| Received Food Stamps, % | 0.03 (0.00) | 0.006 |
| Retired, % | 0.52 (0.01) | 0.000 |
| Unemployed, % | 0.01 (0.00) | 0.000 |
| *Health* |  |  |
| Arthritis, % | 0.49 (0.01) | 0.001 |
| Back Pain, % | 0.31 (0.01) | 0.000 |
| Cancer, % | 0.11 (0.00) | 0.001 |
| Diabetes, % | 0.11 (0.00) | 0.000 |
| Dizziness, % | 0.09 (0.00) | 0.000 |
| Fatigue, % | 0.14 (0.01) | 0.000 |
| Headaches, % | 0.08 (0.00) | 0.000 |
| Heart Problems, % | 0.17 (0.01) | 0.000 |
| Hypertension, % | 0.42 (0.01) | 0.001 |
| SR Health | 3.53 (0.02) | 0.000 |
| SR Hearing | 4.47 (0.02) | 0.001 |
| SR Vision | 4.36 (0.01) | 0.000 |
| Lung Disease, % | 0.05 (0.00) | 0.000 |
| Pain, % | 0.26 (0.01) | 0.000 |
| Psychiatric Illness, % | 0.08 (0.00) | 0.000 |
| Short of Breath, % | 0.13 (0.00) | 0.000 |
| Stroke, % | 0.05 (0.00) | 0.000 |
| Wheezing, % | 0.10 (0.00) | 0.000 |
| Cataracts [Excluded] |  | 0.545 |
| Falls [Excluded] |  | 0.522 |
| Glaucoma [Excluded] |  | 0.541 |
| Swelling [Excluded] |  | 0.448 |
| *Behaviors* |  |  |
| Active Smoker, % | 0.14 (0.01) | 0.000 |
| Ever Smoked, % | 0.59 (0.01) | 0.000 |
| Heavy Alcohol Use, % | 0.06 (0.00) | 0.001 |
| BMI | 27.27 (0.07) | 0.01 |
| Low/No Vigorous Physical Activity, % | 0.5 (0.01) | 0.000 |
| *Social Connections* |  |  |
| Ever Divorced, % | 0.29 (0.01) | 0.000 |
| Ever Widowed, % | 0.23 (0.01) | 0.000 |
| Religious | 2.52 (0.01) | 0.001 |
| Lonely, % | 0.15 (0.01) | 0.038 |
| No Friends Nearby, % | 0.69 (0.01) | 0.005 |
| No Relatives Nearby, % | 0.30 (0.01) | 0.006 |
| Not Married/Partnered, % | 0.33 (0.01) | 0.001 |
| *Genetic* |  |  |
| AD PGS | 0.00 (0.01) | 0.000 |
| Coronary Artery Disease PGS | -0.01 (0.01) | 0.000 |
| Diabetes PGS | 0.01 (0.01) | 0.000 |
| Myocardial Infarction PGS | 0.01 (0.01) | 0.000 |
| Parity PGS | -0.01 (0.01) | 0.000 |
| Age at First Birth PGS | 0.00 (0.01) | 0.000 |
| Age at Menarche PGS | 0.00 (0.01) | 0.000 |
| Age at Menopause PGS | 0.00 (0.01) | 0.000 |
| Education PGS | 0.00 (0.01) | 0.000 |
| General Cognition PGS | 0.00 (0.01) | 0.000 |
| Height PGS | 0.00 (0.01) | 0.000 |
| Longevity PGS | 0.00 (0.01) | 0.000 |

^*^ Weighted means or proportions with standard errors listed in parentheses.

**S2 Table. Subdistribution hazard ratios (sdHRs) and 95% confidence intervals (CI) of each predictor for incident dementia obtained from Fine and Gray regression models stratified by race and gender.**Models use full analytic sample and classify dementia using the Langa-Weir classification scheme.

|  | NH White | | NH Black | |
| --- | --- | --- | --- | --- |
| Characteristic | Men | Women | Men | Women |
| *Sociodemographic* |  |  |  |  |
| Foreign Born | 1.13 (0.73, 1.74) | 1.28 (0.83, 1.98) | 1.07 (0.37, 3.13) | 0.70 (0.28, 1.76) |
| Southern Born | 1.11 (0.89, 1.38) | 1.15 (0.95, 1.38) | 1.30 (0.57, 2.97) | 1.22 (0.81, 1.83) |
| Veteran | 0.86 (0.69, 1.07) | 0.35 (0.09, 1.33) | 0.65 (0.43, 0.99) | 1.05 (0.21, 5.13) |
| Childless | 0.95 (0.58, 1.56) | 0.72 (0.38, 1.38) | 1.10 (0.34, 3.58) | 0.73 (0.57, 0.95) |
| Higher Parity | 1.04 (0.84, 1.28) | 1.09 (0.90, 1.31) | 1.17 (0.97, 1.40) | 1.15 (0.97, 1.37) |
| Lower Age at First Birth | 1.06 (0.87, 1.30) | 1.31 (1.07, 1.61) | 1.03 (0.70, 1.52) | 1.12 (0.89, 1.42) |
| Higher Age at Last Birth | 1.07 (0.87, 1.32) | 1.11 (0.93, 1.33) | 0.88 (0.65, 1.19) | 0.98 (0.79, 1.23) |
| *Early-Life* |  |  |  |  |
| Lower Mother's Education | 1.21 (0.95, 1.54) | 1.16 (0.91, 1.49) | 1.20 (0.84, 1.70) | 1.27 (0.91, 1.76) |
| Lower Father's Education | 1.15 (0.94, 1.42) | 1.11 (0.86, 1.43) | 1.33 (0.87, 2.04) | 1.30 (0.96, 1.75) |
| Lower Father's Occupational Status | 1.18 (0.95, 1.46) | 1.11 (0.93, 1.32) | 2.20 (0.75, 6.42) | 1.70 (0.81, 3.56) |
| Lower SR Childhood Health | 1.24 (1.02, 1.50) | 1.20 (0.92, 1.58) | 1.17 (0.80, 1.72) | 1.20 (0.94, 1.52) |
| Lower SR Childhood SES | 1.02 (0.84, 1.23) | 1.12 (0.92, 1.37) | 1.15 (0.73, 1.81) | 1.20 (0.92, 1.58) |
| Lower Education | 1.56 (1.29, 1.89) | 1.35 (1.10, 1.65) | 1.59 (1.03, 2.47) | 1.47 (1.12, 1.92) |
| *Economic* |  |  |  |  |
| Food Insecurity | 0.75 (0.40, 1.43) | 0.78 (0.52, 1.17) | 0.74 (0.44, 1.26) | 0.68 (0.52, 0.91) |
| Lower Income | 1.18 (1.02, 1.37) | 1.36 (1.14, 1.62) | 1.13 (0.90, 1.42) | 1.17 (1.07, 1.28) |
| Lower Neighborhood Safety | 1.44 (0.95, 2.17) | 1.37 (0.69, 2.71) | 0.94 (0.57, 1.55) | 1.22 (0.91, 1.63) |
| Lower Wealth | 1.21 (0.98, 1.51) | 1.14 (0.85, 1.51) | 1.14 (0.91, 1.42) | 1.15 (1.02, 1.30) |
| Medicaid | 2.04 (1.09, 3.81) | 1.07 (0.67, 1.70) | 1.57 (0.84, 2.95) | 1.36 (1.01, 1.81) |
| Medicare | 0.77 (0.63, 0.95) | 0.82 (0.62, 1.08) | 0.95 (0.59, 1.52) | 0.89 (0.67, 1.18) |
| No Insurance | 0.93 (0.76, 1.14) | 0.93 (0.77, 1.12) | 0.58 (0.38, 0.89) | 0.71 (0.51, 0.99) |
| Received Food Stamps | 1.47 (0.58, 3.75) | 1.41 (0.46, 4.34) | 1.60 (0.93, 2.76) | 1.39 (1.00, 1.94) |
| Retired | 0.84 (0.68, 1.04) | 0.94 (0.77, 1.13) | 0.76 (0.49, 1.18) | 0.82 (0.63, 1.07) |
| Unemployed | 0.99 (0.27, 3.55) | 0.94 (0.23, 3.79) | 0.00 (0.00, 0.00) | 1.25 (0.53, 2.94) |
| *Health* |  |  |  |  |
| Arthritis | 0.92 (0.75, 1.12) | 1.11 (0.93, 1.32) | 1.06 (0.70, 1.62) | 0.98 (0.74, 1.28) |
| Back Pain | 1.07 (0.87, 1.33) | 0.99 (0.80, 1.22) | 1.33 (0.86, 2.06) | 0.90 (0.67, 1.22) |
| Cancer | 0.90 (0.67, 1.19) | 0.82 (0.64, 1.05) | 0.71 (0.33, 1.49) | 0.91 (0.56, 1.48) |
| Diabetes | 1.06 (0.81, 1.39) | 1.05 (0.81, 1.36) | 1.02 (0.56, 1.87) | 1.06 (0.77, 1.44) |
| Dizziness | 1.21 (0.88, 1.66) | 1.17 (0.83, 1.66) | 1.20 (0.68, 2.11) | 1.51 (1.09, 2.08) |
| Fatigue | 1.15 (0.83, 1.60) | 1.24 (0.96, 1.59) | 0.86 (0.44, 1.67) | 1.24 (0.88, 1.73) |
| Headaches | 1.01 (0.59, 1.72) | 1.65 (1.14, 2.39) | 1.22 (0.71, 2.12) | 1.35 (0.88, 2.09) |
| Heart Problems | 1.03 (0.82, 1.28) | 0.97 (0.78, 1.20) | 0.78 (0.39, 1.55) | 1.17 (0.82, 1.67) |
| Hypertension | 0.95 (0.77, 1.16) | 1.04 (0.86, 1.25) | 1.00 (0.66, 1.52) | 1.30 (0.96, 1.77) |
| Lower SR Health | 1.24 (1.00, 1.54) | 1.24 (0.96, 1.61) | 1.29 (0.83, 2.01) | 1.35 (1.01, 1.79) |
| Lower SR Hearing | 1.17 (0.94, 1.46) | 1.08 (0.87, 1.33) | 1.01 (0.65, 1.58) | 0.98 (0.72, 1.33) |
| Lower SR Vision | 1.13 (0.93, 1.38) | 1.10 (0.92, 1.32) | 1.37 (0.81, 2.29) | 1.09 (0.82, 1.43) |
| Lung Disease | 0.71 (0.43, 1.18) | 1.14 (0.61, 2.12) | 1.22 (0.56, 2.66) | 0.97 (0.44, 2.13) |
| Pain | 1.22 (0.96, 1.54) | 1.12 (0.91, 1.38) | 1.38 (0.85, 2.24) | 1.18 (0.89, 1.57) |
| Psychiatric Illness | 1.61 (1.13, 2.31) | 1.21 (0.95, 1.54) | 1.12 (0.41, 3.09) | 1.19 (0.77, 1.84) |
| Short of Breath | 1.15 (0.86, 1.53) | 1.11 (0.80, 1.54) | 1.48 (0.86, 2.55) | 1.26 (0.92, 1.74) |
| Stroke | 1.13 (0.81, 1.59) | 1.28 (0.78, 2.10) | 1.08 (0.53, 2.19) | 0.99 (0.54, 1.85) |
| Wheezing | 1.10 (0.83, 1.46) | 1.17 (0.78, 1.75) | 1.22 (0.69, 2.16) | 1.15 (0.81, 1.65) |
| *Behaviors* |  |  |  |  |
| Active Smoker | 1.23 (0.91, 1.67) | 1.15 (0.71, 1.86) | 1.14 (0.77, 1.69) | 1.01 (0.66, 1.55) |
| Ever Smoked | 1.10 (0.87, 1.39) | 1.27 (0.68, 2.38) | 1.02 (0.85, 1.22) | 0.98 (0.75, 1.29) |
| Heavy Alcohol Use | 0.98 (0.69, 1.39) | 0.93 (0.45, 1.89) | 1.57 (0.53, 4.69) | 1.44 (0.65, 3.19) |
| Higher BMI | 1.01 (0.78, 1.31) | 0.89 (0.60, 1.33) | 0.97 (0.80, 1.18) | 1.04 (0.81, 1.33) |
| Low/No Vigorous Physical Activity | 1.05 (0.86, 1.29) | 1.05 (0.68, 1.61) | 1.01 (0.85, 1.21) | 1.00 (0.76, 1.33) |
| *Social Connections* |  |  |  |  |
| Ever Divorced | 1.08 (0.85, 1.37) | 1.17 (0.89, 1.54) | 0.95 (0.59, 1.53) | 1.04 (0.75, 1.44) |
| Ever Widowed | 0.91 (0.66, 1.24) | 0.93 (0.79, 1.10) | 0.90 (0.53, 1.53) | 1.02 (0.77, 1.34) |
| Less Religious | 0.98 (0.81, 1.18) | 0.92 (0.75, 1.12) | 0.98 (0.54, 1.76) | 0.57 (0.26, 1.25) |
| Lonely | 1.05 (0.77, 1.42) | 1.35 (1.08, 1.69) | 1.26 (0.81, 1.95) | 1.21 (0.87, 1.69) |
| No Friends Nearby | 0.93 (0.75, 1.16) | 1.12 (0.91, 1.37) | 1.16 (0.72, 1.87) | 1.10 (0.81, 1.49) |
| No Relatives Nearby | 1.14 (0.92, 1.41) | 1.02 (0.86, 1.21) | 1.17 (0.74, 1.83) | 1.20 (0.92, 1.57) |
| Not Married/Partnered | 1.10 (0.80, 1.51) | 1.02 (0.85, 1.24) | 0.96 (0.60, 1.52) | 0.96 (0.72, 1.27) |
| *Genetic* |  |  |  |  |
| Higher AD PGS | 0.95 (0.78, 1.15) | 1.04 (0.88, 1.23) | 1.08 (0.56, 2.06) | 1.29 (0.74, 2.26) |
| Higher Coronary Artery Disease PGS | 0.97 (0.79, 1.19) | 1.06 (0.87, 1.29) | 0.81 (0.49, 1.34) | 1.11 (0.90, 1.37) |
| Higher Diabetes PGS | 1.04 (0.87, 1.24) | 1.09 (0.89, 1.34) | 0.94 (0.54, 1.65) | 1.07 (0.74, 1.55) |
| Higher Myocardial Infarction PGS | 0.90 (0.75, 1.09) | 0.98 (0.83, 1.16) | 1.01 (0.60, 1.72) | 0.88 (0.66, 1.17) |
| Higher Parity PGS | 0.95 (0.78, 1.16) | 1.01 (0.82, 1.25) | 0.98 (0.63, 1.55) | 0.99 (0.75, 1.30) |
| Lower Age at First Birth PGS | 0.94 (0.76, 1.15) | 0.97 (0.82, 1.15) | 1.01 (0.70, 1.45) | 0.99 (0.76, 1.29) |
| Lower Age at Menarche PGS | — | 1.11 (0.95, 1.29) | — | 0.98 (0.76, 1.25) |
| Lower Age at Menopause PGS | — | 1.09 (0.94, 1.27) | — | 1.02 (0.80, 1.30) |
| Lower Education PGS | 0.93 (0.77, 1.12) | 0.96 (0.79, 1.18) | 0.92 (0.62, 1.37) | 0.91 (0.69, 1.18) |
| Lower General Cognition PGS | 0.96 (0.78, 1.18) | 1.00 (0.81, 1.24) | 0.93 (0.60, 1.42) | 1.01 (0.78, 1.30) |
| Lower Height PGS | 0.95 (0.78, 1.16) | 0.96 (0.78, 1.18) | 0.93 (0.64, 1.34) | 1.15 (0.83, 1.59) |
| Lower Longevity PGS | 1.08 (0.87, 1.34) | 1.03 (0.88, 1.22) | 1.12 (0.81, 1.55) | 0.99 (0.77, 1.27) |

**S3 Table. Cause-specific hazard ratios (HRs) and 95% confidence intervals (CI) of each predictor for incident dementia obtained from independent cause-specific hazard regression models stratified by race and gender.** Models use full analytic sample and classify dementia using the Langa-Weir classification scheme.

|  | NH White | | NH Black | |
| --- | --- | --- | --- | --- |
| Characteristic | Men | Women | Men | Women |
| *Sociodemographic* |  |  |  |  |
| Foreign Born | 1.07 (0.68, 1.67) | 1.23 (0.79, 1.91) | 1.12 (0.40, 3.19) | 0.69 (0.26, 1.83) |
| Southern Born | 1.22 (0.97, 1.53) | 1.21 (0.99, 1.47) | 1.27 (0.56, 2.86) | 1.23 (0.80, 1.87) |
| Veteran | 0.87 (0.69, 1.09) | 0.36 (0.09, 1.38) | 0.58 (0.36, 0.92) | 1.03 (0.21, 5.07) |
| Childless | 0.96 (0.60, 1.55) | 0.80 (0.43, 1.50) | 1.07 (0.33, 3.49) | 0.29 (0.04, 2.17) |
| Higher Parity | 1.07 (0.87, 1.33) | 1.12 (0.93, 1.35) | 1.14 (0.96, 1.37) | 1.17 (0.98, 1.39) |
| Lower Age at First Birth | 1.20 (0.96, 1.51) | 1.46 (1.18, 1.81) | 1.00 (0.67, 1.50) | 1.20 (0.95, 1.52) |
| Higher Age at Last Birth | 1.17 (0.93, 1.47) | 1.16 (0.97, 1.39) | 0.92 (0.67, 1.25) | 1.01 (0.80, 1.27) |
| *Early-Life* |  |  |  |  |
| Lower Mother's Education | 1.13 (0.86, 1.48) | 1.08 (0.80, 1.45) | 1.17 (0.81, 1.69) | 1.27 (0.90, 1.80) |
| Lower Father's Education | 1.12 (0.88, 1.42) | 1.13 (0.83, 1.52) | 1.27 (0.82, 1.98) | 1.28 (0.94, 1.74) |
| Lower Father's Occupational Status | 1.23 (0.98, 1.53) | 1.13 (0.95, 1.35) | 2.11 (0.78, 5.71) | 1.78 (0.80, 3.96) |
| Lower SR Childhood Health | 1.22 (1.00, 1.48) | 1.18 (0.89, 1.57) | 1.17 (0.80, 1.70) | 1.20 (0.94, 1.53) |
| Lower SR Childhood SES | 1.01 (0.83, 1.23) | 1.16 (0.94, 1.43) | 1.09 (0.69, 1.72) | 1.14 (0.85, 1.53) |
| Lower Education | 1.62 (1.34, 1.97) | 1.32 (1.07, 1.64) | 1.58 (1.02, 2.44) | 1.46 (1.11, 1.92) |
| *Economic* |  |  |  |  |
| Food Insecurity | 0.66 (0.34, 1.27) | 0.75 (0.49, 1.16) | 0.71 (0.44, 1.17) | 0.67 (0.50, 0.91) |
| Lower Income | 1.24 (1.06, 1.44) | 1.35 (1.13, 1.62) | 1.13 (0.90, 1.42) | 1.17 (1.07, 1.28) |
| Lower Neighborhood Safety | 1.47 (0.95, 2.27) | 1.45 (0.73, 2.90) | 0.95 (0.59, 1.56) | 1.26 (0.93, 1.70) |
| Lower Wealth | 1.31 (1.05, 1.65) | 1.18 (0.89, 1.58) | 1.11 (0.87, 1.42) | 1.16 (1.02, 1.32) |
| Medicaid | 2.31 (1.20, 4.47) | 1.14 (0.71, 1.84) | 1.63 (0.86, 3.10) | 1.44 (1.05, 1.97) |
| Medicare | 0.55 (0.42, 0.71) | 0.57 (0.42, 0.78) | 0.81 (0.48, 1.36) | 0.69 (0.51, 0.92) |
| No Insurance | 0.96 (0.78, 1.18) | 0.97 (0.80, 1.17) | 0.61 (0.40, 0.94) | 0.73 (0.52, 1.03) |
| Received Food Stamps | 1.80 (0.60, 5.38) | 1.73 (0.57, 5.23) | 1.63 (0.98, 2.72) | 1.63 (1.22, 2.18) |
| Retired | 0.70 (0.56, 0.89) | 0.93 (0.76, 1.14) | 0.62 (0.38, 1.01) | 0.75 (0.57, 1.00) |
| Unemployed | 1.03 (0.28, 3.81) | 1.20 (0.31, 4.70) | 0.00 (0.00, 0.00) | 1.44 (0.77, 2.69) |
| *Health* |  |  |  |  |
| Arthritis | 0.93 (0.76, 1.13) | 1.09 (0.91, 1.31) | 1.02 (0.66, 1.57) | 1.00 (0.75, 1.33) |
| Back Pain | 1.09 (0.88, 1.36) | 1.02 (0.82, 1.26) | 1.37 (0.88, 2.12) | 0.93 (0.69, 1.26) |
| Cancer | 0.87 (0.66, 1.17) | 0.79 (0.60, 1.04) | 0.75 (0.36, 1.55) | 1.01 (0.66, 1.54) |
| Diabetes | 1.10 (0.83, 1.46) | 1.20 (0.92, 1.56) | 1.09 (0.60, 1.96) | 1.17 (0.86, 1.60) |
| Dizziness | 1.16 (0.83, 1.63) | 1.19 (0.84, 1.70) | 1.15 (0.62, 2.14) | 1.55 (1.11, 2.16) |
| Fatigue | 1.23 (0.87, 1.75) | 1.29 (1.00, 1.67) | 0.93 (0.50, 1.72) | 1.35 (0.96, 1.89) |
| Headaches | 1.14 (0.67, 1.97) | 1.76 (1.20, 2.58) | 1.22 (0.69, 2.15) | 1.56 (1.05, 2.31) |
| Heart Problems | 1.01 (0.80, 1.27) | 1.00 (0.80, 1.25) | 0.81 (0.41, 1.60) | 1.22 (0.83, 1.78) |
| Hypertension | 0.95 (0.77, 1.16) | 1.04 (0.86, 1.26) | 1.02 (0.67, 1.57) | 1.31 (0.96, 1.78) |
| Lower SR Health | 1.32 (1.05, 1.65) | 1.33 (1.02, 1.74) | 1.31 (0.84, 2.04) | 1.48 (1.10, 2.00) |
| Lower SR Hearing | 1.10 (0.88, 1.38) | 1.03 (0.82, 1.28) | 1.04 (0.67, 1.62) | 0.97 (0.71, 1.34) |
| Lower SR Vision | 1.11 (0.91, 1.36) | 1.08 (0.90, 1.30) | 1.51 (0.90, 2.51) | 1.06 (0.80, 1.41) |
| Lung Disease | 0.83 (0.50, 1.37) | 1.34 (0.72, 2.47) | 1.23 (0.52, 2.90) | 1.13 (0.56, 2.28) |
| Pain | 1.27 (0.99, 1.62) | 1.16 (0.94, 1.44) | 1.42 (0.87, 2.33) | 1.24 (0.93, 1.66) |
| Psychiatric Illness | 1.79 (1.25, 2.57) | 1.24 (0.96, 1.61) | 1.18 (0.45, 3.06) | 1.34 (0.90, 2.02) |
| Short of Breath | 1.28 (0.95, 1.72) | 1.21 (0.87, 1.66) | 1.45 (0.81, 2.60) | 1.36 (0.98, 1.88) |
| Stroke | 1.18 (0.84, 1.65) | 1.28 (0.77, 2.12) | 1.10 (0.57, 2.13) | 1.05 (0.60, 1.85) |
| Wheezing | 1.15 (0.85, 1.55) | 1.33 (0.90, 1.98) | 1.22 (0.67, 2.21) | 1.13 (0.78, 1.65) |
| *Behaviors* |  |  |  |  |
| Active Smoker | 1.56 (1.15, 2.13) | 1.50 (1.02, 2.20) | 1.30 (0.81, 2.08) | 1.20 (0.80, 1.79) |
| Ever Smoked | 1.17 (0.92, 1.50) | 1.14 (0.95, 1.36) | 1.24 (0.67, 2.30) | 1.04 (0.79, 1.38) |
| Heavy Alcohol Use | 1.07 (0.76, 1.51) | 1.67 (0.56, 4.99) | 0.93 (0.44, 1.99) | 1.50 (0.66, 3.42) |
| Higher BMI | 1.13 (0.86, 1.49) | 1.04 (0.83, 1.31) | 0.92 (0.60, 1.39) | 1.10 (0.86, 1.40) |
| Low/No Vigorous Physical Activity | 1.11 (0.90, 1.35) | 1.02 (0.85, 1.22) | 1.05 (0.68, 1.61) | 1.02 (0.76, 1.37) |
| *Social Connections* |  |  |  |  |
| Ever Divorced | 1.25 (0.98, 1.60) | 1.37 (1.04, 1.80) | 0.94 (0.58, 1.52) | 1.11 (0.78, 1.58) |
| Ever Widowed | 0.84 (0.60, 1.16) | 0.86 (0.72, 1.02) | 0.86 (0.51, 1.45) | 0.91 (0.67, 1.23) |
| Less Religious | 0.98 (0.81, 1.19) | 0.91 (0.74, 1.11) | 0.96 (0.53, 1.75) | 0.62 (0.26, 1.48) |
| Lonely | 1.05 (0.76, 1.44) | 1.36 (1.08, 1.72) | 1.25 (0.81, 1.95) | 1.29 (0.92, 1.81) |
| No Friends Nearby | 0.90 (0.72, 1.13) | 1.08 (0.87, 1.33) | 1.14 (0.71, 1.83) | 1.02 (0.76, 1.38) |
| No Relatives Nearby | 1.13 (0.91, 1.41) | 0.98 (0.82, 1.17) | 1.26 (0.82, 1.96) | 1.25 (0.96, 1.65) |
| Not Married/Partnered | 1.14 (0.82, 1.59) | 1.09 (0.89, 1.33) | 1.03 (0.63, 1.67) | 1.02 (0.76, 1.36) |
| *Genetic* |  |  |  |  |
| Higher AD PGS | 0.95 (0.78, 1.15) | 1.04 (0.88, 1.23) | 1.32 (0.67, 2.61) | 1.32 (0.74, 2.38) |
| Higher Coronary Artery Disease PGS | 0.98 (0.80, 1.21) | 1.07 (0.87, 1.31) | 0.78 (0.47, 1.30) | 1.14 (0.92, 1.42) |
| Higher Diabetes PGS | 1.04 (0.86, 1.26) | 1.09 (0.88, 1.34) | 0.94 (0.54, 1.64) | 1.12 (0.77, 1.63) |
| Higher Myocardial Infarction PGS | 0.93 (0.77, 1.12) | 0.98 (0.83, 1.17) | 1.01 (0.59, 1.75) | 0.85 (0.64, 1.14) |
| Higher Parity PGS | 0.95 (0.78, 1.16) | 1.01 (0.81, 1.25) | 0.98 (0.62, 1.56) | 1.00 (0.75, 1.33) |
| Lower Age at First Birth PGS | 0.92 (0.75, 1.13) | 0.97 (0.82, 1.16) | 1.03 (0.71, 1.48) | 1.01 (0.76, 1.33) |
| Lower Age at Menarche PGS | — | 1.08 (0.92, 1.27) | — | 0.97 (0.76, 1.23) |
| Lower Age at Menopause PGS | — | 1.08 (0.93, 1.26) | — | 1.09 (0.84, 1.41) |
| Lower Education PGS | 0.93 (0.77, 1.12) | 0.97 (0.79, 1.20) | 0.96 (0.64, 1.42) | 0.88 (0.67, 1.16) |
| Lower General Cognition PGS | 0.97 (0.78, 1.19) | 1.01 (0.81, 1.26) | 0.98 (0.65, 1.48) | 1.01 (0.76, 1.34) |
| Lower Height PGS | 0.97 (0.79, 1.19) | 0.99 (0.80, 1.22) | 0.92 (0.64, 1.33) | 1.13 (0.80, 1.59) |
| Lower Longevity PGS | 1.09 (0.88, 1.35) | 1.03 (0.88, 1.22) | 1.13 (0.82, 1.56) | 0.98 (0.77, 1.26) |

**S4 Table. Subdistribution hazard ratios (sdHRs) and 95% confidence intervals (CI) of each predictor for incident dementia obtained from Fine and Gray regression models stratified by race and gender.** Models use restricted analytic sample and classify dementia using the Langa-Weir classification scheme.

|  | NH White | | NH Black | |
| --- | --- | --- | --- | --- |
| Characteristic | Men | Women | Men | Women |
| *Sociodemographic* |  |  |  |  |
| Foreign Born | 1.14 (0.73, 1.77) | 1.26 (0.81, 1.97) | 1.20 (0.39, 3.69) | 0.65 (0.23, 1.78) |
| Southern Born | 1.09 (0.87, 1.37) | 1.15 (0.95, 1.39) | 1.18 (0.50, 2.82) | 1.28 (0.83, 1.98) |
| Veteran | 0.85 (0.68, 1.06) | 0.36 (0.09, 1.35) | 0.69 (0.44, 1.09) | 1.13 (0.22, 5.67) |
| Childless | 0.98 (0.60, 1.60) | 0.73 (0.38, 1.41) | 1.17 (0.34, 3.95) | 0.72 (0.56, 0.94) |
| Higher Parity | 1.03 (0.83, 1.29) | 1.08 (0.90, 1.31) | 1.13 (0.92, 1.40) | 1.15 (0.96, 1.38) |
| Lower Age at First Birth | 1.06 (0.85, 1.33) | 1.30 (1.05, 1.60) | 1.02 (0.68, 1.52) | 1.11 (0.87, 1.41) |
| Higher Age at Last Birth | 1.07 (0.86, 1.33) | 1.12 (0.94, 1.34) | 0.88 (0.63, 1.22) | 0.98 (0.79, 1.22) |
| *Early-Life* |  |  |  |  |
| Lower Mother's Education | 1.19 (0.92, 1.55) | 1.13 (0.88, 1.46) | 1.26 (0.86, 1.85) | 1.25 (0.90, 1.74) |
| Lower Father's Education | 1.15 (0.93, 1.44) | 1.09 (0.84, 1.42) | 1.39 (0.91, 2.12) | 1.25 (0.91, 1.72) |
| Lower Father's Occupational Status | 1.15 (0.92, 1.43) | 1.12 (0.94, 1.33) | 2.67 (0.61, 11.66) | 1.72 (0.77, 3.87) |
| Lower SR Childhood Health | 1.23 (1.01, 1.50) | 1.21 (0.92, 1.59) | 1.16 (0.76, 1.77) | 1.18 (0.91, 1.51) |
| Lower SR Childhood SES | 1.02 (0.84, 1.24) | 1.10 (0.90, 1.35) | 1.19 (0.72, 1.96) | 1.17 (0.88, 1.57) |
| Lower Education | 1.55 (1.28, 1.87) | 1.34 (1.09, 1.65) | 1.61 (1.01, 2.57) | 1.47 (1.11, 1.94) |
| *Economic* |  |  |  |  |
| Food Insecurity | 0.73 (0.36, 1.45) | 0.74 (0.49, 1.13) | 0.77 (0.44, 1.37) | 0.65 (0.48, 0.87) |
| Lower Income | 1.22 (1.03, 1.43) | 1.38 (1.16, 1.63) | 1.07 (0.83, 1.39) | 1.19 (1.08, 1.31) |
| Lower Neighborhood Safety | 1.44 (0.93, 2.23) | 1.33 (0.64, 2.77) | 0.89 (0.52, 1.54) | 1.24 (0.92, 1.67) |
| Lower Wealth | 1.28 (1.00, 1.63) | 1.14 (0.84, 1.53) | 1.12 (0.88, 1.42) | 1.13 (1.00, 1.28) |
| Medicaid | 1.94 (0.97, 3.88) | 1.03 (0.64, 1.67) | 1.44 (0.73, 2.82) | 1.36 (1.00, 1.85) |
| Medicare | 0.71 (0.56, 0.91) | 0.75 (0.53, 1.06) | 0.73 (0.45, 1.19) | 0.83 (0.60, 1.15) |
| No Insurance | 0.94 (0.76, 1.15) | 0.94 (0.78, 1.13) | 0.67 (0.42, 1.06) | 0.73 (0.51, 1.03) |
| Received Food Stamps | 1.89 (0.64, 5.57) | 1.44 (0.47, 4.48) | 1.42 (0.87, 2.33) | 1.49 (1.05, 2.12) |
| Retired | 0.83 (0.65, 1.05) | 0.92 (0.77, 1.12) | 0.72 (0.43, 1.21) | 0.83 (0.62, 1.09) |
| Unemployed | 1.08 (0.29, 4.06) | 0.98 (0.24, 4.03) | 0.00 (0.00, 0.00) | 1.61 (0.89, 2.93) |
| *Health* |  |  |  |  |
| Arthritis | 0.89 (0.73, 1.09) | 1.11 (0.93, 1.33) | 0.99 (0.63, 1.56) | 0.93 (0.70, 1.24) |
| Back Pain | 1.09 (0.87, 1.36) | 0.99 (0.80, 1.23) | 1.18 (0.73, 1.92) | 0.85 (0.62, 1.15) |
| Cancer | 0.89 (0.66, 1.18) | 0.81 (0.63, 1.05) | 0.62 (0.30, 1.27) | 0.97 (0.59, 1.59) |
| Diabetes | 1.04 (0.79, 1.38) | 1.07 (0.82, 1.39) | 1.18 (0.63, 2.22) | 1.13 (0.81, 1.56) |
| Dizziness | 1.22 (0.88, 1.70) | 1.18 (0.83, 1.67) | 1.12 (0.60, 2.07) | 1.51 (1.07, 2.12) |
| Fatigue | 1.20 (0.85, 1.69) | 1.25 (0.96, 1.61) | 0.88 (0.42, 1.83) | 1.25 (0.88, 1.79) |
| Headaches | 1.14 (0.66, 1.96) | 1.69 (1.15, 2.49) | 1.12 (0.64, 1.99) | 1.36 (0.85, 2.19) |
| Heart Problems | 1.00 (0.80, 1.26) | 0.97 (0.78, 1.20) | 0.69 (0.31, 1.52) | 1.18 (0.81, 1.73) |
| Hypertension | 0.93 (0.75, 1.14) | 1.03 (0.86, 1.25) | 0.94 (0.59, 1.49) | 1.27 (0.93, 1.74) |
| Lower SR Health | 1.29 (1.03, 1.62) | 1.26 (0.96, 1.65) | 1.13 (0.72, 1.76) | 1.38 (1.01, 1.89) |
| Lower SR Hearing | 1.15 (0.92, 1.44) | 1.07 (0.86, 1.33) | 1.07 (0.69, 1.67) | 0.93 (0.68, 1.29) |
| Lower SR Vision | 1.11 (0.90, 1.36) | 1.10 (0.92, 1.32) | 1.27 (0.74, 2.17) | 1.04 (0.79, 1.38) |
| Lung Disease | 0.75 (0.45, 1.25) | 1.15 (0.60, 2.20) | 1.36 (0.58, 3.15) | 1.14 (0.51, 2.56) |
| Pain | 1.23 (0.97, 1.57) | 1.13 (0.91, 1.39) | 1.16 (0.67, 2.01) | 1.12 (0.83, 1.51) |
| Psychiatric Illness | 1.57 (1.07, 2.31) | 1.20 (0.94, 1.53) | 0.79 (0.23, 2.67) | 1.37 (0.89, 2.11) |
| Short of Breath | 1.18 (0.88, 1.59) | 1.11 (0.80, 1.55) | 1.39 (0.77, 2.51) | 1.17 (0.83, 1.66) |
| Stroke | 1.13 (0.80, 1.59) | 1.27 (0.77, 2.10) | 1.00 (0.49, 2.05) | 0.89 (0.47, 1.70) |
| Wheezing | 1.13 (0.84, 1.51) | 1.17 (0.77, 1.77) | 1.09 (0.59, 2.05) | 1.13 (0.77, 1.66) |
| *Behaviors* |  |  |  |  |
| Active Smoker | 1.24 (0.90, 1.72) | 1.15 (0.75, 1.78) | 1.11 (0.64, 1.93) | 1.04 (0.65, 1.66) |
| Ever Smoked | 1.13 (0.89, 1.44) | 1.02 (0.85, 1.22) | 1.27 (0.65, 2.51) | 1.00 (0.75, 1.32) |
| Heavy Alcohol Use | 1.02 (0.71, 1.45) | 1.62 (0.54, 4.86) | 0.86 (0.37, 2.02) | 1.19 (0.56, 2.53) |
| Higher BMI | 1.01 (0.77, 1.32) | 0.99 (0.81, 1.21) | 0.92 (0.56, 1.52) | 1.00 (0.78, 1.28) |
| Low/No Vigorous Physical Activity | 1.06 (0.87, 1.30) | 1.02 (0.85, 1.22) | 0.99 (0.62, 1.58) | 0.98 (0.73, 1.33) |
| *Social Connections* |  |  |  |  |
| Ever Divorced | 1.10 (0.86, 1.41) | 1.21 (0.91, 1.60) | 0.92 (0.54, 1.56) | 1.07 (0.75, 1.54) |
| Ever Widowed | 0.90 (0.66, 1.24) | 0.91 (0.77, 1.08) | 0.86 (0.50, 1.46) | 0.98 (0.73, 1.31) |
| Less Religious | 1.00 (0.82, 1.21) | 0.92 (0.75, 1.13) | 1.04 (0.55, 1.94) | 0.61 (0.28, 1.34) |
| Lonely | 1.00 (0.73, 1.38) | 1.35 (1.07, 1.70) | 1.30 (0.83, 2.05) | 1.26 (0.88, 1.81) |
| No Friends Nearby | 0.93 (0.74, 1.17) | 1.11 (0.90, 1.37) | 1.32 (0.79, 2.20) | 1.06 (0.77, 1.46) |
| No Relatives Nearby | 1.13 (0.91, 1.41) | 1.02 (0.85, 1.21) | 1.12 (0.69, 1.82) | 1.22 (0.93, 1.61) |
| Not Married/Partnered | 1.11 (0.80, 1.54) | 1.03 (0.86, 1.25) | 1.01 (0.60, 1.68) | 1.02 (0.76, 1.37) |
| *Genetic* |  |  |  |  |
| Higher AD PGS | 0.95 (0.79, 1.16) | 1.05 (0.88, 1.24) | 1.00 (0.49, 2.02) | 1.42 (0.78, 2.58) |
| Higher Coronary Artery Disease PGS | 0.95 (0.77, 1.17) | 1.06 (0.87, 1.30) | 0.78 (0.43, 1.44) | 1.14 (0.91, 1.43) |
| Higher Diabetes PGS | 1.05 (0.87, 1.27) | 1.11 (0.89, 1.37) | 0.96 (0.53, 1.72) | 1.07 (0.73, 1.57) |
| Higher Myocardial Infarction PGS | 0.91 (0.75, 1.10) | 0.99 (0.83, 1.17) | 0.96 (0.53, 1.73) | 0.85 (0.63, 1.14) |
| Higher Parity PGS | 0.98 (0.80, 1.20) | 1.03 (0.83, 1.28) | 1.02 (0.61, 1.71) | 0.99 (0.74, 1.33) |
| Lower Age at First Birth PGS | 0.93 (0.75, 1.14) | 0.98 (0.82, 1.16) | 1.02 (0.67, 1.54) | 1.01 (0.76, 1.35) |
| Lower Age at Menarche PGS | — | 1.11 (0.95, 1.31) | — | 0.97 (0.75, 1.25) |
| Lower Age at Menopause PGS | — | 1.09 (0.94, 1.27) | — | 1.02 (0.79, 1.31) |
| Lower Education PGS | 0.93 (0.77, 1.13) | 0.97 (0.79, 1.20) | 0.96 (0.62, 1.48) | 0.91 (0.70, 1.18) |
| Lower General Cognition PGS | 0.96 (0.77, 1.18) | 1.01 (0.81, 1.25) | 0.96 (0.60, 1.54) | 1.07 (0.81, 1.42) |
| Lower Height PGS | 0.97 (0.79, 1.19) | 0.97 (0.79, 1.20) | 0.92 (0.61, 1.39) | 1.11 (0.79, 1.56) |
| Lower Longevity PGS | 1.07 (0.86, 1.34) | 1.04 (0.88, 1.23) | 1.15 (0.83, 1.61) | 1.01 (0.78, 1.30) |

**S5 Table. Subdistribution hazard ratios (sdHRs) and 95% confidence intervals (CI) of each predictor for incident dementia obtained from Fine and Gray regression models stratified by race and gender.** Models use restricted analytic sample and classify dementia using the Hurd classification scheme.

|  | NH White | | NH Black | |
| --- | --- | --- | --- | --- |
| Characteristic | Men | Women | Men | Women |
| *Sociodemographic* |  |  |  |  |
| Foreign Born | 1.06 (0.78, 1.45) | 1.03 (0.80, 1.34) | 0.76 (0.20, 2.95) | 0.76 (0.29, 2.00) |
| Southern Born | 1.12 (0.94, 1.33) | 1.04 (0.91, 1.20) | 1.37 (0.68, 2.75) | 1.19 (0.75, 1.90) |
| Veteran | 0.97 (0.81, 1.15) | 0.79 (0.39, 1.59) | 0.88 (0.55, 1.41) | 0.00 (0.00, 0.00) |
| Childless | 0.95 (0.63, 1.45) | 0.81 (0.49, 1.34) | 1.40 (0.40, 4.92) | 0.71 (0.54, 0.93) |
| Higher Parity | 0.98 (0.83, 1.16) | 0.97 (0.85, 1.11) | 1.17 (0.93, 1.47) | 1.15 (0.95, 1.40) |
| Lower Age at First Birth | 1.01 (0.87, 1.18) | 1.15 (1.00, 1.32) | 1.40 (0.89, 2.18) | 0.95 (0.75, 1.22) |
| Higher Age at Last Birth | 1.03 (0.88, 1.20) | 1.14 (1.00, 1.31) | 1.00 (0.68, 1.47) | 0.85 (0.67, 1.09) |
| *Early-Life* |  |  |  |  |
| Lower Mother's Education | 1.23 (1.02, 1.48) | 1.06 (0.88, 1.28) | 1.07 (0.67, 1.72) | 1.16 (0.81, 1.67) |
| Lower Father's Education | 1.19 (1.00, 1.42) | 1.01 (0.84, 1.22) | 1.06 (0.57, 1.98) | 1.19 (0.88, 1.62) |
| Lower Father's Occupational Status | 1.16 (0.99, 1.36) | 1.10 (0.96, 1.26) | 1.60 (0.56, 4.54) | 0.90 (0.45, 1.83) |
| Lower SR Childhood Health | 1.10 (0.94, 1.29) | 1.07 (0.95, 1.21) | 1.14 (0.74, 1.74) | 1.08 (0.82, 1.43) |
| Lower SR Childhood SES | 1.06 (0.92, 1.22) | 1.11 (0.98, 1.26) | 0.91 (0.55, 1.51) | 1.18 (0.88, 1.60) |
| Lower Education | 1.29 (1.12, 1.47) | 1.10 (0.97, 1.25) | 1.22 (0.80, 1.86) | 1.27 (0.97, 1.67) |
| *Economic* |  |  |  |  |
| Food Insecurity | 0.76 (0.40, 1.45) | 0.82 (0.57, 1.16) | 1.06 (0.47, 2.37) | 0.80 (0.53, 1.21) |
| Lower Income | 1.14 (0.96, 1.36) | 1.11 (0.98, 1.27) | 1.12 (0.85, 1.46) | 1.03 (0.83, 1.28) |
| Lower Neighborhood Safety | 1.27 (0.88, 1.83) | 1.04 (0.74, 1.45) | 0.72 (0.40, 1.27) | 0.98 (0.69, 1.39) |
| Lower Wealth | 1.21 (0.95, 1.54) | 1.04 (0.89, 1.22) | 1.05 (0.77, 1.42) | 1.13 (0.98, 1.30) |
| Medicaid | 1.72 (0.95, 3.11) | 1.07 (0.73, 1.59) | 1.54 (0.63, 3.72) | 1.23 (0.88, 1.73) |
| Medicare | 0.97 (0.76, 1.23) | 0.94 (0.74, 1.18) | 1.03 (0.60, 1.80) | 0.99 (0.66, 1.50) |
| No Insurance | 0.95 (0.82, 1.09) | 1.00 (0.88, 1.13) | 0.69 (0.43, 1.11) | 0.78 (0.54, 1.12) |
| Received Food Stamps | 2.62 (1.46, 4.68) | 0.92 (0.53, 1.59) | 0.91 (0.29, 2.84) | 1.28 (0.80, 2.06) |
| Retired | 0.95 (0.77, 1.17) | 0.92 (0.81, 1.04) | 0.90 (0.49, 1.67) | 0.96 (0.70, 1.33) |
| Unemployed | 0.50 (0.11, 2.25) | 1.07 (0.39, 2.93) | 0.00 (0.00, 0.00) | 1.70 (0.85, 3.40) |
| *Health* |  |  |  |  |
| Arthritis | 1.00 (0.86, 1.15) | 1.05 (0.93, 1.19) | 0.98 (0.62, 1.56) | 0.95 (0.71, 1.27) |
| Back Pain | 1.07 (0.91, 1.26) | 0.96 (0.84, 1.09) | 1.24 (0.75, 2.07) | 0.94 (0.68, 1.30) |
| Cancer | 0.95 (0.78, 1.15) | 0.87 (0.72, 1.05) | 0.84 (0.43, 1.64) | 0.96 (0.55, 1.67) |
| Diabetes | 1.11 (0.91, 1.35) | 1.04 (0.84, 1.29) | 1.32 (0.85, 2.03) | 1.05 (0.75, 1.47) |
| Dizziness | 1.26 (1.01, 1.59) | 1.02 (0.84, 1.24) | 1.13 (0.54, 2.38) | 1.43 (0.91, 2.22) |
| Fatigue | 1.23 (0.95, 1.58) | 1.08 (0.92, 1.27) | 1.06 (0.47, 2.41) | 1.30 (0.88, 1.92) |
| Headaches | 1.14 (0.78, 1.66) | 1.30 (1.03, 1.63) | 1.24 (0.68, 2.26) | 1.62 (0.99, 2.67) |
| Heart Problems | 1.02 (0.87, 1.20) | 1.02 (0.87, 1.21) | 0.61 (0.30, 1.25) | 1.15 (0.74, 1.78) |
| Hypertension | 0.94 (0.81, 1.09) | 1.05 (0.93, 1.18) | 0.81 (0.51, 1.29) | 1.28 (0.93, 1.76) |
| Lower SR Health | 1.21 (1.02, 1.43) | 1.12 (0.97, 1.29) | 0.99 (0.62, 1.58) | 1.32 (0.93, 1.87) |
| Lower SR Hearing | 1.16 (0.99, 1.36) | 1.04 (0.91, 1.17) | 1.07 (0.65, 1.79) | 0.99 (0.69, 1.43) |
| Lower SR Vision | 1.12 (0.97, 1.30) | 1.07 (0.95, 1.21) | 1.24 (0.71, 2.17) | 0.96 (0.72, 1.28) |
| Lung Disease | 0.82 (0.58, 1.15) | 0.96 (0.73, 1.27) | 1.27 (0.44, 3.67) | 1.35 (0.71, 2.56) |
| Pain | 1.17 (0.98, 1.40) | 1.05 (0.92, 1.20) | 1.13 (0.64, 1.98) | 1.17 (0.84, 1.63) |
| Psychiatric Illness | 1.32 (0.91, 1.90) | 1.19 (0.97, 1.47) | 0.59 (0.12, 2.86) | 1.09 (0.62, 1.92) |
| Short of Breath | 1.18 (0.95, 1.47) | 1.02 (0.85, 1.22) | 1.38 (0.78, 2.46) | 1.38 (0.94, 2.01) |
| Stroke | 1.15 (0.87, 1.52) | 1.09 (0.86, 1.38) | 1.17 (0.52, 2.63) | 1.09 (0.65, 1.84) |
| Wheezing | 1.11 (0.90, 1.38) | 1.04 (0.85, 1.28) | 0.77 (0.33, 1.81) | 1.18 (0.77, 1.80) |
| *Behaviors* |  |  |  |  |
| Active Smoker | 1.02 (0.76, 1.36) | 0.99 (0.79, 1.25) | 1.06 (0.59, 1.90) | 1.05 (0.62, 1.78) |
| Ever Smoked | 1.05 (0.89, 1.23) | 1.03 (0.91, 1.16) | 1.13 (0.64, 2.00) | 1.02 (0.75, 1.40) |
| Heavy Alcohol Use | 0.98 (0.76, 1.27) | 1.15 (0.80, 1.64) | 0.88 (0.37, 2.13) | 1.03 (0.35, 3.05) |
| Higher BMI | 1.09 (0.89, 1.34) | 1.01 (0.88, 1.16) | 0.95 (0.53, 1.68) | 1.03 (0.80, 1.33) |
| Low/No Vigorous Physical Activity | 1.09 (0.94, 1.26) | 1.03 (0.91, 1.17) | 0.83 (0.52, 1.32) | 0.96 (0.70, 1.31) |
| *Social Connections* |  |  |  |  |
| Ever Divorced | 0.98 (0.79, 1.21) | 1.11 (0.94, 1.32) | 0.83 (0.48, 1.45) | 0.94 (0.61, 1.44) |
| Ever Widowed | 0.95 (0.77, 1.17) | 0.98 (0.87, 1.11) | 0.96 (0.59, 1.57) | 1.06 (0.78, 1.44) |
| Less Religious | 0.94 (0.82, 1.07) | 0.94 (0.81, 1.08) | 0.69 (0.32, 1.47) | 0.58 (0.26, 1.30) |
| Lonely | 1.06 (0.84, 1.34) | 1.13 (0.98, 1.31) | 1.61 (1.04, 2.50) | 1.43 (1.03, 1.98) |
| No Friends Nearby | 1.04 (0.87, 1.24) | 0.97 (0.84, 1.12) | 0.99 (0.59, 1.64) | 1.23 (0.87, 1.76) |
| No Relatives Nearby | 1.08 (0.92, 1.27) | 1.05 (0.93, 1.19) | 0.93 (0.53, 1.62) | 1.18 (0.87, 1.59) |
| Not Married/Partnered | 1.03 (0.83, 1.28) | 1.00 (0.89, 1.12) | 1.04 (0.64, 1.69) | 1.02 (0.75, 1.39) |
| *Genetic* |  |  |  |  |
| Higher AD PGS | 1.02 (0.89, 1.18) | 1.04 (0.93, 1.16) | 0.74 (0.33, 1.69) | 1.41 (0.74, 2.68) |
| Higher Coronary Artery Disease PGS | 0.99 (0.86, 1.14) | 0.99 (0.87, 1.12) | 0.94 (0.65, 1.35) | 1.10 (0.86, 1.42) |
| Higher Diabetes PGS | 1.03 (0.90, 1.19) | 1.04 (0.93, 1.16) | 0.83 (0.47, 1.48) | 0.90 (0.58, 1.39) |
| Higher Myocardial Infarction PGS | 0.96 (0.84, 1.10) | 1.00 (0.88, 1.14) | 0.79 (0.48, 1.28) | 0.86 (0.62, 1.18) |
| Higher Parity PGS | 0.94 (0.82, 1.07) | 1.06 (0.94, 1.20) | 0.85 (0.58, 1.24) | 0.80 (0.59, 1.09) |
| Lower Age at First Birth PGS | 1.00 (0.86, 1.16) | 1.02 (0.90, 1.16) | 1.09 (0.69, 1.72) | 1.13 (0.84, 1.52) |
| Lower Age at Menarche PGS | — | 1.05 (0.94, 1.18) | — | 0.95 (0.70, 1.28) |
| Lower Age at Menopause PGS | — | 0.99 (0.88, 1.11) | — | 1.01 (0.76, 1.34) |
| Lower Education PGS | 0.98 (0.85, 1.13) | 1.02 (0.91, 1.15) | 0.79 (0.51, 1.21) | 0.96 (0.74, 1.23) |
| Lower General Cognition PGS | 0.97 (0.83, 1.12) | 1.08 (0.95, 1.21) | 0.87 (0.58, 1.31) | 0.99 (0.73, 1.34) |
| Lower Height PGS | 0.96 (0.83, 1.11) | 1.04 (0.93, 1.16) | 0.79 (0.48, 1.28) | 1.13 (0.78, 1.63) |
| Lower Longevity PGS | 1.02 (0.88, 1.19) | 1.00 (0.89, 1.12) | 1.11 (0.82, 1.51) | 0.91 (0.70, 1.17) |

**S6 Table. Subdistribution hazard ratios (sdHRs) and 95% confidence intervals (CI) of each predictor for incident dementia obtained from Fine and Gray regression models stratified by race and gender.** Models use restricted analytic sample and classify dementia using the Expert classification scheme.

|  | NH White | | NH Black | |
| --- | --- | --- | --- | --- |
| Characteristic | Men | Women | Men | Women |
| *Sociodemographic* |  |  |  |  |
| Foreign Born | 1.07 (0.79, 1.45) | 1.09 (0.85, 1.39) | 0.86 (0.26, 2.85) | 1.02 (0.68, 1.52) |
| Southern Born | 1.10 (0.94, 1.29) | 1.02 (0.90, 1.16) | 1.37 (0.71, 2.63) | 1.04 (0.78, 1.38) |
| Veteran | 0.88 (0.75, 1.04) | 0.87 (0.49, 1.53) | 0.99 (0.63, 1.54) | 0.94 (0.35, 2.53) |
| Childless | 0.78 (0.51, 1.19) | 0.82 (0.51, 1.31) | 1.93 (0.80, 4.61) | 0.29 (0.04, 2.00) |
| Higher Parity | 0.96 (0.81, 1.15) | 1.04 (0.93, 1.17) | 1.08 (0.85, 1.37) | 1.19 (1.01, 1.40) |
| Lower Age at First Birth | 1.00 (0.86, 1.16) | 1.17 (1.03, 1.33) | 1.37 (0.85, 2.20) | 1.08 (0.87, 1.35) |
| Higher Age at Last Birth | 0.99 (0.86, 1.14) | 1.08 (0.95, 1.22) | 1.05 (0.74, 1.50) | 0.93 (0.76, 1.14) |
| *Early-Life* |  |  |  |  |
| Lower Mother's Education | 1.31 (1.08, 1.59) | 1.17 (0.97, 1.41) | 1.05 (0.64, 1.73) | 1.13 (0.83, 1.54) |
| Lower Father's Education | 1.23 (1.02, 1.48) | 1.15 (0.95, 1.38) | 1.05 (0.61, 1.82) | 1.07 (0.82, 1.41) |
| Lower Father's Occupational Status | 1.20 (1.03, 1.39) | 1.17 (1.03, 1.32) | 1.34 (0.51, 3.53) | 0.96 (0.52, 1.77) |
| Lower SR Childhood Health | 1.09 (0.95, 1.26) | 1.08 (0.96, 1.21) | 1.12 (0.74, 1.68) | 1.10 (0.87, 1.40) |
| Lower SR Childhood SES | 1.07 (0.94, 1.22) | 1.13 (1.00, 1.27) | 1.06 (0.64, 1.76) | 1.03 (0.83, 1.28) |
| Lower Education | 1.35 (1.19, 1.53) | 1.17 (1.03, 1.32) | 1.03 (0.71, 1.50) | 1.08 (0.86, 1.35) |
| *Economic* |  |  |  |  |
| Food Insecurity | 0.93 (0.49, 1.78) | 0.85 (0.62, 1.17) | 1.02 (0.48, 2.17) | 0.75 (0.55, 1.00) |
| Lower Income | 1.17 (1.01, 1.35) | 1.15 (1.01, 1.31) | 1.09 (0.83, 1.42) | 1.01 (0.83, 1.23) |
| Lower Neighborhood Safety | 1.27 (0.92, 1.75) | 1.17 (0.85, 1.61) | 0.73 (0.41, 1.27) | 1.04 (0.78, 1.40) |
| Lower Wealth | 1.16 (0.92, 1.46) | 1.05 (0.90, 1.22) | 1.13 (0.87, 1.46) | 1.07 (0.94, 1.22) |
| Medicaid | 1.42 (0.77, 2.61) | 1.15 (0.80, 1.65) | 1.57 (0.71, 3.47) | 1.17 (0.86, 1.59) |
| Medicare | 0.97 (0.78, 1.20) | 0.94 (0.76, 1.16) | 0.86 (0.53, 1.42) | 1.00 (0.70, 1.44) |
| No Insurance | 0.94 (0.82, 1.07) | 0.99 (0.89, 1.11) | 0.71 (0.45, 1.13) | 0.83 (0.64, 1.08) |
| Received Food Stamps | 2.04 (1.19, 3.50) | 0.98 (0.60, 1.60) | 0.95 (0.33, 2.77) | 1.54 (1.11, 2.14) |
| Retired | 0.96 (0.79, 1.16) | 0.93 (0.83, 1.04) | 0.86 (0.49, 1.52) | 0.91 (0.69, 1.20) |
| Unemployed | 0.63 (0.24, 1.68) | 1.23 (0.52, 2.91) | 0.00 (0.00, 0.00) | 1.35 (0.73, 2.52) |
| *Health* |  |  |  |  |
| Arthritis | 1.00 (0.88, 1.15) | 1.04 (0.92, 1.16) | 1.04 (0.66, 1.63) | 1.06 (0.81, 1.37) |
| Back Pain | 1.07 (0.92, 1.25) | 0.97 (0.85, 1.09) | 1.37 (0.85, 2.21) | 1.04 (0.80, 1.34) |
| Cancer | 0.91 (0.76, 1.10) | 0.93 (0.79, 1.10) | 0.81 (0.42, 1.55) | 0.93 (0.59, 1.49) |
| Diabetes | 1.24 (1.03, 1.50) | 1.19 (0.98, 1.44) | 1.28 (0.85, 1.93) | 1.21 (0.86, 1.71) |
| Dizziness | 1.24 (1.01, 1.52) | 1.03 (0.85, 1.25) | 1.03 (0.55, 1.92) | 1.42 (0.98, 2.07) |
| Fatigue | 1.27 (0.98, 1.65) | 1.06 (0.91, 1.23) | 0.94 (0.42, 2.09) | 1.26 (0.90, 1.75) |
| Headaches | 1.08 (0.75, 1.57) | 1.26 (1.02, 1.56) | 1.30 (0.79, 2.14) | 1.34 (0.86, 2.10) |
| Heart Problems | 1.03 (0.88, 1.21) | 1.01 (0.87, 1.17) | 0.56 (0.26, 1.20) | 1.23 (0.93, 1.62) |
| Hypertension | 0.98 (0.85, 1.12) | 1.02 (0.92, 1.15) | 0.87 (0.55, 1.37) | 1.25 (0.96, 1.63) |
| Lower SR Health | 1.23 (1.05, 1.44) | 1.15 (1.02, 1.30) | 0.96 (0.63, 1.47) | 1.42 (1.08, 1.86) |
| Lower SR Hearing | 1.18 (1.01, 1.38) | 1.07 (0.95, 1.20) | 0.96 (0.61, 1.51) | 0.92 (0.67, 1.25) |
| Lower SR Vision | 1.15 (0.99, 1.33) | 1.10 (0.99, 1.23) | 1.23 (0.73, 2.07) | 1.03 (0.78, 1.35) |
| Lung Disease | 0.92 (0.69, 1.23) | 1.04 (0.81, 1.34) | 1.43 (0.61, 3.36) | 1.40 (0.77, 2.55) |
| Pain | 1.14 (0.95, 1.37) | 1.05 (0.93, 1.19) | 1.17 (0.68, 2.01) | 1.16 (0.87, 1.53) |
| Psychiatric Illness | 1.32 (0.95, 1.83) | 1.22 (0.99, 1.51) | 0.73 (0.18, 2.93) | 1.42 (0.88, 2.30) |
| Short of Breath | 1.12 (0.90, 1.40) | 0.98 (0.83, 1.17) | 1.41 (0.88, 2.26) | 1.33 (0.97, 1.83) |
| Stroke | 1.16 (0.89, 1.52) | 1.09 (0.88, 1.35) | 1.05 (0.50, 2.20) | 1.24 (0.85, 1.81) |
| Wheezing | 1.12 (0.91, 1.39) | 1.00 (0.82, 1.23) | 0.76 (0.36, 1.61) | 1.04 (0.70, 1.55) |
| *Behaviors* |  |  |  |  |
| Active Smoker | 1.13 (0.88, 1.45) | 1.02 (0.83, 1.27) | 1.06 (0.60, 1.86) | 1.05 (0.71, 1.57) |
| Ever Smoked | 1.11 (0.95, 1.29) | 1.01 (0.90, 1.13) | 1.27 (0.71, 2.28) | 1.02 (0.79, 1.31) |
| Heavy Alcohol Use | 0.98 (0.78, 1.24) | 1.09 (0.76, 1.56) | 1.01 (0.46, 2.25) | 0.81 (0.31, 2.12) |
| Higher BMI | 1.14 (0.95, 1.37) | 1.06 (0.94, 1.19) | 0.96 (0.58, 1.59) | 1.05 (0.87, 1.27) |
| Low/No Vigorous Physical Activity | 1.10 (0.96, 1.26) | 1.02 (0.91, 1.15) | 0.94 (0.60, 1.48) | 0.90 (0.71, 1.14) |
| *Social Connections* |  |  |  |  |
| Ever Divorced | 1.04 (0.86, 1.27) | 1.12 (0.95, 1.32) | 1.05 (0.64, 1.73) | 1.26 (0.98, 1.62) |
| Ever Widowed | 1.01 (0.84, 1.23) | 0.97 (0.87, 1.08) | 0.93 (0.58, 1.48) | 0.94 (0.73, 1.22) |
| Less Religious | 0.95 (0.84, 1.07) | 0.95 (0.84, 1.08) | 0.85 (0.42, 1.70) | 0.83 (0.54, 1.27) |
| Lonely | 1.11 (0.86, 1.42) | 1.14 (0.99, 1.31) | 1.65 (1.14, 2.38) | 1.33 (0.97, 1.81) |
| No Friends Nearby | 1.00 (0.85, 1.19) | 0.94 (0.83, 1.07) | 1.12 (0.68, 1.86) | 1.07 (0.80, 1.44) |
| No Relatives Nearby | 1.12 (0.96, 1.30) | 1.01 (0.90, 1.14) | 0.95 (0.57, 1.60) | 1.14 (0.88, 1.47) |
| Not Married/Partnered | 0.92 (0.75, 1.13) | 1.01 (0.90, 1.13) | 0.93 (0.59, 1.47) | 0.97 (0.73, 1.29) |
| *Genetic* |  |  |  |  |
| Higher AD PGS | 1.04 (0.91, 1.18) | 1.02 (0.92, 1.14) | 1.01 (0.44, 2.33) | 1.45 (0.83, 2.51) |
| Higher Coronary Artery Disease PGS | 0.99 (0.88, 1.13) | 0.99 (0.88, 1.11) | 0.86 (0.58, 1.25) | 1.12 (0.89, 1.41) |
| Higher Diabetes PGS | 1.04 (0.91, 1.19) | 1.03 (0.92, 1.14) | 0.83 (0.45, 1.52) | 0.84 (0.59, 1.19) |
| Higher Myocardial Infarction PGS | 1.01 (0.88, 1.15) | 0.99 (0.88, 1.11) | 0.80 (0.49, 1.29) | 0.88 (0.69, 1.14) |
| Higher Parity PGS | 1.03 (0.90, 1.18) | 1.02 (0.91, 1.15) | 0.85 (0.57, 1.27) | 0.95 (0.76, 1.21) |
| Lower Age at First Birth PGS | 0.99 (0.86, 1.14) | 1.00 (0.89, 1.12) | 1.10 (0.70, 1.74) | 1.05 (0.83, 1.34) |
| Lower Age at Menarche PGS | — | 1.00 (0.90, 1.11) | — | 0.96 (0.74, 1.24) |
| Lower Age at Menopause PGS | — | 1.01 (0.91, 1.13) | — | 0.99 (0.78, 1.25) |
| Lower Education PGS | 1.03 (0.90, 1.18) | 0.99 (0.89, 1.10) | 0.79 (0.52, 1.20) | 0.96 (0.76, 1.21) |
| Lower General Cognition PGS | 0.96 (0.84, 1.10) | 1.05 (0.94, 1.17) | 0.80 (0.54, 1.19) | 0.97 (0.77, 1.23) |
| Lower Height PGS | 0.98 (0.86, 1.12) | 1.05 (0.94, 1.17) | 0.81 (0.51, 1.30) | 1.11 (0.82, 1.50) |
| Lower Longevity PGS | 0.97 (0.84, 1.13) | 1.01 (0.91, 1.13) | 1.07 (0.80, 1.43) | 0.92 (0.74, 1.16) |

**S7 Table. Subdistribution hazard ratios (sdHRs) and 95% confidence intervals (CI) of each predictor for incident dementia obtained from Fine and Gray regression models stratified by race and gender.** Models use restricted analytic sample and classify dementia using the LASSO classification scheme.

|  | NH White | | NH Black | |
| --- | --- | --- | --- | --- |
| Characteristic | Men | Women | Men | Women |
| *Sociodemographic* |  |  |  |  |
| Foreign Born | 1.09 (0.81, 1.47) | 0.96 (0.73, 1.27) | 1.04 (0.33, 3.23) | 1.04 (0.71, 1.52) |
| Southern Born | 1.17 (0.99, 1.38) | 1.09 (0.96, 1.25) | 1.23 (0.64, 2.38) | 1.09 (0.80, 1.47) |
| Veteran | 0.86 (0.73, 1.02) | 0.98 (0.57, 1.68) | 1.01 (0.65, 1.58) | 0.00 (0.00, 0.00) |
| Childless | 0.98 (0.67, 1.43) | 0.89 (0.56, 1.41) | 1.07 (0.25, 4.49) | 0.73 (0.47, 1.16) |
| Higher Parity | 0.98 (0.84, 1.15) | 0.98 (0.86, 1.12) | 1.21 (0.98, 1.49) | 1.12 (0.93, 1.35) |
| Lower Age at First Birth | 0.98 (0.85, 1.13) | 1.14 (0.99, 1.31) | 1.42 (0.91, 2.22) | 1.07 (0.85, 1.36) |
| Higher Age at Last Birth | 0.98 (0.85, 1.13) | 1.08 (0.95, 1.23) | 0.97 (0.68, 1.39) | 0.98 (0.80, 1.21) |
| *Early-Life* |  |  |  |  |
| Lower Mother's Education | 1.26 (1.05, 1.51) | 1.11 (0.92, 1.33) | 1.11 (0.66, 1.87) | 1.07 (0.79, 1.45) |
| Lower Father's Education | 1.16 (0.96, 1.41) | 1.12 (0.93, 1.35) | 1.17 (0.62, 2.20) | 0.97 (0.72, 1.30) |
| Lower Father's Occupational Status | 1.14 (0.98, 1.33) | 1.17 (1.02, 1.34) | 1.15 (0.61, 2.18) | 0.91 (0.57, 1.45) |
| Lower SR Childhood Health | 1.13 (0.98, 1.30) | 1.09 (0.97, 1.22) | 1.17 (0.77, 1.77) | 1.05 (0.80, 1.37) |
| Lower SR Childhood SES | 1.05 (0.92, 1.21) | 1.15 (1.02, 1.30) | 0.87 (0.51, 1.49) | 1.13 (0.89, 1.44) |
| Lower Education | 1.39 (1.21, 1.59) | 1.17 (1.03, 1.32) | 1.16 (0.80, 1.70) | 1.12 (0.88, 1.41) |
| *Economic* |  |  |  |  |
| Food Insecurity | 0.77 (0.42, 1.43) | 0.84 (0.59, 1.18) | 1.07 (0.49, 2.37) | 0.81 (0.58, 1.14) |
| Lower Income | 1.11 (0.93, 1.31) | 1.16 (1.01, 1.32) | 1.08 (0.83, 1.39) | 1.20 (1.03, 1.39) |
| Lower Neighborhood Safety | 1.32 (0.93, 1.86) | 1.05 (0.78, 1.41) | 0.66 (0.39, 1.13) | 1.18 (0.88, 1.59) |
| Lower Wealth | 1.16 (0.92, 1.47) | 1.06 (0.92, 1.22) | 1.07 (0.85, 1.35) | 1.10 (0.96, 1.26) |
| Medicaid | 1.66 (0.95, 2.90) | 1.06 (0.75, 1.51) | 1.43 (0.65, 3.17) | 1.28 (0.92, 1.78) |
| Medicare | 0.99 (0.79, 1.25) | 0.87 (0.71, 1.07) | 0.95 (0.56, 1.61) | 0.98 (0.67, 1.42) |
| No Insurance | 0.98 (0.85, 1.13) | 0.99 (0.88, 1.12) | 0.92 (0.59, 1.44) | 0.80 (0.61, 1.05) |
| Received Food Stamps | 2.23 (1.28, 3.87) | 0.87 (0.50, 1.52) | 0.90 (0.31, 2.64) | 1.48 (1.02, 2.16) |
| Retired | 0.92 (0.75, 1.11) | 0.99 (0.87, 1.12) | 0.85 (0.48, 1.49) | 0.92 (0.70, 1.21) |
| Unemployed | 0.48 (0.11, 2.06) | 0.93 (0.34, 2.55) | 0.00 (0.00, 0.00) | 1.30 (0.66, 2.54) |
| *Health* |  |  |  |  |
| Arthritis | 0.98 (0.85, 1.13) | 1.01 (0.90, 1.14) | 1.02 (0.66, 1.58) | 1.04 (0.78, 1.38) |
| Back Pain | 1.05 (0.90, 1.24) | 0.96 (0.85, 1.09) | 1.36 (0.85, 2.17) | 0.91 (0.69, 1.21) |
| Cancer | 0.90 (0.74, 1.09) | 0.87 (0.73, 1.05) | 0.85 (0.42, 1.74) | 0.93 (0.58, 1.47) |
| Diabetes | 1.10 (0.91, 1.34) | 1.06 (0.86, 1.29) | 0.98 (0.62, 1.56) | 1.09 (0.77, 1.55) |
| Dizziness | 1.23 (0.97, 1.55) | 0.98 (0.82, 1.18) | 1.18 (0.70, 1.99) | 1.43 (1.00, 2.04) |
| Fatigue | 1.25 (0.96, 1.63) | 1.05 (0.90, 1.23) | 0.91 (0.41, 2.02) | 1.32 (0.96, 1.81) |
| Headaches | 1.09 (0.74, 1.61) | 1.24 (0.99, 1.55) | 1.13 (0.64, 2.00) | 1.38 (0.90, 2.12) |
| Heart Problems | 1.03 (0.87, 1.22) | 1.03 (0.88, 1.20) | 0.60 (0.31, 1.15) | 1.14 (0.83, 1.56) |
| Hypertension | 0.98 (0.85, 1.14) | 1.03 (0.92, 1.16) | 0.88 (0.56, 1.36) | 1.30 (0.98, 1.73) |
| Lower SR Health | 1.15 (0.97, 1.36) | 1.09 (0.95, 1.25) | 1.01 (0.66, 1.53) | 1.30 (0.96, 1.74) |
| Lower SR Hearing | 1.21 (1.03, 1.43) | 1.06 (0.94, 1.19) | 1.17 (0.73, 1.86) | 0.89 (0.65, 1.22) |
| Lower SR Vision | 1.11 (0.96, 1.28) | 1.08 (0.97, 1.21) | 1.25 (0.73, 2.15) | 1.03 (0.78, 1.37) |
| Lung Disease | 0.88 (0.64, 1.19) | 1.09 (0.83, 1.42) | 1.21 (0.48, 3.06) | 1.45 (0.78, 2.70) |
| Pain | 1.12 (0.93, 1.35) | 1.07 (0.94, 1.22) | 1.07 (0.61, 1.86) | 1.15 (0.84, 1.56) |
| Psychiatric Illness | 1.36 (0.96, 1.93) | 1.16 (0.94, 1.43) | 0.84 (0.25, 2.77) | 1.25 (0.72, 2.15) |
| Short of Breath | 1.13 (0.89, 1.42) | 1.01 (0.85, 1.20) | 1.25 (0.71, 2.17) | 1.17 (0.79, 1.73) |
| Stroke | 1.22 (0.93, 1.61) | 1.13 (0.90, 1.41) | 1.05 (0.49, 2.27) | 0.97 (0.52, 1.81) |
| Wheezing | 1.13 (0.90, 1.41) | 1.03 (0.83, 1.28) | 0.87 (0.43, 1.78) | 1.16 (0.77, 1.73) |
| *Behaviors* |  |  |  |  |
| Active Smoker | 1.15 (0.89, 1.50) | 1.06 (0.85, 1.32) | 0.96 (0.54, 1.69) | 1.15 (0.70, 1.88) |
| Ever Smoked | 1.05 (0.89, 1.23) | 1.02 (0.90, 1.14) | 1.14 (0.66, 1.99) | 1.06 (0.81, 1.39) |
| Heavy Alcohol Use | 0.93 (0.72, 1.20) | 1.13 (0.76, 1.69) | 0.96 (0.43, 2.13) | 0.85 (0.30, 2.39) |
| Higher BMI | 0.97 (0.79, 1.19) | 0.98 (0.85, 1.12) | 0.89 (0.53, 1.52) | 0.97 (0.78, 1.20) |
| Low/No Vigorous Physical Activity | 1.06 (0.92, 1.23) | 1.03 (0.91, 1.16) | 0.91 (0.58, 1.40) | 1.01 (0.77, 1.33) |
| *Social Connections* |  |  |  |  |
| Ever Divorced | 0.99 (0.80, 1.21) | 1.10 (0.93, 1.30) | 0.96 (0.58, 1.56) | 1.17 (0.87, 1.56) |
| Ever Widowed | 0.94 (0.76, 1.16) | 1.00 (0.89, 1.12) | 0.96 (0.57, 1.60) | 0.95 (0.72, 1.25) |
| Less Religious | 0.91 (0.79, 1.04) | 0.92 (0.81, 1.05) | 0.92 (0.51, 1.69) | 0.85 (0.53, 1.37) |
| Lonely | 1.03 (0.79, 1.33) | 1.14 (0.99, 1.32) | 1.57 (1.04, 2.38) | 1.23 (0.88, 1.72) |
| No Friends Nearby | 0.98 (0.82, 1.16) | 0.94 (0.82, 1.08) | 1.00 (0.62, 1.62) | 1.00 (0.73, 1.37) |
| No Relatives Nearby | 1.09 (0.93, 1.27) | 1.09 (0.96, 1.22) | 0.98 (0.58, 1.65) | 1.07 (0.81, 1.40) |
| Not Married/Partnered | 1.02 (0.81, 1.27) | 0.98 (0.87, 1.10) | 1.01 (0.64, 1.58) | 0.98 (0.72, 1.32) |
| *Genetic* |  |  |  |  |
| Higher AD PGS | 1.03 (0.89, 1.18) | 1.02 (0.91, 1.13) | 0.80 (0.38, 1.70) | 1.33 (0.75, 2.36) |
| Higher Coronary Artery Disease PGS | 0.96 (0.84, 1.11) | 1.01 (0.89, 1.14) | 0.72 (0.48, 1.07) | 1.09 (0.87, 1.36) |
| Higher Diabetes PGS | 1.02 (0.89, 1.18) | 1.02 (0.91, 1.14) | 0.70 (0.41, 1.20) | 0.89 (0.62, 1.27) |
| Higher Myocardial Infarction PGS | 0.94 (0.82, 1.08) | 1.03 (0.91, 1.17) | 0.72 (0.47, 1.11) | 0.83 (0.63, 1.09) |
| Higher Parity PGS | 0.94 (0.82, 1.08) | 1.01 (0.90, 1.14) | 0.79 (0.52, 1.20) | 0.89 (0.68, 1.16) |
| Lower Age at First Birth PGS | 1.01 (0.88, 1.17) | 1.00 (0.89, 1.13) | 1.11 (0.74, 1.66) | 1.01 (0.80, 1.28) |
| Lower Age at Menarche PGS | — | 1.01 (0.90, 1.13) | — | 0.96 (0.73, 1.26) |
| Lower Age at Menopause PGS | — | 1.03 (0.92, 1.15) | — | 0.93 (0.72, 1.19) |
| Lower Education PGS | 0.98 (0.86, 1.13) | 1.02 (0.91, 1.14) | 0.81 (0.53, 1.25) | 0.89 (0.69, 1.15) |
| Lower General Cognition PGS | 0.95 (0.82, 1.10) | 1.05 (0.93, 1.18) | 0.80 (0.54, 1.20) | 1.02 (0.79, 1.30) |
| Lower Height PGS | 0.97 (0.84, 1.11) | 1.02 (0.91, 1.14) | 0.71 (0.43, 1.15) | 1.05 (0.77, 1.42) |
| Lower Longevity PGS | 0.98 (0.84, 1.14) | 1.00 (0.89, 1.12) | 1.11 (0.83, 1.49) | 1.00 (0.78, 1.28) |

**S1 Appendix. Variable Construction.**

Sociodemographic characteristics.

Respondents were classified as *Childless* (1) if they reported never having children compared to reporting at least one child (-1).

Respondents were classified as *Foreign Born* if they reported being born outside of the United States (1) compared to being born in the US (-1).

Among respondents who reported having at least one child, *Higher Age at Last Birth* was a standardized measure (with mean zero, standard deviation one) of the respondent’s age when they last gave birth. Among respondents who were classified as childless, H*igher Age at Last Birth* was set to zero.

*Higher Parity* was defined as the number of children the respondent reported ever having. Values were reverse coded and standardized such that higher parity reflected higher risk.

Among respondents who reported having at least one child, *Lower Age at First Birth* was a standardized measure (with mean zero, standard deviation one) of the respondent’s age when they first gave birth. Values were reverse coded such that younger ages reflected higher risk. Among respondents who were classified as childless, *Lower Age at First Birth* was set to zero.

Respondents were classified as *Southern Born* if they reported being born in the South Atlantic, Eastern South Central, or Western South Central Census Divisions of the United States (1) compared to being born outside of these divisions (-1).

Respondents who served in the military were classified as a *Veteran* (1) compared to not (-1).

Early-Life Characteristics.

Respondent’s years of completed education were reverse coded and standardized with mean zero and standard deviation one to create the variable *Lower Education***.** This same process was used to create *Lower Father's Education* and *Lower Mother's Education*.

*Lower Father Occupational Status* was scored by categorizing the respondent’s father’s occupation as (1) Executives and managers, (2) Professional specialty, (3) Sales and administration, (4) Protection services and armed forces, (5) Cleaning, building, food preparation, and personal services, and (6) Production, construction, and operation occupations. Scores were then standardized with mean zero and standard deviation one. Scores were then standardized with mean zero and standard deviation one.

Respondents self-reported their overall health from birth to age 16 as excellent, very good, good, fair, and poor. Responses were scored such that higher values corresponded to worse health and then standardized with mean zero and standard deviation one to generate the variable *Lower SR Childhood Health.*

Respondents self-reported their family’s financial well-being from birth to age 16 as being pretty well off financially, about average, or poor. Responses were scored such that higher values corresponded to lower socioeconomic status (SES) and then standardized with mean zero and standard deviation one to generate the variable *Lower SR Childhood SES.*

Economic Characteristics

*Food Insecurity* was scored as binary (-1/1), with 1 indicating the respondent had reported not having enough money to buy the food they needed over the two year prior to their interview in 2000, and -1 indicating the respondent had enough money to buy the food they needed.

Income was measured in nominal dollars during the respondent’s interview in 2000 and is the sum of all income in a household, including: respondent’s and spouse’s wage/salary income, bonuses/overtime pay/commissions/tips, 2nd job or military reserve earnings, professional practice or trade income; household business or farm income, self-employment earnings, business income, gross rent, dividend and interest income, trust funds or royalties, and other asset income; respondent’s and spouse’s income from all pensions and annuities; respondent’s and spouse’s total Social Security income (including that which is and is not received due to disability); respondent’s and spouse’s income from unemployment and worker’s compensation; respondent’s and spouse’s income from veterans’ benefits, welfare, and food stamps; and alimony, other income, and lump sums from insurance, pension, and inheritance at the household level. This value was then log-transformed, reverse coded, and standardized with mean zero and standard deviation one to generate the variable *Lower Income*.

Respondents reported the safety of their neighborhood. Response options were excellent, very good, good, fair, and poor. Responses were coded such that higher values corresponded to *Lower Neighborhood Safety* and then standardized with mean zero and standard deviation one.

Wealth was measured in nominal dollars during the respondent’s interview in 2000 as the sum of all wealth components (except secondary home) less all debt. This value was then log-transformed, reverse coded, and standardized with mean zero and standard deviation one to generate the variable *Lower Wealth.*

*Medicaid* was coded as binary (-1/1), with 1 indicating the respondent reported being covered by Medicaid during their interview in 2000 and -1 indicating otherwise.

*Medicare* was coded as binary (-1/1), with 1 indicating the respondent reported being covered by Medicare during their interview in 2000 and -1 indicating otherwise.

*No Insurance* was coded as binary (-1/1), with 1 indicating the respondent reported being uninsured during their interview in 2000 and -1 indicating otherwise.

*Food Stamps* was scored as binary (-1/1), with 1 indicating the respondent or a family member living with them received government food stamps over the two year prior to their interview in 2000, and -1 indicating otherwise.

*Retired* was coded as binary (-1/1), with 1 indicating the respondent reported being retired during their interview in 2000 and -1 indicating otherwise.

*Unemployed* was coded as binary (-1/1), with 1 indicating the respondent reported was not working for pay while actively looking for a job in the last four weeks prior to their interview in 2000 and -1 indicating otherwise.

Behaviors

*Active Smoker* was scored as binary (-1/1), with 1 indicating the respondent reported being a current smoker and -1 otherwise.

*Ever Smoked* was scored as binary (-1/1), with 1 indicating the respondent reported ever smoking and -1 indicating otherwise.

*Heavy Alcohol Use* was scored as binary (-1/1), with 1 indicating the respondent reported drinking three or more drinks per day on days they drank and -1 otherwise.

Body mass index (BMI) was calculated by dividing the respondent's weight by their height-squared and then standardizing with mean zero and standard deviation one. Higher scores reflected *Higher BMI***.**

*Low/No Vigorous Activity* was scored as binary (-1/1), with -1 indicating the respondent reported completing vigorous activity (including, for example, sports, heavy housework, or a job that involves physical labor) three or more times per week over the 12 month period prior to their interview in 2000 and 1 indicating otherwise.

Health Characteristics

All binary health characteristics were coded as 1 if the respondent self-reported the condition or was otherwise coded as -1.

Respondents were asked whether a doctor ever told them that they had *Arthritis* (arthritis or rheumatism), *Cancer* (cancer or a malignant tumor, excluding minor skin cancers), *Diabetes* (diabetes or high blood sugar), *Heart Problems* (heart attack, coronary heart disease, angina, congestive heart failure, or other heart problems), *Hypertension* (high blood pressure or hypertension), *Lung Disease* (chronic lung disease such as chronic bronchitis or emphysema), *Psychiatric Illness* (any emotional, nervous, or psychiatric problems), *Stroke***.**

Respondents were also asked to report whether they had persistent *Back Pain, Dizziness, Fatigue, Headaches*, were *Short of Breath, Wheezing*, and whether they were often troubled with *Pain* over the 12 month period prior to the interview in 2000.

Respondents self-reported their overall health (*SR Health*), hearing (*SR Hearing*), and vision (*SR Vision*). Response options were excellent, very good, good, fair, and poor. Responses were coded such that higher values corresponded to worse health/hearing/vision and then standardized with mean zero and standard deviation one.

Social Ties

*Ever Divorced* was coded as binary (-1/1), with 1 indicating the respondent reported being divorced or separated and -1 indicating the respondent never reported being divorced no separated.

*Ever Widowed* was coded as binary (-1/1), with 1 indicating the respondent reported being widowed and -1 indicating the respondent never reported being widowed.

Respondents reported whether religion was very important, somewhat important, or not too important in their lives. Responses were coded such that higher values corresponded to being *Less Religious* and then standardized with mean zero and standard deviation one.

Respondents reported whether they felt *Lonely* much of the week prior to their interview in 2000. Responses were scored (-1/1), with 1 indicating a response of “yes” and -1 indicating a response of “no.”

Respondents reported whether they had any good friends or relatives living in their neighborhood. Responses were used to create binary indicators for *No Friends Nearby* (1 if “no”; -1 if “yes”) and *No Relatives Nearby* (1 if “no”; -1 if “yes”).

*Not Married/Partnered* was coded as binary (-1/1), with 1 indicating the respondent was not married and not partnered at the time of their interview in 2000 and -1 indicating otherwise.

Genetic

All polygenic scores (PGSs) were residualized by regressing the PGS on the first 10 single nucleotide polymorphisms principal components, computing the residuals from the predictions, and standardizing the residual values to a normal distribution with mean zero and standard deviation 1. Some PGSs were reverse coded as described below. Detailed information on sample selection, consent procedures, and assay processes are provided by the Health and Retirement study (HRS) investigators [1].

*Higher AD PGS* represents the Alzheimer's disease PGS (with apolipoprotein [ApoE] status variants, rs7412 and rs429358) [2].

*Higher Coronary Artery Disease PGS* represents the PGS for coronary artery disease which was created by HRS investigators using results from a 2011 study conducted by the Coronary Artery Disease Genome wide Replication and Meta-analysis Consortium [3].

*Higher Diabetes PGS* represents the type 2 diabetes PGS which was created by HRS investigators using GWAS meta-analysis results from a 2012 study conducted by the Diabetes Genetics Replication and Meta-analysis Consortium [4].

*Higher Myocardial Infarction PGS* represents the myocardial infarction PGS which was created by HRS investigators using 2015 results from a subgroup analysis of coronary artery disease conducted by the Coronary Artery Disease Genome wide Replication and Meta-analysis Consortium [5].

*Higher Parity PGS* represents a PGS for the number of children ever born created by HRS investigators from a 2016 study conducted by the Sociogenome Consortium [6]. Values were coded such that higher PGSs corresponded to higher genetic propensity for higher parity levels.

*Lower Age at First Birth PGS* represents a PGS for age at first birth created by HRS investigators from a 2016 study conducted by the Sociogenome Consortium [6]. Values were reverse coded such that higher PGSs corresponded to lower genetic propensity for younger age at first birth.

*Lower Age at Menarche PGS* represents a PGS for age at menarche created by HRS investigators from a 2014 study conducted by the Reproductive Genetics (ReproGen) Consortium [7]. Values were reverse coded such that higher PGSs corresponded to lower genetic propensity for younger age at menarche.

*Lower Age at Menopause PGS* represents a PGS for age at menopause created by HRS investigators from a 2014 study conducted by the Reproductive Genetics Consortium [8]. Values were reverse coded such that higher PGSs corresponded to lower genetic propensity for younger age at menopause.

*Lower Education PGS* represents a PGS for educational attainment which was created by HRS investigators using results from a 2018 study by the Social Science Genetic Association Consortium [9]. Values were reverse coded such that higher PGSs corresponded to lower genetic propensity for higher educational attainment

*Lower General Cognition PGS* represents a PGS for general cognition created by HRS investigators from the Cohorts for Heart and Aging Research in Genomic Epidemiology Consortium, 2015 [10]. Values were reverse coded such that higher PGSs corresponded to lower genetic propensity for higher cognitive functioning

*Lower Height PGS* represents a PGS for height created by HRS investigators from a 2014 study conducted by the Genetic Investigation of Anthropometric Traits Consortium [11]. Values were reverse coded such that higher PGSs corresponded to lower genetic propensity for taller height

*Lower Longevity PGS* represents a PGS for longevity created by HRS investigators from the Cohorts for Heart and Aging Research in Genomic Epidemiology Consortium, 2015 [12]. Values were reverse coded such that higher PGSs corresponded to lower genetic propensity for higher longevity

**S2 Appendix. Statistical Methods.**

**Fine-Gray Estimator**

The Kaplan-Meier (KM) estimator is a non-parametric statistical technique that was introduced as a way to study time-to-event data with applications to mortality in the presence of attrition (e.g., study drop-out, loss to follow-up) [13]. In the setting for which it was developed (i.e., studies of mortality which may have incomplete follow-up), the required assumption of uninformative censoring—or that subjects who are lost to follow-up (i.e., censored) have the same survival prospects as those who continue to be followed–is likely to be met within the KM framework. It is assumed that these censored subjects for whom we do not observe an event (i.e., death) remain at risk of the event.

However, when using the KM estimator to examine a health outcome, mortality becomes another way in which subjects may be censored. In this application, subjects will be censored of they die prior to developing the health outcome of interest yet they will be considered “at risk” in the same way a subject who was lost to follow-up would be. In this case, the KM estimator does not account for the competing risk of mortality and will yield biased estimates.

The Fine-Gray model was introduced to account for competing risks in these settings [14]. Whereas in the KM framework a subject who died prior to experiencing the health outcome of interest would be censored, the Fine-Gray classification scheme uses weights to represent the conditional probability of an event of interest for subjects who experienced the competing event, carrying these subjects forward in the analysis rather than censoring them.

**Random Forest Algorithm**

To complement our analyses using the Fine-Gray model, we used a data-driven classification scheme referred to as random forest competing risks survival analysis [15, 16]. Random forest is a non-parametric, ensemble machine-learning algorithm which iteratively bifurcates a dataset based on predictor variables over a number of permutations set by the user and then ranks the importance of each predictor based on its ability to “split” the data [17]. The basis for this algorithm is fitting multiple decision trees on the data and pooling them together which overcomes the limitations of singular classification and regression tree (CART) classification schemees. To do this, the algorithm repeatedly draws bootstrap samples from the analytic sample and a random selection of predictors to grow a predetermined number of decision trees (i.e., a forest) set by the user across which results are pooled. A training data set consisting of *n* of N cases (approximately two-thirds of the original sample) is generated for each of *k* decision trees and the remaining cases (one third of the original sample) are used as test data to estimate the out of bag (OOB) classification error. A random sample *m* of M predictors is selected at each node and the one predictor that best discriminates discrepancies in the outcome is chosen for that particular split. As a result, the root node of each decision tree represents the strongest predictor and the splits that follow are based on the successively strongest predictors. A final classification is made using a majority of votes across all trees.

**References**

1. Ware E, Schmitz L, Gard A, Faul J. HRS Polygenic Scores—Release 3: 2006–2012 Genetic Data. Ann Arbor: Survey Research Center, University of Michigan. 2018.

2. Lambert J-C, Ibrahim-Verbaas CA, Harold D, Naj AC, Sims R, Bellenguez C, et al. Meta-analysis of 74,046 individuals identifies 11 new susceptibility loci for Alzheimer's disease. Nature genetics. 2013;45(12):1452.

3. Schunkert H, König IR, Kathiresan S, Reilly MP, Assimes TL, Holm H, et al. Large-scale association analysis identifies 13 new susceptibility loci for coronary artery disease. Nature genetics. 2011;43(4):333.

4. Morris AP, Voight BF, Teslovich TM, Ferreira T, Segre AV, Steinthorsdottir V, et al. Large-scale association analysis provides insights into the genetic architecture and pathophysiology of type 2 diabetes. Nature genetics. 2012;44(9):981.

5. Nikpay M, Goel A, Won H-H, Hall LM, Willenborg C, Kanoni S, et al. A comprehensive 1000 Genomes–based genome-wide association meta-analysis of coronary artery disease. Nature genetics. 2015;47(10):1121.

6. Barban N, Jansen R, De Vlaming R, Vaez A, Mandemakers JJ, Tropf FC, et al. Genome-wide analysis identifies 12 loci influencing human reproductive behavior. Nature genetics. 2016;48(12):1462-72.

7. Perry JR, Day F, Elks CE, Sulem P, Thompson DJ, Ferreira T, et al. Parent-of-origin-specific allelic associations among 106 genomic loci for age at menarche. Nature. 2014;514(7520):92-7.

8. Day FR, Ruth KS, Thompson DJ, Lunetta KL, Pervjakova N, Chasman DI, et al. Large-scale genomic analyses link reproductive aging to hypothalamic signaling, breast cancer susceptibility and BRCA1-mediated DNA repair. Nature genetics. 2015;47(11):1294.

9. Lee JJ, Wedow R, Okbay A, Kong E, Maghzian O, Zacher M, et al. Gene discovery and polygenic prediction from a 1.1-million-person GWAS of educational attainment. Nature genetics. 2018;50(8):1112.

10. Davies G, Armstrong N, Bis JC, Bressler J, Chouraki V, Giddaluru S, et al. Genetic contributions to variation in general cognitive function: a meta-analysis of genome-wide association studies in the CHARGE Consortium (N= 53 949). Molecular psychiatry. 2015;20(2):183.

11. Wood AR, Esko T, Yang J, Vedantam S, Pers TH, Gustafsson S, et al. Defining the role of common variation in the genomic and biological architecture of adult human height. Nature genetics. 2014;46(11):1173.

12. Broer L, Buchman AS, Deelen J, Evans DS, Faul JD, Lunetta KL, et al. GWAS of longevity in CHARGE Consortium confirms APOE and FOXO3 candidacy. Journals of Gerontology Series A: Biomedical Sciences and Medical Sciences. 2014;70(1):110-8.

13. Berry SD, Ngo L, Samelson EJ, Kiel DP. Competing risk of death: an important consideration in studies of older adults. Journal of the American Geriatrics Society. 2010;58(4):783-7. Epub 2010/03/22. doi: 10.1111/j.1532-5415.2010.02767.x. PubMed PMID: 20345862.

14. Fine JP, Gray RJ. A proportional hazards model for the subdistribution of a competing risk. Journal of the American statistical association. 1999;94(446):496-509.

15. Ishwaran H, Kogalur U. randomForestSRC: Random Forests for Survival, Regression and Classification (RF-SRC). R package version 1.4. 0. 2014.

16. Ishwaran H, Kogalur UB, Blackstone EH, Lauer MS. Random survival forests. The annals of applied statistics. 2008;2(3):841-60.

17. Breiman L. Random forests. Machine learning. 2001;45(1):5-32.
